# Evolution of the Natural Transformation Protein, ComEC, in Bacteria

**DOI:** 10.3389/fmicb.2018.02980

**Published:** 2018-12-12

**Authors:** Zachary T. Pimentel, Ying Zhang

**Affiliations:** Department of Cell and Molecular Biology, College of the Environment and Life Sciences, University of Rhode Island, Kingston, RI, United States

**Keywords:** natural transformation, natural competence, ComEC, horizontal gene transfer, *Competence*, *DUF4131*, *Lactamase_B*

## Abstract

Natural transformation enables the incorporation of exogenous DNA into host genomes and plays a fundamental role in the evolution of microbial populations. At the center of the natural transformation machinery, the ComEC protein mediates DNA import and serves potential functions in DNA recognition and single strand degradation. Despite its importance, the evolution of ComEC is not fully understood. Here, we aim to fill this knowledge gap by surveying putative ComEC proteins across 5,574 bacteria that span diverse phyla. We first derived the presence of a universal, core *Competence* domain through the analysis of ComEC proteins from known naturally competent species. Then, we followed this observation to identify *Competence* domain containing proteins (CDCPs) from all bacteria and used CDCPs as putative ComEC proteins for evolutionary analysis. A near universal presence of CDCPs was revealed, with 89% of the proteomes and 96% of the genomes encoding a single CDCP or a CDCP-like fragment. Two domains, *DUF4131* and *Lactamase_B*, were found to commonly co-occur with the *Competence* domain. Ancestral state reconstruction of CDCPs over the bacterial species phylogeny suggested an origin of a *Competence*-only domain profile, while multiple gains and losses of the *DUF4131* and *Lactamase_B* domains were observed among diverse bacterial lineages.

## Introduction

Natural transformation (NT) is a process by which exogenous DNA is imported and incorporated into a genome. It is an important mechanism of horizontal gene transfer and as such enables DNA repair and the acquisition of new functions, such as antibiotic resistance and nutrition utilization (Ambur et al., 2016). Due to the diverse regulatory mechanisms involved in NT, natural competence is often difficult to initiate in laboratory conditions (Dubnau, 1991; Meibom et al., 2005). To date, only around 80 naturally competent bacterial strains have been confirmed in the literature (Lorenz and Wackernagel, 1994; Johnsborg et al., 2007; Johnston et al., 2014).

The NT process is mediated by a complex machinery of proteins via multiple steps. In gram-negative bacteria, initiation of outer membrane double-stranded DNA (dsDNA) uptake into the periplasmic space involves proteins related to components of the type IV pilus, the type II secretion system (Chen and Dubnau, 2004), or the type IV secretion system (Hofreuter et al., 2001). The dsDNA then binds to ComEA, which is hypothesized to pull the transforming DNA into the periplasm and is present in both gram-positive and gram-negative species (Bergé et al., 2002; Matthey and Blokesch, 2016). One strand of the dsDNA is then subject to degradation, for example, by the nuclease EndA in *Streptococcus pneumoniae* (Puyet et al., 1990) or by a putative nuclease domain of the ComEC protein in *Bacillus subtilis* (Baker et al., 2016). The single-stranded DNA (ssDNA) retained from degradation of its complement strand is then transported by a conserved ComEC protein into the cytosol (Draskovic and Dubnau, 2005; Baker et al., 2016). In a following step, the cytosolic ssDNA is recognized by DprA and recombined into the genomic DNA by RecA (Mortier-Barrière et al., 2007).

The composition of NT machinery varies among naturally competent species (Johnston et al., 2014). Several proteins, such as RecA and PilA, have known functions outside of the NT process (Taha et al., 1992; Cox, 2007). The DNA Processing Protein A (DprA) and the Competence protein EC (ComEC), however, are shown to be essential for NT (Friedrich et al., 2001; Yeh et al., 2003; Wilharm et al., 2013; Mell and Redfield, 2014). While DprA is responsible for binding ssDNA in the cytosol (Mortier-Barrière et al., 2007; Quevillon-Cheruel et al., 2012), ComEC is predicted to mediate multiple steps including the DNA binding, single strand degradation (to expose the other strand), and ssDNA membrane translocation (Baker et al., 2016). Further, while homologs of DprA and RecA are present in archaea, ComEC appears to be absent among the few naturally transformable archaeal species, indicating distinct DNA import mechanisms between bacteria and archaea (Lipscomb et al., 2011; van Wolferen et al., 2016).

ComEC proteins have been identified among diverse bacterial phyla, including candidate phyla that lack cultured representatives (Kantor et al., 2013; Mell and Redfield, 2014). The ComEC protein of *B. subtilis* contains three Pfam domains, a domain of unknown function (*DUF4131*), a transmembrane competence domain (*Competence*), and a metallo-beta-lactamase domain (*Lactamase_B*), ordered accordingly from the N-terminus to the C-terminus of the protein (Baker et al., 2016). The *DUF4131* domain contains a putative OB fold that is predicted to have functions in nucleic acids binding (Baker et al., 2016), the *Competence* domain includes a set of core transmembrane helices that mediate the uptake of ssDNA (Draskovic and Dubnau, 2005), and the *Lactamase_B* domain belongs to a broad family of DNA and RNA nucleases that could be responsible for the degradation of a single strand in the dsDNA (Callebaut, 2002; Dominski, 2007; Baker et al., 2016).

While studies have been performed on the structure, function, and regulation of ComEC proteins among a small number of competent bacteria, little is known about the taxonomic distribution and domain evolution of this protein across a broader range of bacterial species. To fill this knowledge gap, here we report the identification of putative ComEC proteins (referred to as CDCPs) among 5,574 bacteria and provide a first ancestral state reconstruction of the protein among diverse bacterial lineages.

## Materials and Methods

### Collection and Domain Analysis of Known ComEC Proteins

A collection of ComEC proteins from strains experimentally confirmed to be capable of NT were obtained from the literature (Supplementary Table S1). The ComEC proteins were compared against the Pfam-A database, version 27.0 (Finn et al., 2016) using the *hmmscan* function in the HMMER package, version 3.1b2 (Eddy, 1998). Domain hits with an e-value of greater than 1 × 10^-5^ and a coverage of less than 70% of the Pfam-A models were removed. The remaining domain mappings were used to reconstruct the profiles of domain abundances for each ComEC protein.

### Identification and Domain Profiling of CDCPs

**FIGURE 1:**
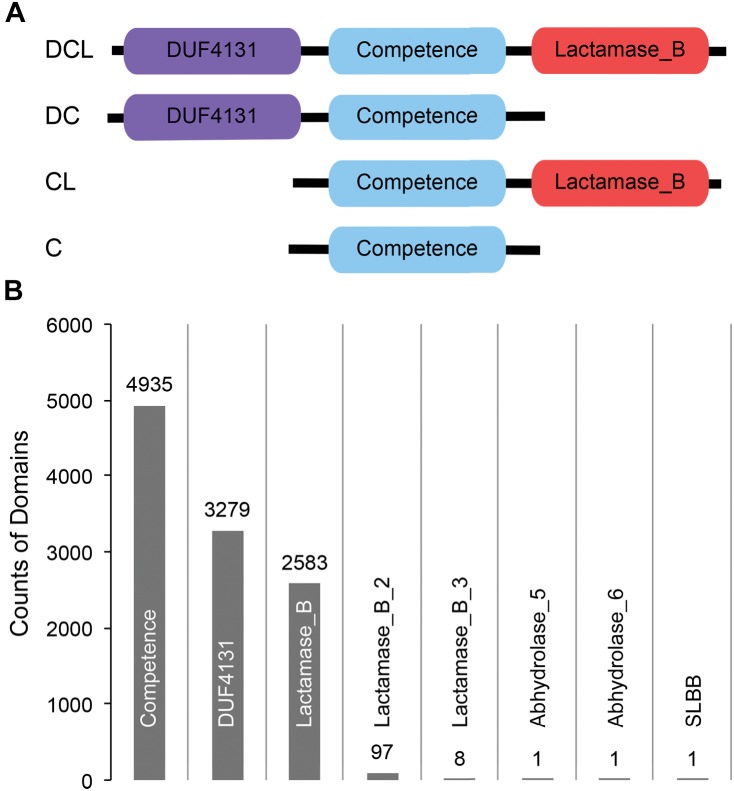
**(A)** Cartoon representation of the domain profiles identified from ComEC proteins of naturally competent bacterial strains. **(B)** Abundance of domains observed from a global search of CDCPs in 5,574 bacterial proteomes.

**FIGURE 2:**
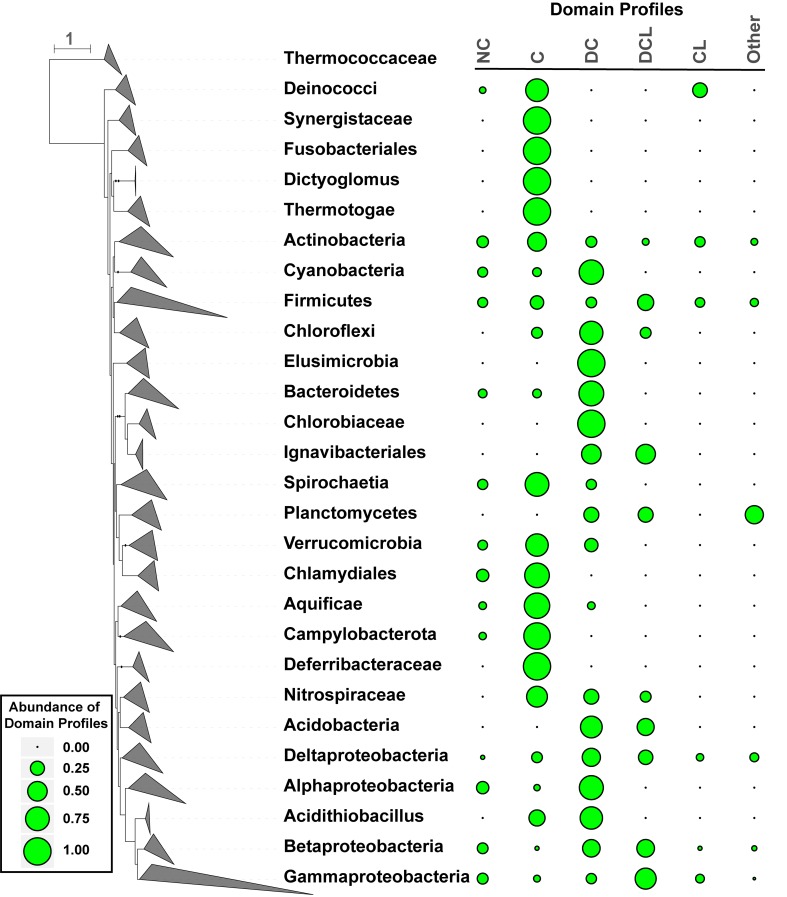
Distribution of CDCPs and their domain profiles in the bacterial species phylogeny. A dendrogram on the left represents the branching of different taxonomic groups, which were manually collapsed and represented as triangles following the convention of the iTol software (Letunic and Bork, 2016). The taxa names were shown on the right of the corresponding collapsed branches. A boxed legend on the lower left provides a scale for the bubble plot on the right, which represents the abundance values of different CDCP domain profiles for each taxon. The following profiles were considered: No CDCP (***NC***), *Competence* only (***C***), *DUF4131* and *Competence* (***DC***), *Competence* and *Lactamase_B* (***CL***), *DUF4131*, *Competence*, and *Lactamase_B* (***DCL***). All other profiles were combined into the ***Other*** group due to their low abundance.

A set of 5,574 complete bacterial genomes and their corresponding proteomes were downloaded from the NCBI RefSeq database (O’Leary et al., 2016) on October 25, 2016. Proteins containing the *Competence* domain (Pfam accession number: PF03772) were identified as CDCPs following the above-mentioned criteria of domain mapping, and the CDCPs were further analyzed to enumerate the co-occurrence of additional domains. Detailed information of all CDCPs were enlisted as individual rows in Supplementary Table S2. The occurrence of different domains was examined among all proteomes analyzed (Figure 1). In order to decrease biases associated with over sampled species when calculating the taxonomic distribution of the different profile types (Figure 2), each domain profile (i.e., combination of domains in a protein) was counted proportionally based on their frequencies among different strains of a species. For example, three distinct profiles were observed among different strains of *Escherichia coli* with an occurrence of 9, 13, and 177 strains for each profile. Hence, a relative count of 0.045, 0.065, and 0.889, respectively, were assigned to each of the profiles in *E. coli*.

### Genomic Analysis and Identification of Missing CDCPs

Genomic sequences of all bacterial strains were analyzed to search for potential CDCP coding regions or degenerated CDCP fragments that were not captured in the proteomic analysis. First, a query database was constructed by extracting nucleotide sequences that correspond to the CDCPs identified from the proteome-wide analysis. Then, subsequences of the genes that specifically encoded the *Competence* domains were searched against the database of 5,574 complete genomes using the *blastn* function in the BLAST+ package, version 2.2.31 (Camacho et al., 2009). A threshold of e-value less or equal to 1 × 10^-5^, query coverage 30% or greater, and identity greater than or equal to 60% were used in the database search. Hits were identified among genomes that did not contain a CDCP in their corresponding proteomes (Supplementary Table S2).

### Reconstruction of the Bacterial Species Phylogeny

AMPHORA2 (Wu and Scott, 2012) was used to identify marker genes from the 2,373 representative proteomes selected from the reduction of oversampling biases (described in an above section). Species of the archaeal order Thermococcales were used as an outgroup for the species phylogeny (39 proteomes). In cases where multiple copies of a marker were predicted in one genome, the protein with the lowest e-value was retained. Each identified marker gene was aligned and trimmed using the ‘-Trim’ option in the MarkerAlignTrim.pl script of AMPHORA2. The individual protein alignments were then concatenated into one alignment. The hybrid mode of RAxML version 8.2.1 (Stamatakis, 2014) was used to infer a phylogeny using the JTT substitution model and CAT model of evolution. The internal nodes of the phylogeny were labeled based on the lowest common ancestor (LCA) of the underlying leaves. The tree was collapsed manually in iTol (Letunic and Bork, 2016) and taxa that contained a single representative in the dataset were removed to facilitate the visualization of major taxonomic groups (Supplementary Data Sheet S1). The distribution of different CDCP domain profiles in Figure 2 was calculated based on the fraction of relative counts among all strains represented in each collapsed branch.

### Ancestral State Reconstruction

The evolution of domains in CDCPs was inferred through reconstructing ancestral domain compositions across the species phylogeny. The three most abundant Pfam domains, *Competence*, *DUF4131* (Pfam accession PF13567), and *Lactamase_B* (Pfam accession PF00753), were considered in the ancestral state reconstruction. A total of five different states were assigned to each leaf of the species phylogeny based on the presence or absence of each domain, these included (1) No CDCP (***NC***); (2) *Competence* only (***C***); (3) *Competence* and *Lactamase_B* (***CL***); (4) *DUF4131* and *Competence* (***DC***); and (5) *DUF4131, Competence*, *and Lactamase_B* (***DCL***). Ancestral state reconstruction was performed with the ‘ace’ function from the APE package, version 5.1 (Paradis et al., 2004) in R, version 3.4.2 (R Core Team, 2017) with the ‘discrete’ type and equal rates (‘ER’) model. Subsequently, the posterior probabilities of each state at each internal node was overlaid onto the species phylogeny using the Phytools package, version 0.6-44 (Revell, 2012) in R, version 3.4.2 (R Core Team, 2017).

### Reconstruction of Protein Domain Phylogenies

Protein sequences of the *Competence*, *DUF4131*, and *Lactamase_B* domains were extracted from entire CDCP sequences according to alignment positions of the respective Pfam domains. The extracted sequences were aligned using MUSCLE, version 3.8.31 (Edgar, 2004). Individual domain phylogenies were constructed using the hybrid mode of RAxML, version 8.2.1 (Stamatakis, 2014) with the JTT substitution model and the CAT model of evolution. The trees were midpoint rooted with taxonomic information mapped to the leaves. Due to the redundant nature of NCBI protein identifiers across proteomes, if one protein identifier was associated with multiple proteomes the corresponding leaf identifier was randomly assigned to one of the lineages. Additional verifications were performed to ensure that such redundant protein identifiers only occurred in closely related bacterial strains and hence did not influence the derivation of LCAs.

### Comparison of Evolutionary Distances

**FIGURE 3:**
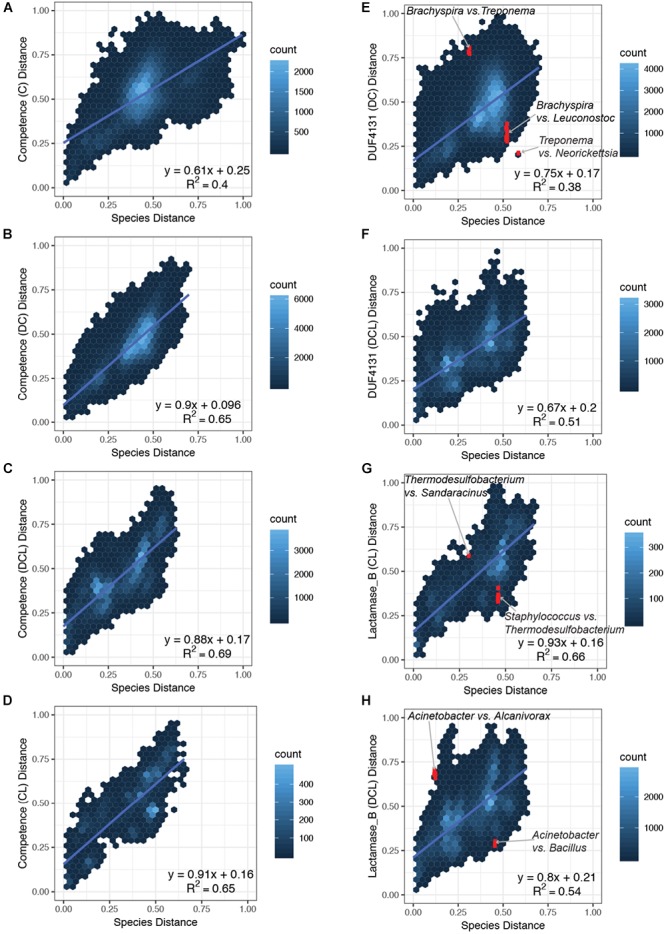
Comparison of pairwise distances in the species versus the domain phylogenies. Normalized distances from the species phylogeny (*x*-axis) is plotted against the normalized distances from the domain phylogenies (*y*-axis) for each subset of proteins that carry the same CDCP profile. Four subsets that represent the ***C***, ***DC***, ***DCL***, and ***CL*** profiles of the *Competence* domain phylogeny were plotted in panels **(A–D)**, respectively; two subsets that represent the ***DC*** and ***DCL*** profiles of the *DUF4131* domain phylogeny were plotted in **E,F**; and two subsets that represent the ***CL*** and ***DCL*** profiles of the *Lactamase_B* domain phylogeny were plotted in **G,H**. The following pairs are highlighted in red (and labeled in the corresponding plots): **(E)** *Brachyspira* vs. *Leuconostoc*, *Brachyspira* vs. *Treponema*, and *Treponema* vs. *Neorickettsia*; **(G)** *Staphylococcus* vs. *Thermodesulfobacterium* and *Thermodesulfobacterium* vs. *Sandaracinus*; **(H)** *Acinetobacter* vs. *Bacillus* and *Acinetobacter* vs. *Alcanivorax*.

Pairwise phylogenetic distances were computed for leaves of the species phylogeny and the phylogenies of the *Competence*, *DUF4131*, and *Lactamase_B* domains with the ‘cophenetic’ function in R, version 3.4.2 (R Core Team, 2017). The distances in the domain phylogenies were normalized by the maximum pairwise distances found in each tree. The distances in the species phylogeny were similarly normalized, but the maximum pairwise distance among all CDCP-containing strains were used as the denominator for the normalization. Pairs of strains that both have the ***C***, ***DC***, ***CL***, or ***DCL*** profiles from the *Competence* tree along with pairs of strains that both have the ***DC*** or ***DCL*** and ***CL*** or ***DCL*** profiles in the *DUF4131* and *Lactamase_B* trees, respectively, were identified (Figure 3). Linear models were constructed using the ‘lm’ function in R, version 3.4.2 (R Core Team, 2017) to correlate the species distances with the domain distances for each subplot in Figure 3.

## Results

### Domain Composition of Known ComEC Proteins

A list of 51 naturally competent bacterial strains were identified from the literature (Johnston et al., 2014 and references therein). These strains span eight different phyla, and each strain encodes a single ComEC protein varying in length from 417 to 872 amino acids (Supplementary Table S1). Three distinct protein domains, *DUF4131*, *Competence*, and *Lactamase_B*, were observed based on sequence comparisons of the ComEC proteins to the Pfam database (Materials and Methods). While the *Competence* domain was universally conserved, the *DUF4131* and *Lactamase_B* domains appeared in some but not all ComEC proteins. Overall, four domain profiles were identified (Figure 1A). The most abundant was the ***DCL*** profile (24 occurrences), followed by the ***DC*** profile (14 occurrences). The least abundant were the ***CL*** (7 occurrences) and the ***C*** profiles (6 occurrences). The great diversity in domain compositions and sequence lengths of ComEC proteins prevents the global identification of the protein from comparison of full length sequences alone. However, due to the universal presence of the *Competence* domain among all known ComEC proteins, here we study the *Competence* domain containing proteins (CDCPs) and use them as a proxy to infer the distribution, domain profiles, and evolution of putative ComEC proteins among a diverse range of fully sequenced bacterial genomes.

### Abundance and Distribution of CDCPs in Bacteria

Complete proteomes of 5,574 bacteria were analyzed, where CDCPs were found in 4,934 proteomes. Of the 640 proteomes for which a CDCP was not identified, 409 were predicted to encode either complete or fragmented *Competence* domains in the genomic DNA (Supplementary Table S2). Hence, a near universal presence of the protein was observed among 89% of the proteomes and 96% of the genomes analyzed in this study. The remaining 231 strains for which a *Competence* domains was missing included representatives from the *Buchnera*, *Rickettsia*, *Blattabacterium*, *Mycoplasma*, and *Prochlorococcus* genera. These genera are typically obligate endosymbionts, intracellular pathogens, or small marine phytoplanktons, and they are known to have highly reduced genomes (Wernegreen and Moran, 1999; Moran, 2002; Kuo et al., 2009).

When present, only a single CDCP was found in every proteome except for the *Pseudarthrobacter sulfonivorans* strain Ar51, which contains two identified CDCPs in its proteome. However, one of the proteins (WP_058932413.1) appeared to be a truncated copy of the other (WP_058932395.1), as they share a sequence identity of 94% and the former is 330 amino acids shorter than the latter. In addition, all identified CDCPs were encoded in the chromosomes except for the strains *Xanthomonas sacchari* R1 and *Prevotella* sp. oral taxon 299 F0039, where the CDCPs were encoded in plasmids. The occurrence of CDCPs varied among different taxa. For example, in the phyla Thermotogae, Fusobacteria, Chlorobi, and Chloroflexi, a CDCP was found in every strain. In contrast, a CDCP was missing among 10.7% and 36.6% of the strains in Actinobacteria and Tenericutes, respectively.

### Domain Variations in CDCPs

Besides the *DUF4131* and *Lactamase_B* domains found in known ComEC proteins, five additional domains were found to co-occur with the *Competence* domain through the global search of bacterial proteomes (Figure 1B). These included members of the beta-lactamase superfamilies *Lactamase_B_2* (Pfam accession PF12706) and *Lactamase_B_3* (Pfam accession PF13483), the alpha/beta hydrolase families *Abhydrolase_5* (Pfam accession PF12695) and *Abhydrolase_6* (Pfam accession PF12697), and the *SLBB* domain (Pfam accession PF10531) of the beta-grasp fold (Burroughs et al., 2007). The *DUF4131* and *Lactamase_B* remained to be the most dominant among all domains: of the 4,935 CDCPs identified, the *DUF4131* domain was in 66.4% (3,279 occurrences) and the Lactamase_B domain was in 52.3% (2,583 occurrences). In contrast, all other domains only had from one to 97 occurrences (Figure 1B).

By default, the collection of proteomes included an over-representation of certain species, such as *E. coli* (199 distinct proteomes), *Helicobacter pylori* (83 distinct proteomes), and *B. subtilis* (45 distinct proteomes). Substrains of these species may contain CDCPs with distinct domain profiles. For example, of the 199 distinct *E. coli* proteomes analyzed, nine encoded no CDCP (***NC***), 13 encoded CDCPs of the ***CL*** profile, and 177 encoded CDCPs of the ***DCL*** profile (Supplementary Table S2). To avoid biases introduced by oversampling of such species while still account for the diversity of domain profiles, a non-redundant set of 2,373 representative proteomes were identified that proportionally represents domain profiles among bacterial species (Materials and Methods).

A bacterial species phylogeny was constructed from protein marker sequences identified in the representative proteomes (Figure 2). The frequency of CDCP domain profiles within any given taxon was calculated by dividing the relative counts of each profile by the sum of relative counts for all proteomes in the taxon (Materials and Methods). Six distinct profiles were considered. Besides the dominant ***C***, ***DC***, ***DCL***, and ***CL*** profiles, strains that encode any other domains were classified into a group named ***Other***, and strains that encode no CDCP were classified as ***NC***.

The analysis of domain distributions (Figure 2) revealed a great diversity among different taxa. While the Actinobacteria, Firmicutes, Betaproteobacteria, and Deltaproteobacteria included a relatively even proportion of all profiles, other taxa demonstrated preferences to one or a few profiles. Among all CDCPs, the ***C*** profile appeared to be the most widely distributed across a diverse range of bacterial phyla. It was dominant (with a frequency of over 0.50) in Aquificae, Campylobacterota, Thermotogae, Deinococcus-Thermus, Chlamydiae, and Spirochaetes. The ***DC*** profile, one of the most abundant among all profiles, was the main representative in Cyanobacteria, Bacteroidetes, and Alphaproteobacteria. The ***DCL*** profile occurred in multiple taxa, but was only dominant in the Gammaproteobacteria. Additionally, a dominance of the ***CL***, ***NC***, and ***Other*** profiles was not observed in any taxa, and the main occurrences of these profiles were in Actinobacteria, Firmicutes, and Gammaproteobacteria.

### Evolution of Domain Compositions in CDCPs

An ancestral state reconstruction of the domains indicated the ***C*** profile as the most parsimonious ancestral state among all bacteria, while it also revealed multiple gains and losses of the *DUF4131* and *Lactamase_B* domains (Supplementary Figure S1). For example, in the Deinococcus-Thermus branch, the *Lactamase_B* domain appeared to be gained in the family Thermaceae but remained absent in the Deinococcaceae (Supplementary Figure S2A). In Spirochaetes, the ***C*** profile dominated all major subclades except for the *Brachyspira* genus and *Treponema azotonutricium* ZAS-9, where a gain of the *DUF4131* domain was observed (Supplementary Figure S2B). In Bacteroidetes, the *DUF4131* domain was potentially gained in its common ancestor and retained among the majority of its subclades except for the family Porphyromonadaceae, which is dominated by the ***C*** profile. Additionally, the Bacteroidetes also included a subclade that demonstrated a loss of entire CDCPs. This subclade is composed solely of the endosymbionts of the *Blattabacterium* genus (Supplementary Figure S2C). In Proteobacteria (Supplementary Figures S2D–G), the *DUF4131* domain was observed in the common ancestor of this phylum, followed by a secondary acquisition of the *Lactamase_B* domain in the *Burkholderia* genus and the Neisseriales order of Betaproteobacteria, the Desulfuromonadales order of Deltaproteobacteria, and the Gammaproteobacteria. Several subsequent losses were also observed throughout the different classes of Proteobacteria. Specifically, entire CDCPs were lost in the endosymbionts *Kinetoplastibacterium*, *Buchnera*, and *Wigglesworthia*, as well as in most of the intracellular pathogens of the *Rickettsia*, *Francisella*, and *Coxiella* genera.

The Firmicutes phylum had both the *DUF4131* and the *Lactamase_B* domains in its ancestral state, while it experienced multiple losses and gains of either or both domains in some subclades (Supplementary Figure S2H). For example, both domains were lost in the families Clostridiaceae and Planococcaceae, the *DUF4131* domain was lost in substrains of the genus *Lactobacillus*, and the *Lactamase_B* domain was lost in substrains of the genus *Listeria*, *Desulfosporosinus*, and *Desulfitobacterium*. While the family Staphylococcaceae may have originated with a loss of both domains, the *Lactamase_B* domain was regained in some species of the *Staphylococcus* genus. Similarly, the family Leuconostocaceae also had an initial loss of both domains, while the *DUF4131* domain was regained in the *Leuconostoc* genus.

Subclades of the Tenericutes phylum had an intermingled branching with the Erysipelotrichaceae family of Firmicutes, which is an important species in the human gut microbiota (Kaakoush, 2015) and has emerged between Acholeplasmataceae and other families of Tenericutes (Supplementary Figure S2H). While entire CDCPs were lost in Erysipelotrichaceae, Acholeplasmataceae, and the genus *Mycoplasma*, the rest of this clade was dominated by CDCPs of the ***C*** profile.

### Evolution of Individual Domains in CDCPs

Phylogenies of the *Lactamase_B*, *DUF4131, and Competence* domains (Supplementary Figures S3–S5) provided further insights into the multiple domain gains and losses throughout the species phylogeny. For example, the *Thermodesulfobacterium* genus, while shown to form a clade within Deltaproteobacteria in the species phylogeny (Supplementary Figure S2), clustered with the *Staphylococcus* genus in the *Lactamase_B* domain phylogeny (Supplementary Figure S3). Further examination of the neighboring branches of this clade in the *Lactamase_B* phylogeny revealed a similarity to the Clostridia in Firmicutes, suggesting an acquisition of the *Lactamase_B* domain from Clostridia into both *Staphylococcus* and *Thermodesulfobacterium*. Similarly, the *DUF4131* domain of *Leuconostoc* is a close neighbor of the *Brachyspira* genus in Spirochaetes, and the broader context of the domain phylogeny suggested a potential acquisition from Clostridia to *Leuconostoc* and *Brachyspira* (Supplementary Figure S4).

An overview of the correlation between species distances and domain distances is summarized in Figure 3. Pairs of bacterial strains were divided into subsets based on the CDCP domain profiles encoded in their proteomes. Domain distances were considered among the *Competence* (Figures 3A–D), *DUF4131* (Figures 3E,F), and *Lactamase_B* (Figures 3G,H) phylogeny, and a linear regression was applied to each subplot in order to establish the quantitative association of domain distances as compared to the species distances. For the *Competence* domain, the slope of the linear models ranged from 0.61 to 0.91 for the ***C***, ***DC***, ***DCL***, and ***CL*** profiles, with the ***C*** profile carrying the lowest slope while the ***CL*** profile carrying the highest slope. For the *DUF4131* domain, only two profiles (***DC*** and ***DCL***) were available, and the slope of their linear models are 0.75 and 0.67, respectively. Finally, for the *Lactamase_B* domain, slopes of 0.93 and 0.80, respectively, were observed for the ***CL*** and ***DCL*** profiles. All domain distances had a positive offset as compared to the species distances. The offset ranges from 0.16 to 0.25, with only an exception in the ***DC*** profile of the *Competence* domain, which had an offset of 0.096 (Figure 3B). Overall, linear regression revealed a positive correlation between distances of the species phylogeny and distances of the domain phylogenies. While three profiles of the *Competence* domain (Figures 3B–D) and all profiles of the *Lactamase_B* domain (Figures 3G,H) had a near 1:1 ratio with the species distances, the ***C*** profile of *Competence* (Figure 3A) and all profiles of the *DUF4131* domain (Figures 3E,F) had a lower ratio when compared to the species distances.

Despite capturing the overall trends of species and domain distance correlations, the linear models were insufficient in explaining all variations in the domain distances (R-squared values between 0.38 and 0.69). Some of the variations indicated the lateral acquisition of particular domains. For example, in the above-mentioned case studies, the acquisition of *DUF4131* domain in *Brachyspira* and *Leuconostoc*, and the acquisition of *Lactamase_B* domain in *Staphylococcus* and Thermodesulfobacterium resulted in smaller domain distances among taxa that are more distant in the species phylogeny. Hence, coordinates of these pairs were below the linear regression lines (Figures 3E,H).

Additional variations were identified through a close examination of outliers in domain distances. The termite gut bacterium, *T. azotonutricium* ZAS-9 (Graber et al., 2004), is related to the *Brachyspira* of Spirochaetia while distant from the *Neorickettsia and Wolbachia* of Alphaproteobacteria in the species phylogeny. However, in the *DUF4131* domain phylogeny, *T. azotonutricium* ZAS-9 had a shorter distance to *Neorickettsia* and *Wolbachia*, while it had a longer distance to the *Brachyspira* (Figure 3E). Similarly, the Thermodesulfobacterium and the *Sandaracinus amylolyticus* of Deltaproteobacteria are closely related in the species phylogeny, but they are distant in the *Lactamase_B* domain phylogeny (Figure 3G). Finally, *Acinetobacter baumannii* and *Acinetobacter equi* of Gammaproteobacteria are distant from *Bacillus pumilus* of Firmicutes in the species phylogeny, but the *Lactamase_B* domain distances between *Acinetobacter* and *B. pumilus* are shorter than expected from their species distances. In contrast, the *Lactamase_B* domains of *Acinetobacter* and *Alcanivorax* are distant, despite the taxa being close neighbors in the species phylogeny (Figure 3H).

## Discussion

Natural transformation is a mechanism of horizontal gene transfer and has fundamental roles in bacterial genome evolution (Ambur et al., 2016). The ComEC protein mediates the critical step of ssDNA membrane translocation, and as such it serves as an essential component of the NT machinery. Despite extensive studies of ComEC among selected strains that are known to be naturally competent, little is known about the taxonomic distribution and domain evolution of ComEC in diverse lineages of Bacteria. The current study has provided a stepping stone into bridging this knowledge gap through a global survey of putative ComEC proteins (referred to as CDCPs) and a reconstruction of their ancestral states. Similar analyses can be performed on other proteins that compose the NT machinery to enhance our understanding of its diversity and evolution.

A global presence of CDCPs has been identified among 89% of proteomes and 96% of genomes analyzed in this study. This is surprising considering that only less than 1% of these bacterial strains are known to be naturally competent under laboratory settings (Johnsborg et al., 2007; Johnston et al., 2014). Several considerations could help reconcile the apparent discrepancy between the abundance of CDCPs and the lack of detectable natural competence in many bacteria: (1) the NT process is mediated by many proteins besides ComEC, so variations in other proteins could inhibit the competence phenotype; (2) the NT process is subject to complex regulatory mechanisms, which may prevent the expression of essential proteins in the NT machinery (including ComEC) even when all the proteins are encoded in the genome (Sinha and Redfield, 2012); and (3) besides being a competence protein, ComEC could have other functions. For example, it may be involved in the virulence of *Listeria monocytogenes* (Rabinovich et al., 2012) and *A. baumannii* (Wilharm et al., 2013), and it may contribute to the twitching motility in *Thermus thermophilus* (Salzer et al., 2016).

For all but one strain, only a single CDCP has been found in each proteome (Supplementary Table S2). The two closely related copies of CDCPs in *P. sulfonivorans* Ar51 are likely to be a result of a recent gene duplication event, and the fact that one protein appears to be a truncated copy of the other suggests the former is under the process of being degenerated from the genome. The broad presence of single copy CDCP-encoding genes suggests its key roles in the cell machinery, although its function and regulation in strains that are not known to undergo NT is still unclear.

Despite the near universal presence, CDCPs are missing from 231 bacterial strains using search criteria defined in this study. These strains are enriched with obligate endosymbionts and intracellular pathogens. Considering the significant genomic reductions in these organisms due to genetic drift (Wernegreen and Moran, 1999; Moran, 2002; Kuo et al., 2009), ComEC proteins may have been lost from these strains, either in the ancestral state of an entire genera (e.g., *Buchnera*) or among selected subgroups (e.g., *Mycoplasma*, Supplementary Figure S2).

Two domains, *DUF4131* and *Lactamase_B*, are commonly present in CDCPs. While five additional domains have also been observed, these domains occur in low frequencies and hence could have minor roles in the evolution of CDCPs. Of the dominant domain profiles, the *Competence* only (***C*)** profile appears to be the most ancient and most widely distributed among bacterial phyla (Figure 2). This observation has been supported with an ancestral state reconstruction (Supplementary Figures S1, S2), which predicted that the protein has emerged from the ***C*** profile, and subsequently modified through multiple gains and losses of the *DUF4131* and *Lactamase_B* domains over diverse lineages.

An earlier study of naturally competent strains and the ComEC proteins encoded by these strains suggests the *Competence* domain evolve at a rate sixfold higher than the corresponding species (Johnston et al., 2014). This phenomenon, however, was not observed in our broader survey of putative ComEC proteins. In contrast, the *Competence* domain of ***DC***, ***DCL***, and ***CL*** proteins appeared to maintain a near 1:1 slope, and that of the ***C*** profile had a lower slope of 0.61 (Figures 3A–D). Further, the larger dataset provided from this study has revealed additional complexity in the evolution of ComEC proteins that are not explained by the linear models. This is reflected in the relatively low R-squared values in linear fitting. As detailed in several case studies in the Results section, this deviation from linear models could indicate potential events of horizontal domain acquisition that contributes to the gains and losses of *DUF4131* and *Lactamase_B* in CDCPs among diverse lineages. Interestingly, no obvious HGT has been found for the *DUF4131* domain among proteins that carry the ***DCL*** profile (Figure 3F). Since the acquisition of *DUF4131* generally proceeds the acquisition of *Lactamase_B* (Supplementary Figure S1), it suggests that proteins of the ***DCL*** profile are unlikely to lose the *DUF4131* domain and then regain it into the resulting ***CL*** proteins.

## Author Contributions

YZ conceived the project and led the data analysis and interpretation. ZP contributed to data acquisition, analysis and interpretation. All authors composed the manuscript.

## Conflict of Interest Statement

The authors declare that the research was conducted in the absence of any commercial or financial relationships that could be construed as a potential conflict of interest.

## Supplementary Material

FIGURE S1 Ancestral reconstruction of CDCPs in the species phylogeny. Five states were considered in the reconstruction: No CDCP (***NC***), *Competence* only (***C***), *DUF4131* and *Competence* (***DC***), *Competence* and *Lactamase_B* (***CL***), and *DUF4131*, *Competence*, and *Lactamase_B* (***DCL***). Phyla (except for the case of the Proteobacteria which were analyzed at the class level) with greater than 20 leaves were labeled. Selected groups that carry the ***NC*** profile were also labeled throughout the phylogeny.

FIGURE S2 Detailed view of the sub-branches displayed in Supplementary Figure S1 along with the ancestral state reconstruction results. The Deinococcus-Thermus **(A)**, Spirochaetes **(B)**, Bacteroidetes **(C)**, Betaproteobacteria **(D)**, Deltaproteobacteria **(E)**, Gammaproteobacteria **(F)**, Alphaproteobacteria **(G)**, and Firmicutes **(H)** are displayed.

FIGURE S3 Phylogenetic reconstruction of the *Lactamase_B* domain.

FIGURE S4 Phylogenetic reconstruction of the *DUF4131* domain.

FIGURE S5 Phylogenetic reconstruction of the *Competence* domain.

TABLE S1 Domain profiles of ComEC in bacteria confirmed to be capable of natural transformation.

TABLE S2 Taxonomic and domain presence/absence data for CDCPs predicted from 5,574 complete bacterial proteomes/genomes. A total of eight domains (including *Competence*) were observed among all of the identified CDCPs. The abundance of each domain was recorded in their corresponding columns, with zero indicating the absence of a domain in a CDCP, and non-zero values indicating the number of occurrences of a domain in a protein. Each row of the table represents a unique combination of a CDCP (if identified) and a bacterial strain that carries the protein. The columns included the protein accession number (if present), protein length, genome assembly identifier from the NCBI RefSeq database (O’Leary et al., 2016), and the taxonomy assignments from the NCBI Taxonomy databases (Sayers et al., 2009). For the strains in which no CDCP was identified, the domain profiles were filled with all zeros for each domain. In the one case where two CDCPs were identified in one strain (NCBI assembly GCF_001484605.1), two entries/rows were included with the same NCBI assembly identifier mapped to the two different CDCP protein identifiers.

DATA SHEET S1 Manual operations for the visualization of the species phylogeny.
