# Supplementary material for: Evolution of the Natural Transformation Protein, ComEC, in Bacteria
Source: Front Microbiol. 2018 Dec 12;9:2980. doi: 10.3389/fmicb.2018.02980 (PMC6299819; doi:10.3389/fmicb.2018.02980)

Supplementary Figure S2A

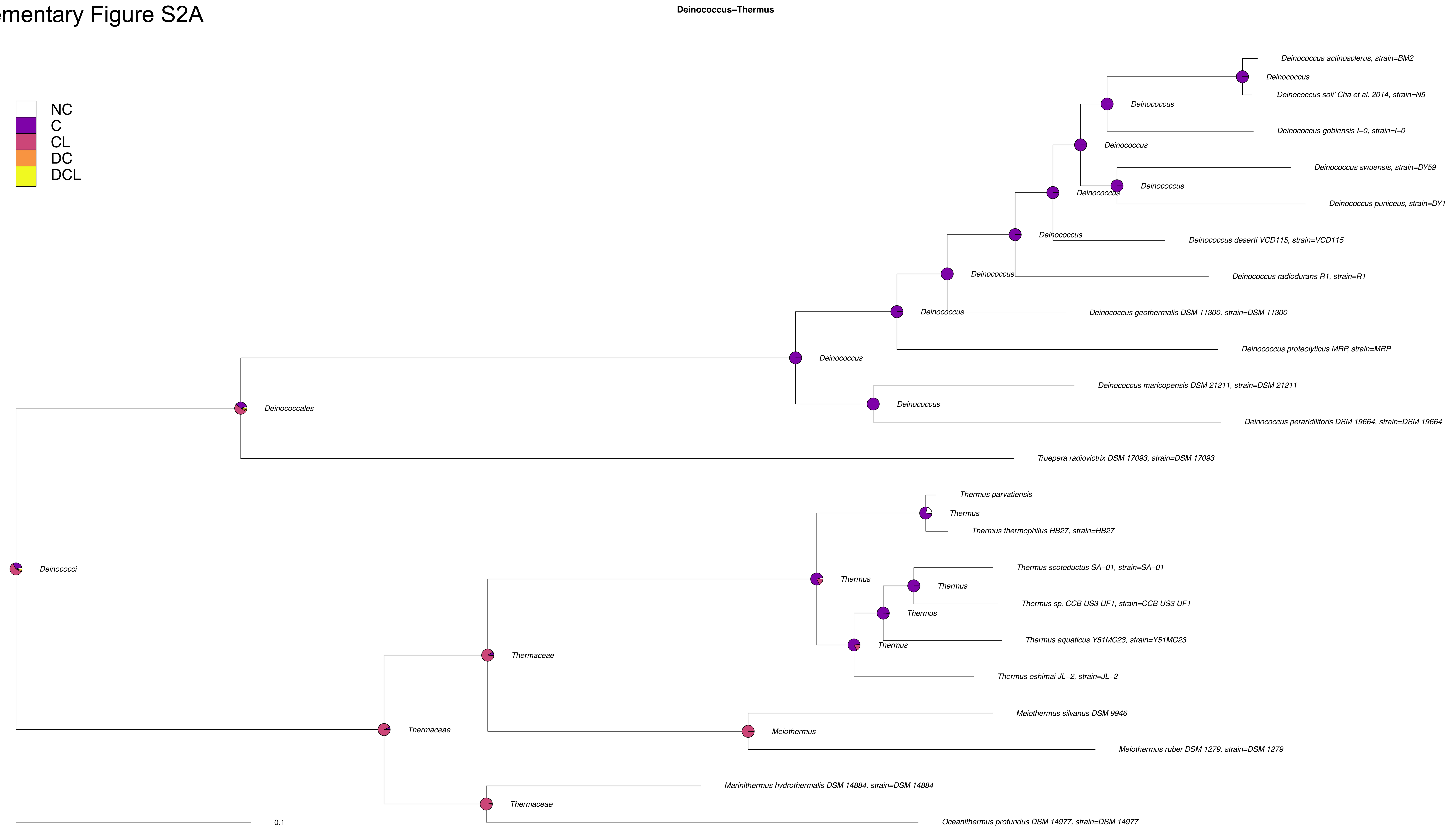

Supplementary Figure S2B

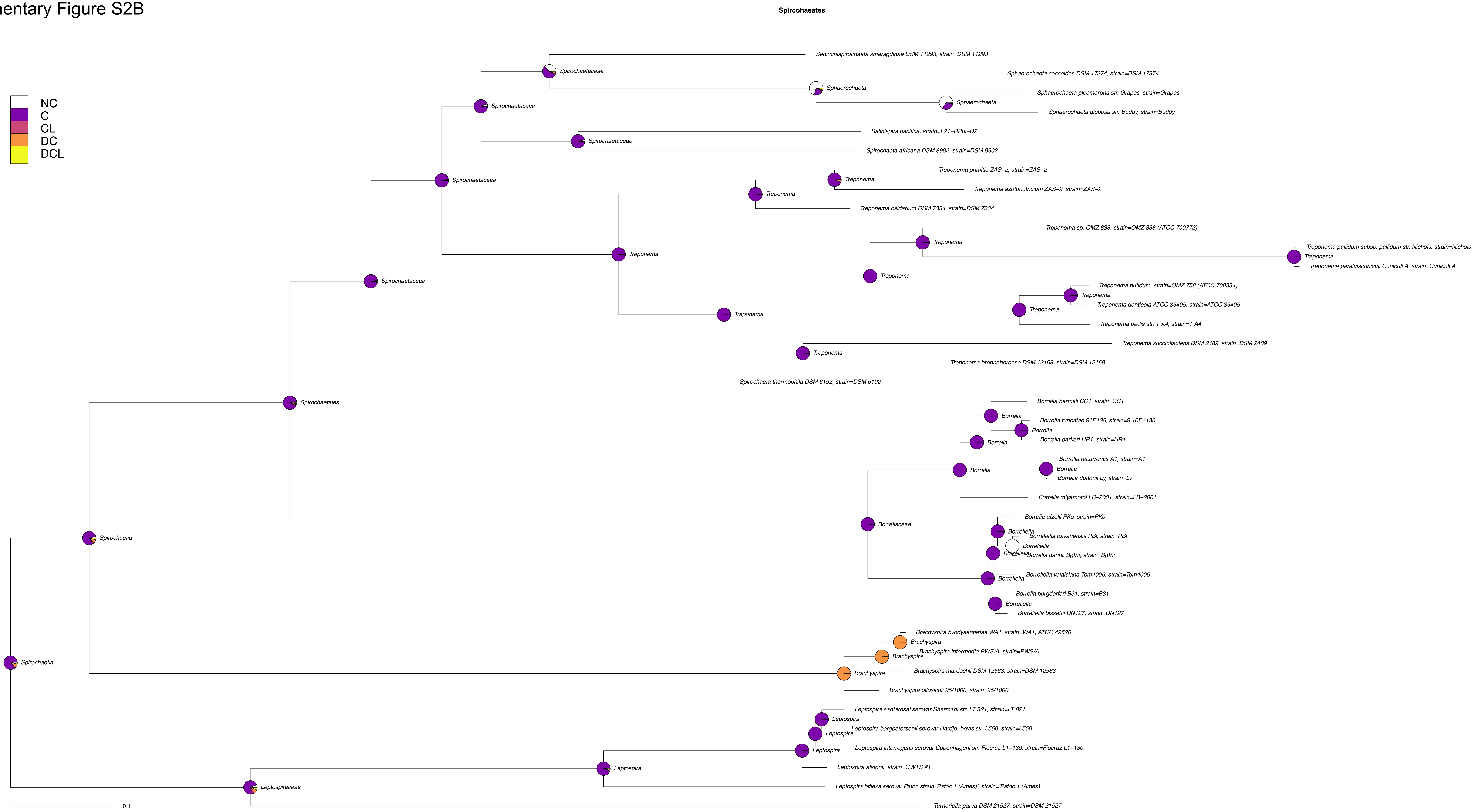

Supplementary Figure S2C

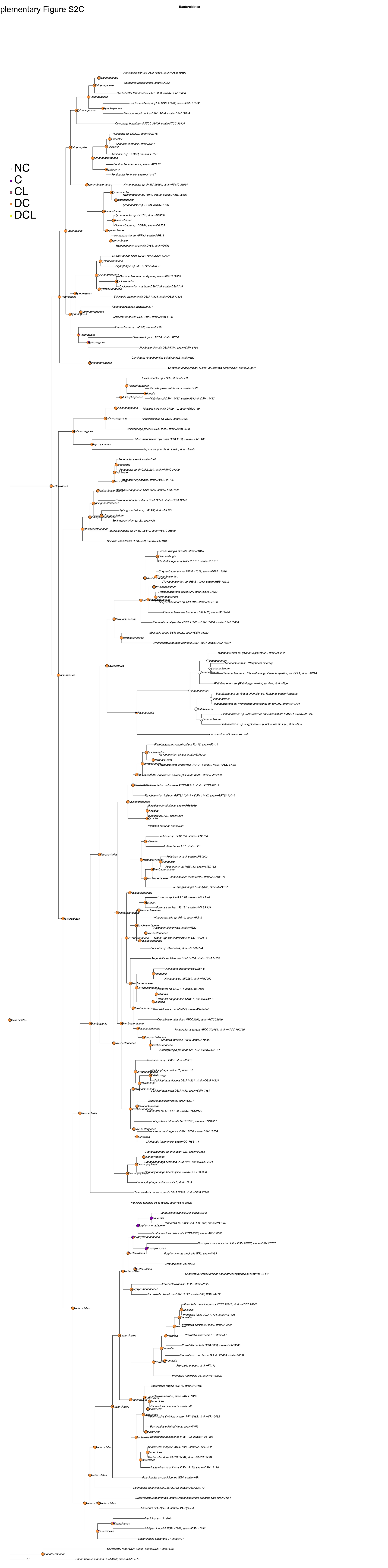

Supplementary Figure S2D

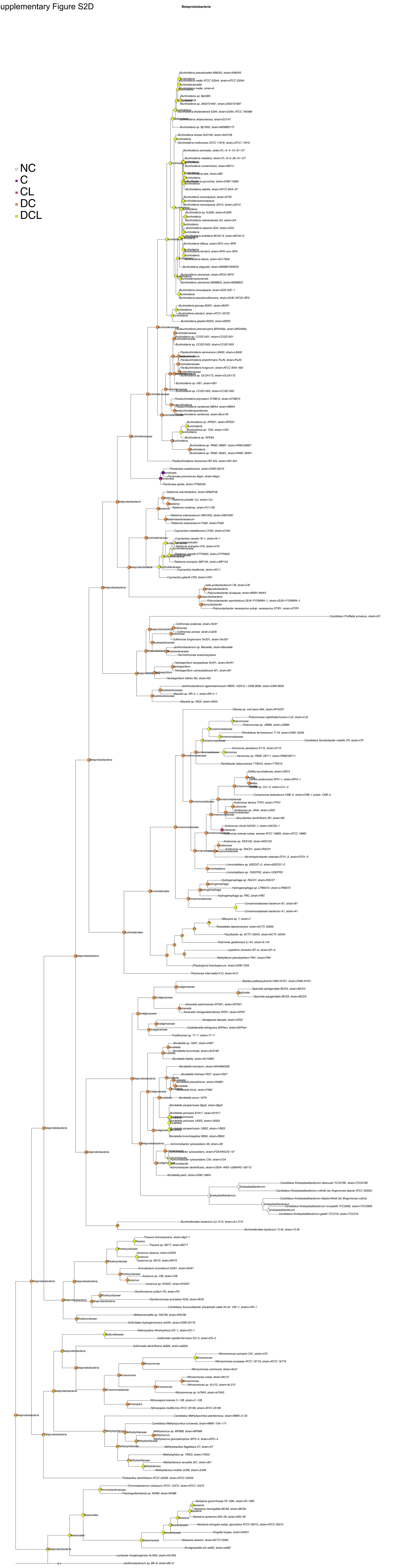

Supplementary Figure S2E

Deltaproteobacteria

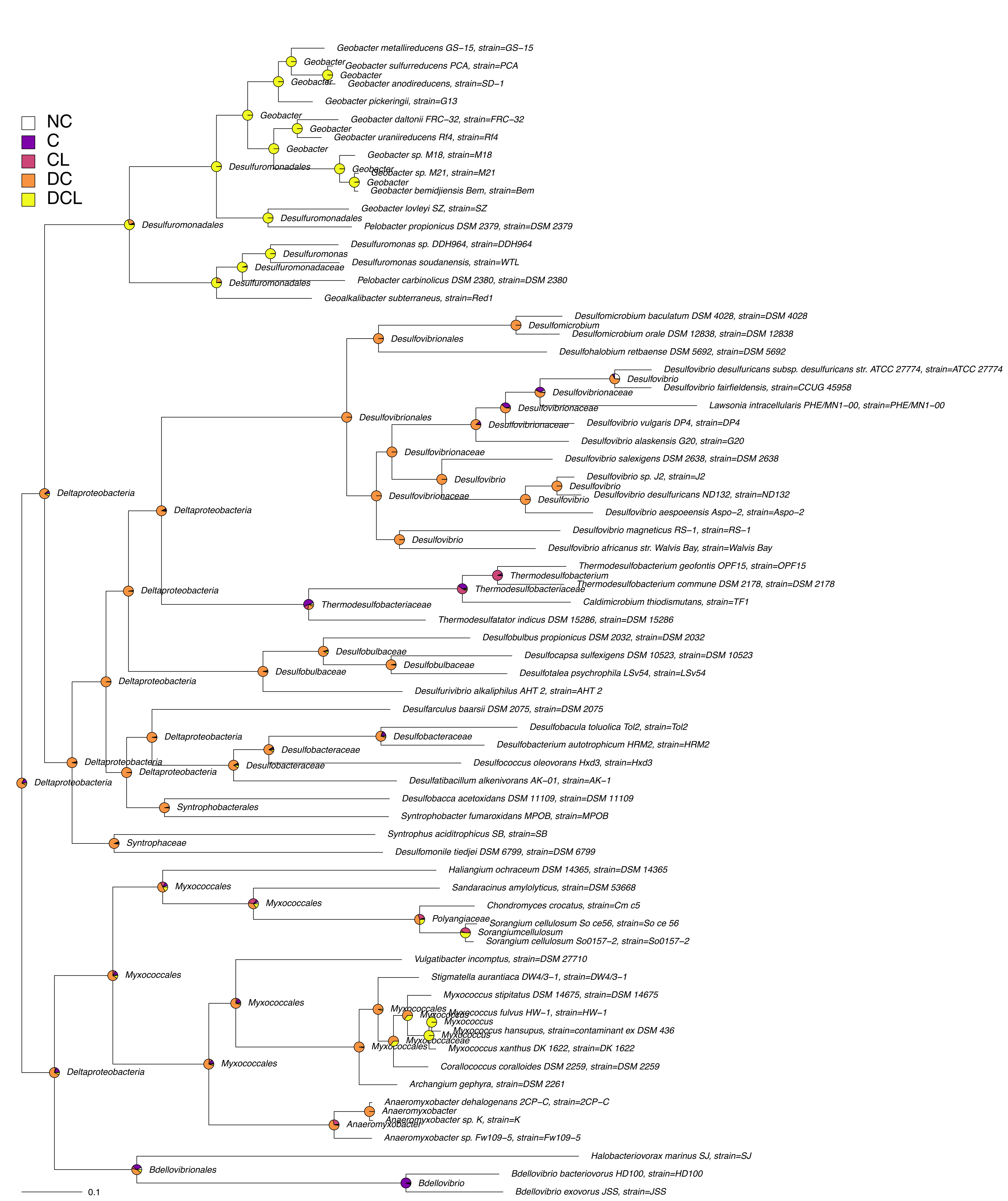

Supplementary Figure S2F

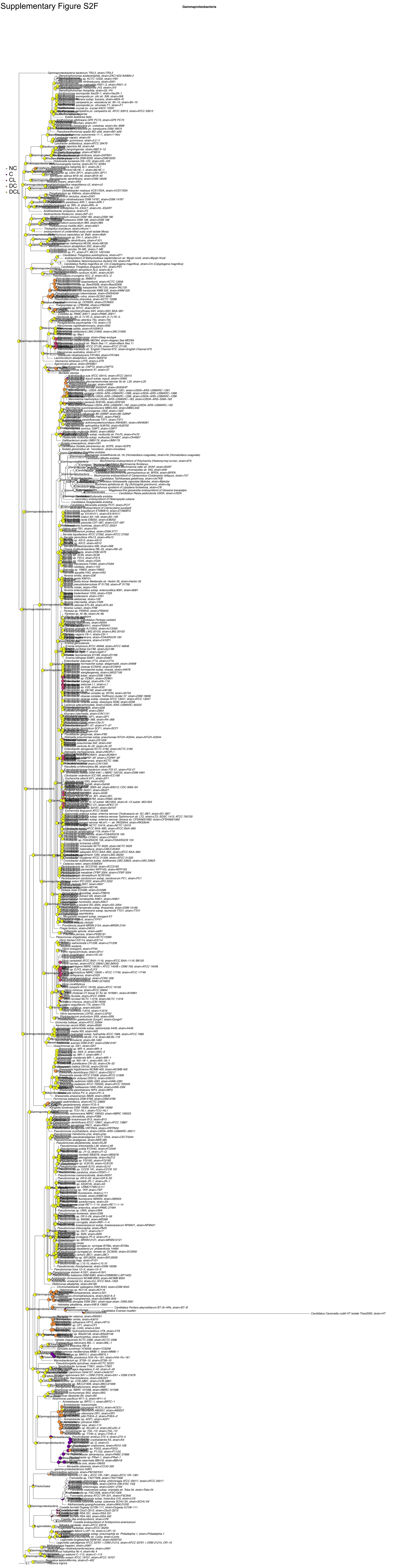

Supplementary Figure S2G

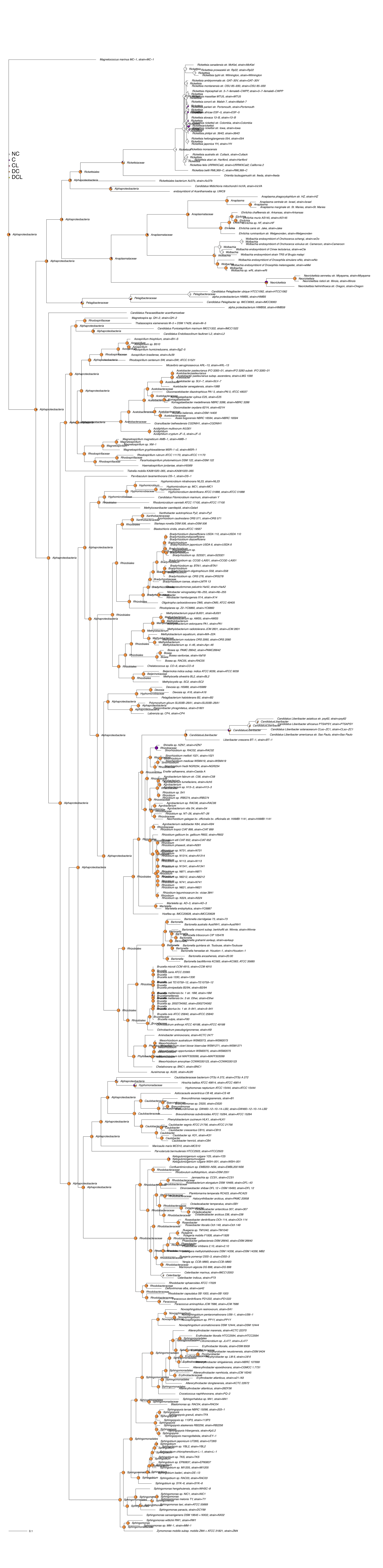

Supplementary Figure S2H

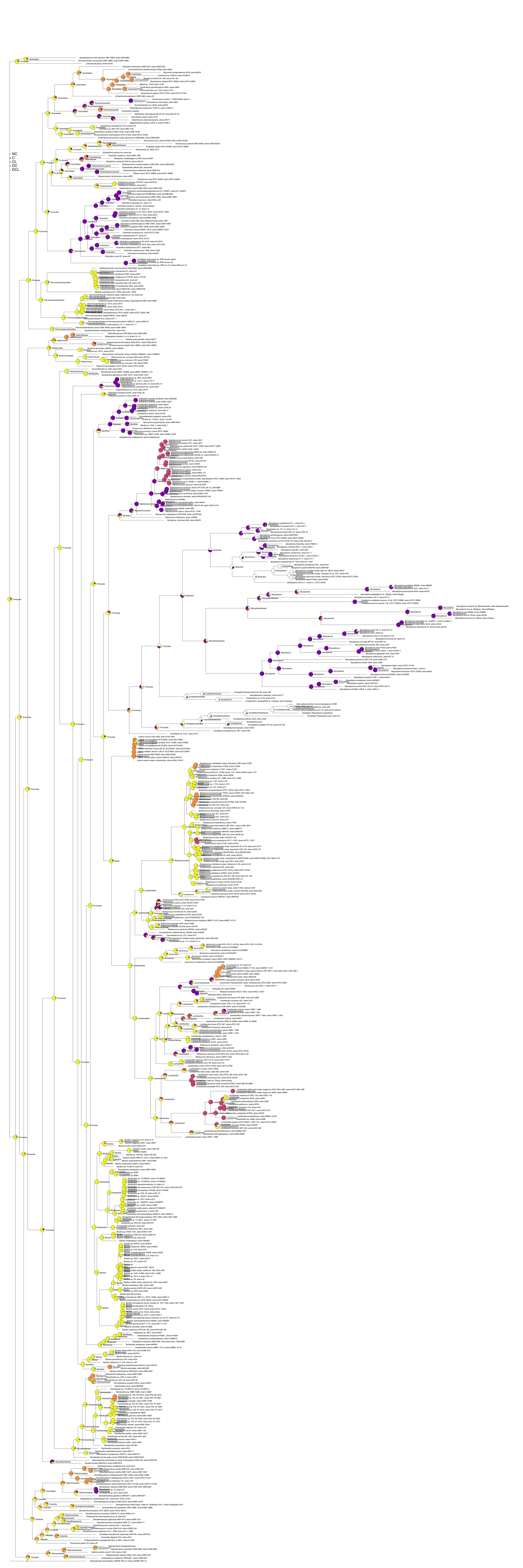

Supplement: FIGURE S2 — Detailed view of the sub-branches displayed in Supplementary Figure S1 along with the ancestral state reconstruction results. The Deinococcus-Thermus (A), Spirochaetes (B), Bacteroidetes (C), Betaproteobacteria (D), Deltaproteobacteria (E), Gammaproteobacteria (F), Alphaproteobacteria (G), and Firmicutes (H) are displayed. [file Image_2.pdf]
