# Supplementary material for: Evolution of the Natural Transformation Protein, ComEC, in Bacteria
Source: Front Microbiol. 2018 Dec 12;9:2980. doi: 10.3389/fmicb.2018.02980 (PMC6299819; doi:10.3389/fmicb.2018.02980)
Supplement: FIGURE S3 — Phylogenetic reconstruction of the Lactamase_B domain. [file Image_3.pdf]

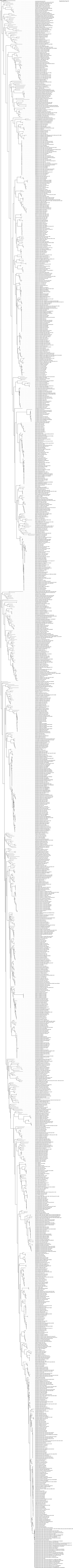

Supplementary Figure S3
