# Supplementary material for: Evolution of the Natural Transformation Protein, ComEC, in Bacteria
Source: Front Microbiol. 2018 Dec 12;9:2980. doi: 10.3389/fmicb.2018.02980 (PMC6299819; doi:10.3389/fmicb.2018.02980)
Supplement: FIGURE S5 — Phylogenetic reconstruction of the Competence domain. [file Image_5.pdf]

Supplementary Figure S5

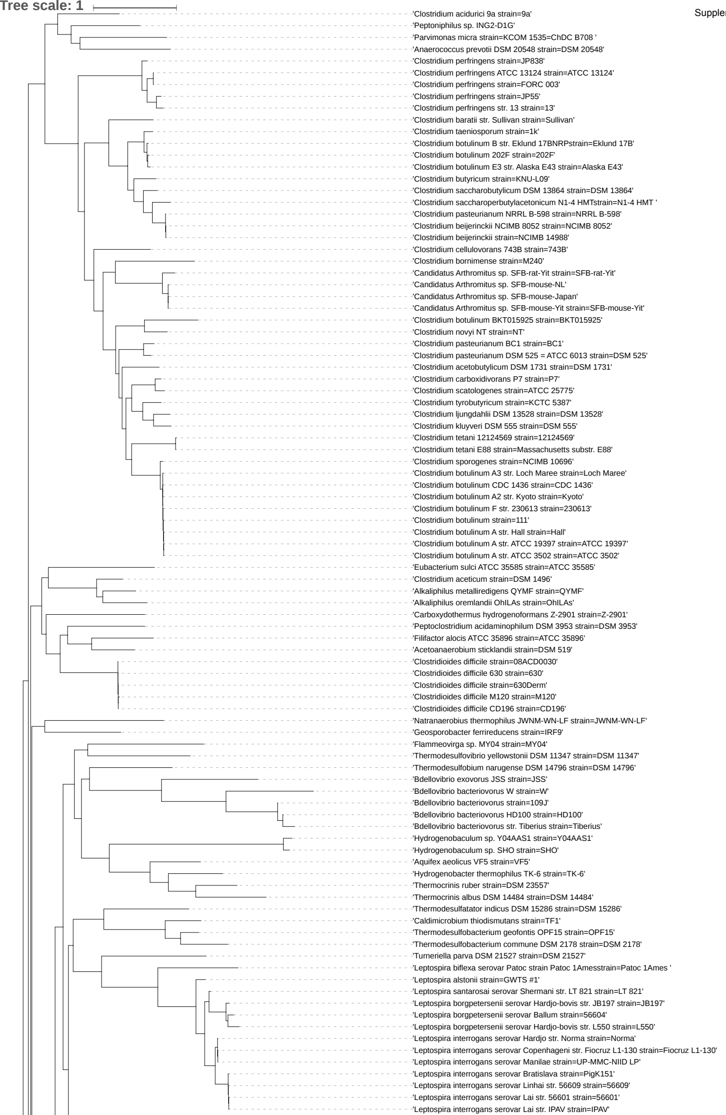

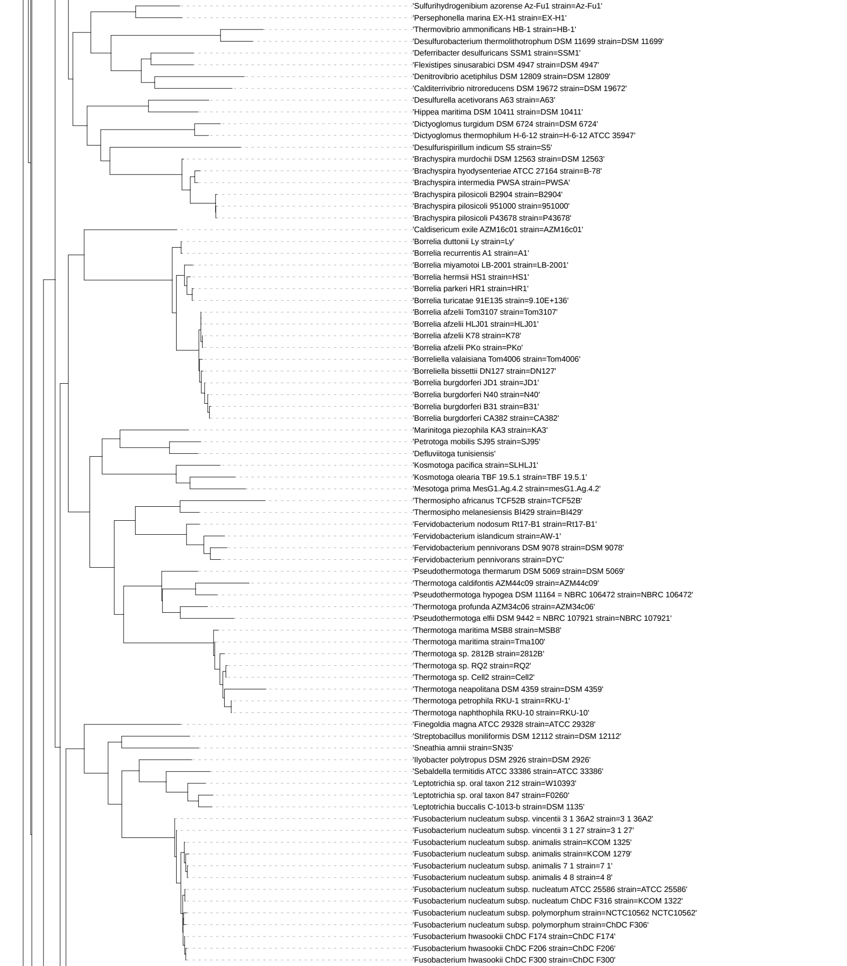
'Sulfurihydrogenibium azorense Az-Fu1 strain=Az-Fu1'
'Persephonella marina EX-H1 strain=EX-H1'
'Thermovibrio ammonificans HB-1 strain=HB-1'
'Desulfurobacterium thermolithotrophum DSM 11699 strain=DSM 11699'
'Deferribacter desulfuricans SSM1 strain=SSM1'
'Flexistipes sinusarabici DSM 4947 strain=DSM 4947'
'Denitrovibrio acetiphilus DSM 12809 strain=DSM 12809'
'Calditerrivibrio nitroreducens DSM 19672 strain=DSM 19672'
'Desulfurella acetivorans A63 strain=A63'
'Hippea maritima DSM 10411 strain=DSM 10411'
'Dictyoglomus turgidum DSM 6724 strain=DSM 6724'
'Dictyoglomus thermophilum H-6-12 strain=H-6-12 ATCC 35947'
'Desulfurispirillum indicum S5 strain=S5'
'Brachyspira murdochii DSM 12563 strain=DSM 12563'
'Brachyspira hyodysenteriae ATCC 27164 strain=B-78'
'Brachyspira intermedia PWSA strain=PWSA'
'Brachyspira pilosicoli B2904 strain=B2904'
'Brachyspira pilosicoli 951000 strain=951000'
'Brachyspira pilosicoli P43678 strain=P43678'
'Caldisericum exile AZM16c01 strain=AZM16c01'
'Borrelia duttonii Ly strain=Ly'
'Borrelia recurrentis A1 strain=A1'
'Borrelia miyamotoi LB-2001 strain=LB-2001'
'Borrelia hermsii HS1 strain=HS1'
'Borrelia parkeri HR1 strain=HR1'
'Borrelia turicatae 91E135 strain=9.10E+136'
'Borrelia afzelii Tom3107 strain=Tom3107'
'Borrelia afzelii HLJ01 strain=HLJ01'
'Borrelia afzelii K78 strain=K78'
'Borrelia afzelii PKo strain=PKo'
'Borreliella valaisiana Tom4006 strain=Tom4006'
'Borreliella bissettii DN127 strain=DN127'
'Borrelia burgdorferi JD1 strain=JD1'
'Borrelia burgdorferi N40 strain=N40'
'Borrelia burgdorferi B31 strain=B31'
'Borrelia burgdorferi CA382 strain=CA382'
'Marinitoga piezophila KA3 strain=KA3'
'Petrotoga mobilis SJ95 strain=SJ95'
'Defluviitoga tunisiensis'
'Kosmotoga pacifica strain=SLHLJ1'
'Kosmotoga olearia TBF 19.5.1 strain=TBF 19.5.1'
'Mesotoga prima MesG1.Ag.4.2 strain=mesG1.Ag.4.2'
'Thermosipho africanus TCF52B strain=TCF52B'
'Thermosipho melanesiensis BI429 strain=BI429'
'Fervidobacterium nodosum Rt17-B1 strain=Rt17-B1'
'Fervidobacterium islandicum strain=AW-1'
'Fervidobacterium pennivorans DSM 9078 strain=DSM 9078'
'Fervidobacterium pennivorans strain=DYC'
'Pseudothermotoga thermarum DSM 5069 strain=DSM 5069'
'Thermotoga caldifontis AZM44c09 strain=AZM44c09'
'Pseudothermotoga hypogea DSM 11164 = NBRC 106472 strain=NBRC 106472'
'Thermotoga profunda AZM34c06 strain=AZM34c06'
'Pseudothermotoga elfii DSM 9442 = NBRC 107921 strain=NBRC 107921'
'Thermotoga maritima MSB8 strain=MSB8'
'Thermotoga maritima strain=Tma100'
'Thermotoga sp. 2812B strain=2812B'
'Thermotoga sp. RQ2 strain=RQ2'
'Thermotoga sp. Cell2 strain=Cell2'
'Thermotoga neapolitana DSM 4359 strain=DSM 4359'
'Thermotoga petrophila RKU-1 strain=RKU-1'
'Thermotoga naphthophila RKU-10 strain=RKU-10'
'Finegoldia magna ATCC 29328 strain=ATCC 29328'
'Streptobacillus moniliformis DSM 12112 strain=DSM 12112'
'Sneathia amnii strain=SN35'
'Ilyobacter polytropus DSM 2926 strain=DSM 2926'
'Sebaldella termitidis ATCC 33386 strain=ATCC 33386'
'Leptotrichia sp. oral taxon 212 strain=W10393'
'Leptotrichia sp. oral taxon 847 strain=F0260'
'Leptotrichia buccalis C-1013-b strain=DSM 1135'
'Fusobacterium nucleatum subsp. vincentii 3 1 36A2 strain=3 1 36A2'
'Fusobacterium nucleatum subsp. vincentii 3 1 27 strain=3 1 27'
'Fusobacterium nucleatum subsp. animalis strain=KCOM 1325'
'Fusobacterium nucleatum subsp. animalis strain=KCOM 1279'
'Fusobacterium nucleatum subsp. animalis 7 1 strain=7 1'
'Fusobacterium nucleatum subsp. animalis 4 8 strain=4 8'
'Fusobacterium nucleatum subsp. nucleatum ATCC 25586 strain=ATCC 25586'
'Fusobacterium nucleatum subsp. nucleatum ChDC F316 strain=KCOM 1322'
'Fusobacterium nucleatum subsp. polymorphum strain=NCTC10562 NCTC10562'
'Fusobacterium nucleatum subsp. polymorphum strain=ChDC F306'
'Fusobacterium hwasookii ChDC F174 strain=ChDC F174'
'Fusobacterium hwasookii ChDC F206 strain=ChDC F206'
'Fusobacterium hwasookii ChDC F300 strain=ChDC F300'

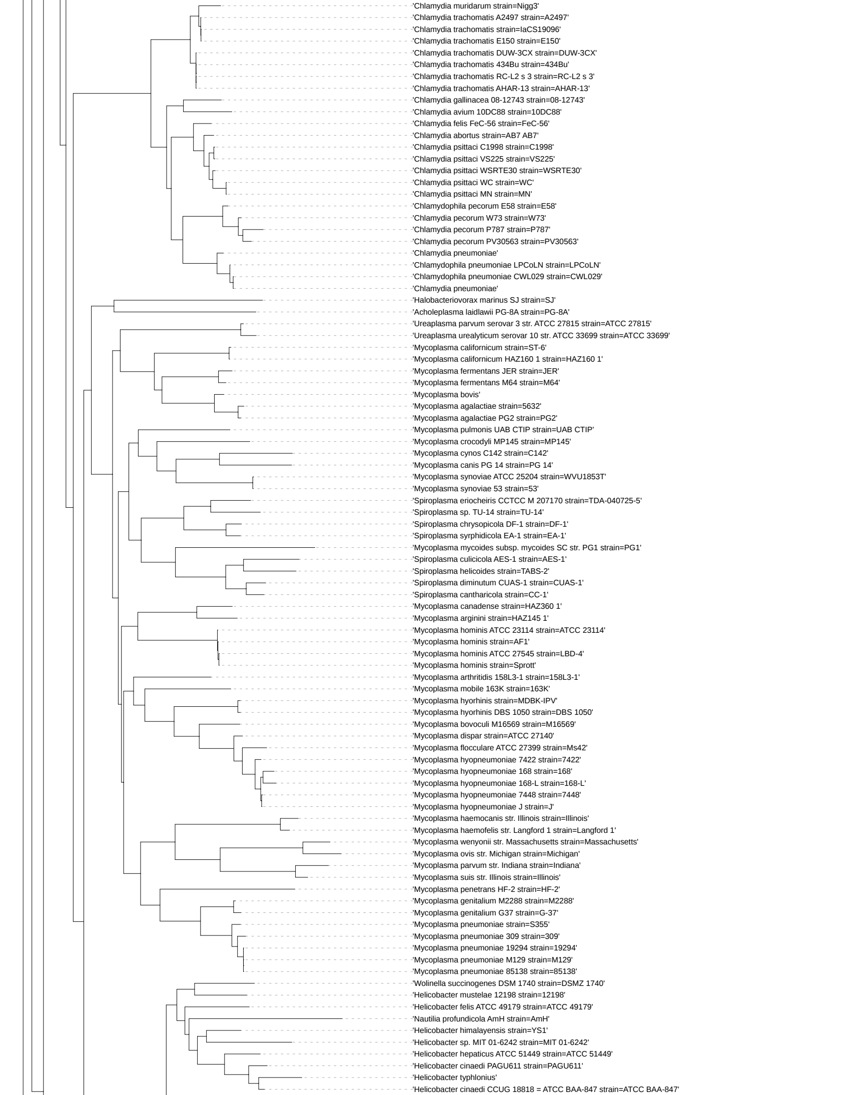
'Chlamydia muridarum strain=Nigg3'
'Chlamydia trachomatis A2497 strain=A2497'
'Chlamydia trachomatis strain=IaCS19096'
'Chlamydia trachomatis E150 strain=E150'
'Chlamydia trachomatis DUW-3CX strain=DUW-3CX'
'Chlamydia trachomatis 434Bu strain=434Bu'
'Chlamydia trachomatis RC-L2 s 3 strain=RC-L2 s 3'
'Chlamydia trachomatis AHAR-13 strain=AHAR-13'
'Chlamydia gallinacea 08-12743 strain=08-12743'
'Chlamydia avium 10DC88 strain=10DC88'
'Chlamydia felis FeC-56 strain=FeC-56'
'Chlamydia abortus strain=AB7 AB7'
'Chlamydia psittaci C1998 strain=C1998'
'Chlamydia psittaci VS225 strain=VS225'
'Chlamydia psittaci WSRTE30 strain=WSRTE30'
'Chlamydia psittaci WC strain=WC'
'Chlamydia psittaci MN strain=MN'
'Chlamydophila pecorum E58 strain=E58'
'Chlamydia pecorum W73 strain=W73'
'Chlamydia pecorum P787 strain=P787'
'Chlamydia pecorum PV30563 strain=PV30563'
'Chlamydia pneumoniae'
'Chlamydophila pneumoniae LPCoLN strain=LPCoLN'
'Chlamydophila pneumoniae CWL029 strain=CWL029'
'Chlamydia pneumoniae'
'Halobacteriovorax marinus SJ strain=SJ'
'Acholeplasma laidlawii PG-8A strain=PG-8A'
'Ureaplasma parvum serovar 3 str. ATCC 27815 strain=ATCC 27815'
'Ureaplasma urealyticum serovar 10 str. ATCC 33699 strain=ATCC 33699'
'Mycoplasma californicum strain=ST-6'
'Mycoplasma californicum HAZ160 1 strain=HAZ160 1'
'Mycoplasma fermentans JER strain=JER'
'Mycoplasma fermentans M64 strain=M64'
'Mycoplasma bovis'
'Mycoplasma agalactiae strain=5632'
'Mycoplasma agalactiae PG2 strain=PG2'
'Mycoplasma pulmonis UAB CTIP strain=UAB CTIP'
'Mycoplasma crocodyli MP145 strain=MP145'
'Mycoplasma cynos C142 strain=C142'
'Mycoplasma canis PG 14 strain=PG 14'
'Mycoplasma synoviae ATCC 25204 strain=WVU1853T'
'Mycoplasma synoviae 53 strain=53'
'Spiroplasma eriocheiris CCTCC M 207170 strain=TDA-040725-5'
'Spiroplasma sp. TU-14 strain=TU-14'
'Spiroplasma chrysopicola DF-1 strain=DF-1'
'Spiroplasma syrphidicola EA-1 strain=EA-1'
'Mycoplasma mycoides subsp. mycoides SC str. PG1 strain=PG1'
'Spiroplasma culicicola AES-1 strain=AES-1'
'Spiroplasma helicoides strain=TABS-2'
'Spiroplasma diminutum CUAS-1 strain=CUAS-1'
'Spiroplasma cantharicola strain=CC-1'
'Mycoplasma canadense strain=HAZ360 1'
'Mycoplasma arginini strain=HAZ145 1'
'Mycoplasma hominis ATCC 23114 strain=ATCC 23114'
'Mycoplasma hominis strain=AF1'
'Mycoplasma hominis ATCC 27545 strain=LBD-4'
'Mycoplasma hominis strain=Sprott'
'Mycoplasma arthritidis 158L3-1 strain=158L3-1'
'Mycoplasma mobile 163K strain=163K'
'Mycoplasma hyorhinis strain=MDBK-IPV'
'Mycoplasma hyorhinis DBS 1050 strain=DBS 1050'
'Mycoplasma bovoculi M16569 strain=M16569'
'Mycoplasma dispar strain=ATCC 27140'
'Mycoplasma flocculare ATCC 27399 strain=Ms42'
'Mycoplasma hyopneumoniae 7422 strain=7422'
'Mycoplasma hyopneumoniae 168 strain=168'
'Mycoplasma hyopneumoniae 168-L strain=168-L'
'Mycoplasma hyopneumoniae 7448 strain=7448'
'Mycoplasma hyopneumoniae J strain=J'
'Mycoplasma haemocanis str. Illinois strain=Illinois'
'Mycoplasma haemofelis str. Langford 1 strain=Langford 1'
'Mycoplasma wenyonii str. Massachusetts strain=Massachusetts'
'Mycoplasma ovis str. Michigan strain=Michigan'
'Mycoplasma parvum str. Indiana strain=Indiana'
'Mycoplasma suis str. Illinois strain=Illinois'
'Mycoplasma penetrans HF-2 strain=HF-2'
'Mycoplasma genitalium M2288 strain=M2288'
'Mycoplasma genitalium G37 strain=G-37'
'Mycoplasma pneumoniae strain=S355'
'Mycoplasma pneumoniae 309 strain=309'
'Mycoplasma pneumoniae 19294 strain=19294'
'Mycoplasma pneumoniae M129 strain=M129'
'Mycoplasma pneumoniae 85138 strain=85138'
'Wolinella succinogenes DSM 1740 strain=DSMZ 1740'
'Helicobacter mustelae 12198 strain=12198'
'Helicobacter felis ATCC 49179 strain=ATCC 49179'
'Nautilia profundicola AmH strain=AmH'
'Helicobacter himalayensis strain=YS1'
'Helicobacter sp. MIT 01-6242 strain=MIT 01-6242'
'Helicobacter hepaticus ATCC 51449 strain=ATCC 51449'
'Helicobacter cinaedi PAGU611 strain=PAGU611'
'Helicobacter typhlonius'
'Helicobacter cinaedi CCUG 18818 = ATCC BAA-847 strain=ATCC BAA-847'

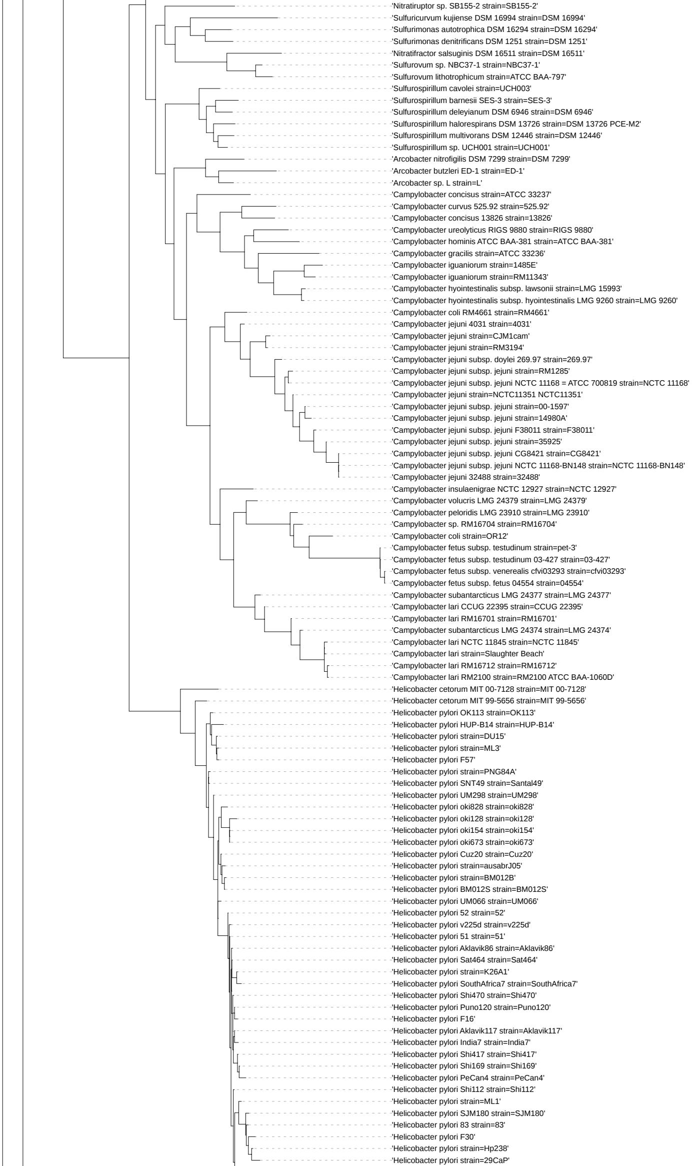

'Nitratiruptor sp. SB155-2 strain=SB155-2'
'Sulfuricurvum kujiense DSM 16994 strain=DSM 16994'
'Sulfurimonas autotrophica DSM 16294 strain=DSM 16294'
'Sulfurimonas denitrificans DSM 1251 strain=DSM 1251'
'Nitratifractor salsuginis DSM 16511 strain=DSM 16511'
'Sulfurovum sp. NBC37-1 strain=NBC37-1'
'Sulfurovum lithotrophicum strain=ATCC BAA-797'
'Sulfurospirillum cavolei strain=UCH003'
'Sulfurospirillum barnesii SES-3 strain=SES-3'
'Sulfurospirillum deleyianum DSM 6946 strain=DSM 6946'
'Sulfurospirillum halorespirans DSM 13726 strain=DSM 13726 PCE-M2'
'Sulfurospirillum multivorans DSM 12446 strain=DSM 12446'
'Sulfurospirillum sp. UCH001 strain=UCH001'
'Arcobacter nitrofigilis DSM 7299 strain=DSM 7299'
'Arcobacter butzleri ED-1 strain=ED-1'
'Arcobacter sp. L strain=L'
'Campylobacter concisus strain=ATCC 33237'
'Campylobacter curvus 525.92 strain=525.92'
'Campylobacter concisus 13826 strain=13826'
'Campylobacter ureolyticus RIGS 9880 strain=RIGS 9880'
'Campylobacter hominis ATCC BAA-381 strain=ATCC BAA-381'
'Campylobacter gracilis strain=ATCC 33236'
'Campylobacter iguaniorum strain=1485E'
'Campylobacter iguaniorum strain=RM11343'
'Campylobacter hyointestinalis subsp. lawsonii strain=LMG 15993'
'Campylobacter hyointestinalis subsp. hyointestinalis LMG 9260 strain=LMG 9260'
'Campylobacter coli RM4661 strain=RM4661'
'Campylobacter jejuni 4031 strain=4031'
'Campylobacter jejuni strain=CJM1cam'
'Campylobacter jejuni strain=RM3194'
'Campylobacter jejuni subsp. doylei 269.97 strain=269.97'
'Campylobacter jejuni subsp. jejuni strain=RM1285'
'Campylobacter jejuni subsp. jejuni NCTC 11168 = ATCC 700819 strain=NCTC 11168'
'Campylobacter jejuni strain=NCTC11351 NCTC11351'
'Campylobacter jejuni subsp. jejuni strain=00-1597'
'Campylobacter jejuni subsp. jejuni strain=14980A'
'Campylobacter jejuni subsp. jejuni F38011 strain=F38011'
'Campylobacter jejuni subsp. jejuni strain=35925'
'Campylobacter jejuni subsp. jejuni CG8421 strain=CG8421'
'Campylobacter jejuni subsp. jejuni NCTC 11168-BN148 strain=NCTC 11168-BN148'
'Campylobacter jejuni 32488 strain=32488'
'Campylobacter insulaenigrae NCTC 12927 strain=NCTC 12927'
'Campylobacter volucris LMG 24379 strain=LMG 24379'
'Campylobacter peloridis LMG 23910 strain=LMG 23910'
'Campylobacter sp. RM16704 strain=RM16704'
'Campylobacter coli strain=OR12'
'Campylobacter fetus subsp. testudinum strain=pet-3'
'Campylobacter fetus subsp. testudinum 03-427 strain=03-427'
'Campylobacter fetus subsp. venerealis cfvi03293 strain=cfvi03293'
'Campylobacter fetus subsp. fetus 04554 strain=04554'
'Campylobacter subantarcticus LMG 24377 strain=LMG 24377'
'Campylobacter lari CCUG 22395 strain=CCUG 22395'
'Campylobacter lari RM16701 strain=RM16701'
'Campylobacter subantarcticus LMG 24374 strain=LMG 24374'
'Campylobacter lari NCTC 11845 strain=NCTC 11845'
'Campylobacter lari strain=Slaughter Beach'
'Campylobacter lari RM16712 strain=RM16712'
'Campylobacter lari RM2100 strain=RM2100 ATCC BAA-1060D'
'Helicobacter cetorum MIT 00-7128 strain=MIT 00-7128'
'Helicobacter cetorum MIT 99-5656 strain=MIT 99-5656'
'Helicobacter pylori OK113 strain=OK113'
'Helicobacter pylori HUP-B14 strain=HUP-B14'
'Helicobacter pylori strain=DU15'
'Helicobacter pylori strain=ML3'
'Helicobacter pylori F57'
'Helicobacter pylori strain=PNG84A'
'Helicobacter pylori SNT49 strain=Santal49'
'Helicobacter pylori UM298 strain=UM298'
'Helicobacter pylori oki828 strain=oki828'
'Helicobacter pylori oki128 strain=oki128'
'Helicobacter pylori oki154 strain=oki154'
'Helicobacter pylori oki673 strain=oki673'
'Helicobacter pylori Cuz20 strain=Cuz20'
'Helicobacter pylori strain=ausabrJ05'
'Helicobacter pylori strain=BM012B'
'Helicobacter pylori BM012S strain=BM012S'
'Helicobacter pylori UM066 strain=UM066'
'Helicobacter pylori 52 strain=52'
'Helicobacter pylori v225d strain=v225d'
'Helicobacter pylori 51 strain=51'
'Helicobacter pylori Aklavik86 strain=Aklavik86'
'Helicobacter pylori Sat464 strain=Sat464'
'Helicobacter pylori strain=K26A1'
'Helicobacter pylori SouthAfrica7 strain=SouthAfrica7'
'Helicobacter pylori Shi470 strain=Shi470'
'Helicobacter pylori Puno120 strain=Puno120'
'Helicobacter pylori F16'
'Helicobacter pylori Aklavik117 strain=Aklavik117'
'Helicobacter pylori India7 strain=India7'
'Helicobacter pylori Shi417 strain=Shi417'
'Helicobacter pylori Shi169 strain=Shi169'
'Helicobacter pylori PeCan4 strain=PeCan4'
'Helicobacter pylori Shi112 strain=Shi112'
'Helicobacter pylori strain=ML1'
'Helicobacter pylori SJM180 strain=SJM180'
'Helicobacter pylori 83 strain=83'
'Helicobacter pylori F30'
'Helicobacter pylori strain=Hp238'
'Helicobacter pylori strain=29CaP'

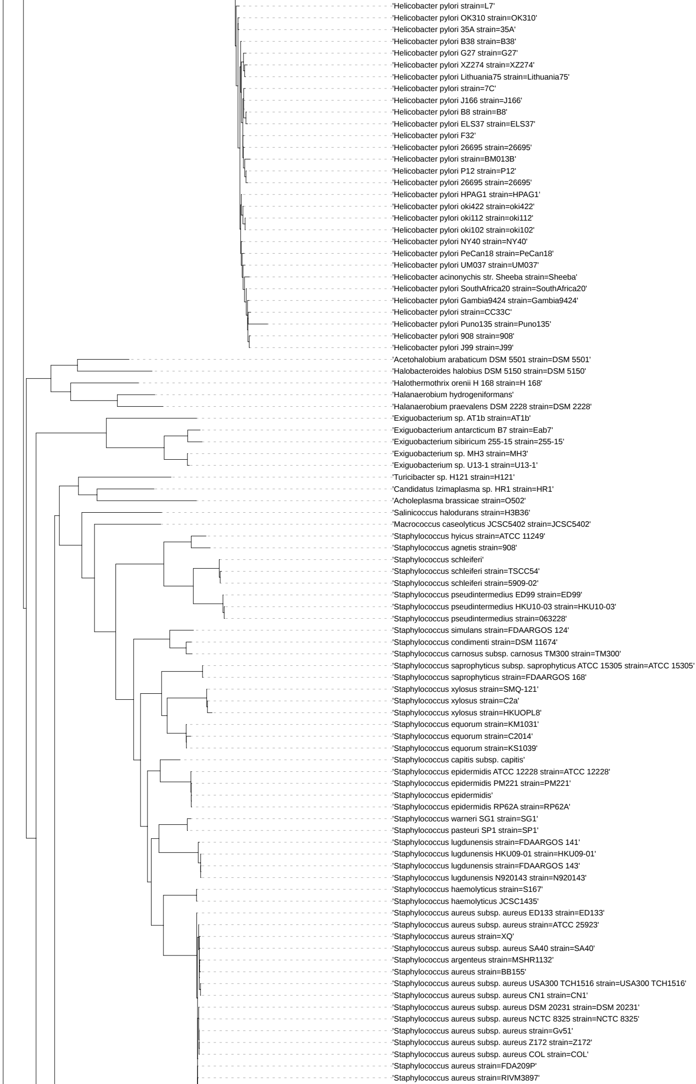
'Helicobacter pylori strain=L7'
'Helicobacter pylori OK310 strain=OK310'
'Helicobacter pylori 35A strain=35A'
'Helicobacter pylori B38 strain=B38'
'Helicobacter pylori G27 strain=G27'
'Helicobacter pylori XZ274 strain=XZ274'
'Helicobacter pylori Lithuania75 strain=Lithuania75'
'Helicobacter pylori strain=7C'
'Helicobacter pylori J166 strain=J166'
'Helicobacter pylori B8 strain=B8'
'Helicobacter pylori ELS37 strain=ELS37'
'Helicobacter pylori F32'
'Helicobacter pylori 26695 strain=26695'
'Helicobacter pylori strain=BM013B'
'Helicobacter pylori P12 strain=P12'
'Helicobacter pylori 26695 strain=26695'
'Helicobacter pylori HPAG1 strain=HPAG1'
'Helicobacter pylori oki422 strain=oki422'
'Helicobacter pylori oki112 strain=oki112'
'Helicobacter pylori oki102 strain=oki102'
'Helicobacter pylori NY40 strain=NY40'
'Helicobacter pylori PeCan18 strain=PeCan18'
'Helicobacter pylori UM037 strain=UM037'
'Helicobacter acinonychis str. Sheeba strain=Sheeba'
'Helicobacter pylori SouthAfrica20 strain=SouthAfrica20'
'Helicobacter pylori Gambia9424 strain=Gambia9424'
'Helicobacter pylori strain=CC33C'
'Helicobacter pylori Puno135 strain=Puno135'
'Helicobacter pylori 908 strain=908'
'Helicobacter pylori J99 strain=J99'
'Acetohalobium arabaticum DSM 5501 strain=DSM 5501'
'Halobacteroides halobius DSM 5150 strain=DSM 5150'
'Halothermothrix orenii H 168 strain=H 168'
'Halanaerobium hydrogeniformans'
'Halanaerobium praevalens DSM 2228 strain=DSM 2228'
'Exiguobacterium sp. AT1b strain=AT1b'
'Exiguobacterium antarcticum B7 strain=Eab7'
'Exiguobacterium sibiricum 255-15 strain=255-15'
'Exiguobacterium sp. MH3 strain=MH3'
'Exiguobacterium sp. U13-1 strain=U13-1'
'Turicibacter sp. H121 strain=H121'
'Candidatus Izimaplasma sp. HR1 strain=HR1'
'Acholeplasma brassicae strain=O502'
'Salinicoccus halodurans strain=H3B36'
'Macrococcus caseolyticus JCSC5402 strain=JCSC5402'
'Staphylococcus hyicus strain=ATCC 11249'
'Staphylococcus agnetis strain=908'
'Staphylococcus schleiferi'
'Staphylococcus schleiferi strain=TSCC54'
'Staphylococcus schleiferi strain=5909-02'
'Staphylococcus pseudintermedius ED99 strain=ED99'
'Staphylococcus pseudintermedius HKU10-03 strain=HKU10-03'
'Staphylococcus pseudintermedius strain=063228'
'Staphylococcus simulans strain=FDAARGOS 124'
'Staphylococcus condimenti strain=DSM 11674'
'Staphylococcus carnosus subsp. carnosus TM300 strain=TM300'
'Staphylococcus saprophyticus subsp. saprophyticus ATCC 15305 strain=ATCC 15305'
'Staphylococcus saprophyticus strain=FDAARGOS 168'
'Staphylococcus xylosus strain=SMQ-121'
'Staphylococcus xylosus strain=C2a'
'Staphylococcus xylosus strain=HKUOPL8'
'Staphylococcus equorum strain=KM1031'
'Staphylococcus equorum strain=C2014'
'Staphylococcus equorum strain=KS1039'
'Staphylococcus capitis subsp. capitis'
'Staphylococcus epidermidis ATCC 12228 strain=ATCC 12228'
'Staphylococcus epidermidis PM221 strain=PM221'
'Staphylococcus epidermidis'
'Staphylococcus epidermidis RP62A strain=RP62A'
'Staphylococcus warneri SG1 strain=SG1'
'Staphylococcus pasteuri SP1 strain=SP1'
'Staphylococcus lugdunensis strain=FDAARGOS 141'
'Staphylococcus lugdunensis HKU09-01 strain=HKU09-01'
'Staphylococcus lugdunensis strain=FDAARGOS 143'
'Staphylococcus lugdunensis N920143 strain=N920143'
'Staphylococcus haemolyticus strain=S167'
'Staphylococcus haemolyticus JCSC1435'
'Staphylococcus aureus subsp. aureus ED133 strain=ED133'
'Staphylococcus aureus subsp. aureus strain=ATCC 25923'
'Staphylococcus aureus strain=XQ'
'Staphylococcus aureus subsp. aureus SA40 strain=SA40'
'Staphylococcus argenteus strain=MSHR1132'
'Staphylococcus aureus strain=BB155'
'Staphylococcus aureus subsp. aureus USA300 TCH1516 strain=USA300 TCH1516'
'Staphylococcus aureus subsp. aureus CN1 strain=CN1'
'Staphylococcus aureus subsp. aureus DSM 20231 strain=DSM 20231'
'Staphylococcus aureus subsp. aureus NCTC 8325 strain=NCTC 8325'
'Staphylococcus aureus subsp. aureus strain=Gv51'
'Staphylococcus aureus subsp. aureus Z172 strain=Z172'
'Staphylococcus aureus subsp. aureus COL strain=COL'
'Staphylococcus aureus strain=FDA209P'
'Staphylococcus aureus strain=RIVM3897'

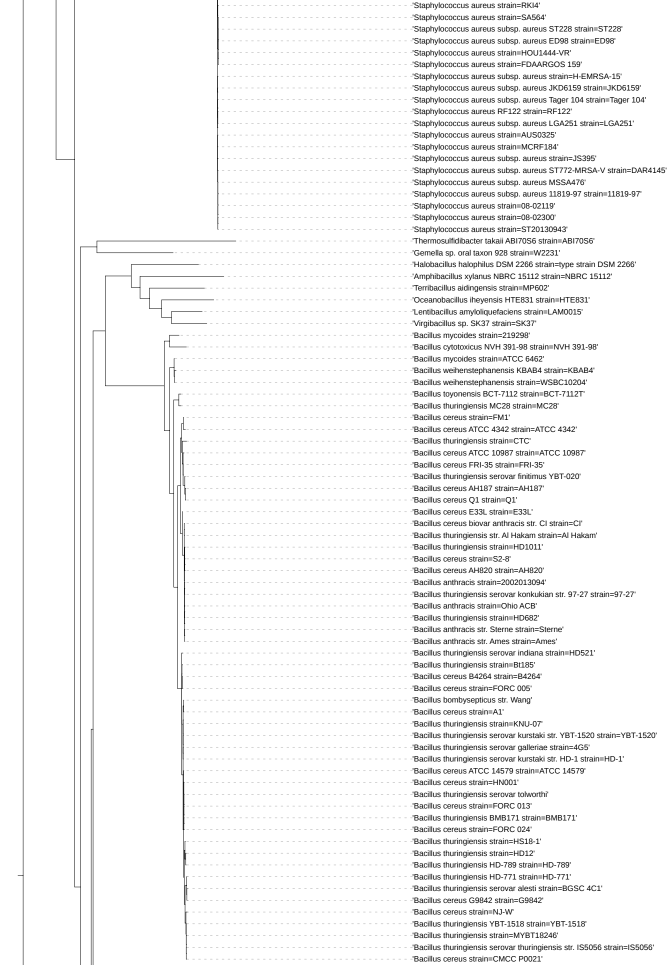
'Staphylococcus aureus strain=RKI4'
'Staphylococcus aureus strain=SA564'
'Staphylococcus aureus subsp. aureus ST228 strain=ST228'
'Staphylococcus aureus subsp. aureus ED98 strain=ED98'
'Staphylococcus aureus strain=HOU1444-VR'
'Staphylococcus aureus strain=FDAARGOS 159'
'Staphylococcus aureus subsp. aureus strain=H-EMRSA-15'
'Staphylococcus aureus subsp. aureus JKD6159 strain=JKD6159'
'Staphylococcus aureus subsp. aureus Tager 104 strain=Tager 104'
'Staphylococcus aureus RF122 strain=RF122'
'Staphylococcus aureus subsp. aureus LGA251 strain=LGA251'
'Staphylococcus aureus strain=AUS0325'
'Staphylococcus aureus strain=MCRF184'
'Staphylococcus aureus subsp. aureus strain=JS395'
'Staphylococcus aureus subsp. aureus ST772-MRSA-V strain=DAR4145'
'Staphylococcus aureus subsp. aureus MSSA476'
'Staphylococcus aureus subsp. aureus 11819-97 strain=11819-97'
'Staphylococcus aureus strain=08-02119'
'Staphylococcus aureus strain=08-02300'
'Staphylococcus aureus strain=ST20130943'
'Thermosulfidibacter takaii ABI70S6 strain=ABI70S6'
'Gemella sp. oral taxon 928 strain=W2231'
'Halobacillus halophilus DSM 2266 strain=type strain DSM 2266'
'Amphibacillus xylanus NBRC 15112 strain=NBRC 15112'
'Terribacillus aidingensis strain=MP602'
'Oceanobacillus iheyensis HTE831 strain=HTE831'
'Lentibacillus amyloliquefaciens strain=LAM0015'
'Virgibacillus sp. SK37 strain=SK37'
'Bacillus mycoides strain=219298'
'Bacillus cytotoxicus NVH 391-98 strain=NVH 391-98'
'Bacillus mycoides strain=ATCC 6462'
'Bacillus weihenstephanensis KBAB4 strain=KBAB4'
'Bacillus weihenstephanensis strain=WSBC10204'
'Bacillus toyonensis BCT-7112 strain=BCT-7112T'
'Bacillus thuringiensis MC28 strain=MC28'
'Bacillus cereus strain=FM1'
'Bacillus cereus ATCC 4342 strain=ATCC 4342'
'Bacillus thuringiensis strain=CTC'
'Bacillus cereus ATCC 10987 strain=ATCC 10987'
'Bacillus cereus FRI-35 strain=FRI-35'
'Bacillus thuringiensis serovar finitimus YBT-020'
'Bacillus cereus AH187 strain=AH187'
'Bacillus cereus Q1 strain=Q1'
'Bacillus cereus E33L strain=E33L'
'Bacillus cereus biovar anthracis str. CI strain=CI'
'Bacillus thuringiensis str. Al Hakam strain=Al Hakam'
'Bacillus thuringiensis strain=HD1011'
'Bacillus cereus strain=S2-8'
'Bacillus cereus AH820 strain=AH820'
'Bacillus anthracis strain=2002013094'
'Bacillus thuringiensis serovar konkukian str. 97-27 strain=97-27'
'Bacillus anthracis strain=Ohio ACB'
'Bacillus thuringiensis strain=HD682'
'Bacillus anthracis str. Sterne strain=Sterne'
'Bacillus anthracis str. Ames strain=Ames'
'Bacillus thuringiensis serovar indiana strain=HD521'
'Bacillus thuringiensis strain=Bt185'
'Bacillus cereus B4264 strain=B4264'
'Bacillus cereus strain=FORC 005'
'Bacillus bombysepticus str. Wang'
'Bacillus cereus strain=A1'
'Bacillus thuringiensis strain=KNU-07'
'Bacillus thuringiensis serovar kurstaki str. YBT-1520 strain=YBT-1520'
'Bacillus thuringiensis serovar galleriae strain=4G5'
'Bacillus thuringiensis serovar kurstaki str. HD-1 strain=HD-1'
'Bacillus cereus ATCC 14579 strain=ATCC 14579'
'Bacillus cereus strain=HN001'
'Bacillus thuringiensis serovar tolworthi'
'Bacillus cereus strain=FORC 013'
'Bacillus thuringiensis BMB171 strain=BMB171'
'Bacillus cereus strain=FORC 024'
'Bacillus thuringiensis strain=HS18-1'
'Bacillus thuringiensis strain=HD12'
'Bacillus thuringiensis HD-789 strain=HD-789'
'Bacillus thuringiensis HD-771 strain=HD-771'
'Bacillus thuringiensis serovar alesti strain=BGSC 4C1'
'Bacillus cereus G9842 strain=G9842'
'Bacillus cereus strain=NJ-W'
'Bacillus thuringiensis YBT-1518 strain=YBT-1518'
'Bacillus thuringiensis strain=MYBT18246'
'Bacillus thuringiensis serovar thuringiensis str. IS5056 strain=IS5056'
'Bacillus cereus strain=CMCC P0021'

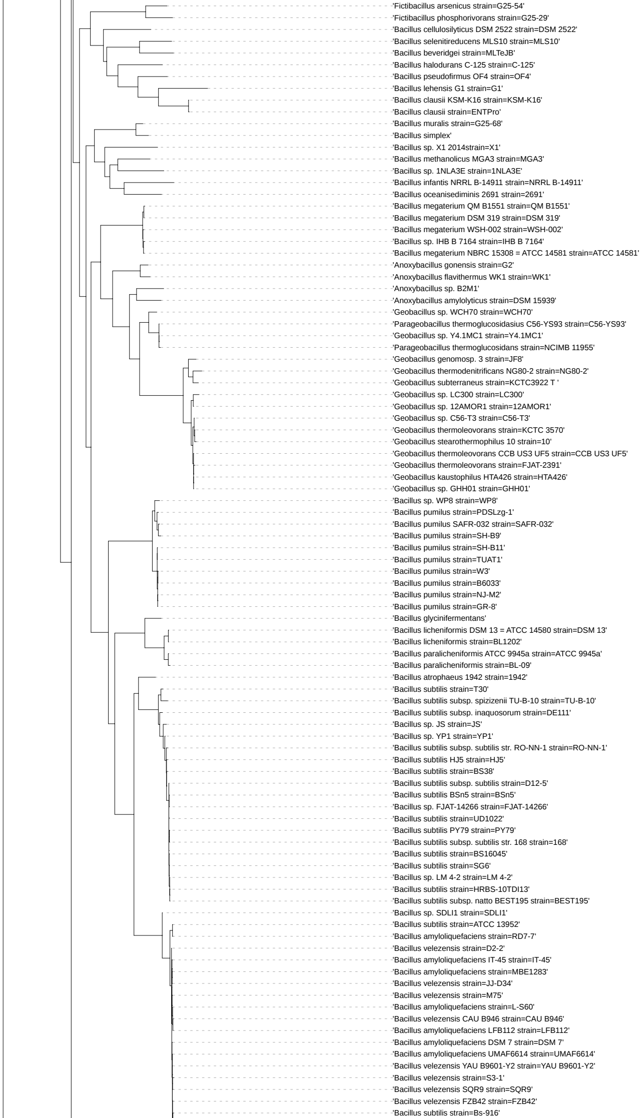

'Fictibacillus arsenicus strain=G25-54'
'Fictibacillus phosphorivorans strain=G25-29'
'Bacillus cellulosilyticus DSM 2522 strain=DSM 2522'
'Bacillus selenitireducens MLS10 strain=MLS10'
'Bacillus beveridgei strain=MLTeJB'
'Bacillus halodurans C-125 strain=C-125'
'Bacillus pseudofirmus OF4 strain=OF4'
'Bacillus lehensis G1 strain=G1'
'Bacillus clausii KSM-K16 strain=KSM-K16'
'Bacillus clausii strain=ENTPro'
'Bacillus muralis strain=G25-68'
'Bacillus simplex'
'Bacillus sp. X1 2014strain=X1'
'Bacillus methanolicus MGA3 strain=MGA3'
'Bacillus sp. 1NLA3E strain=1NLA3E'
'Bacillus infantis NRRL B-14911 strain=NRRL B-14911'
'Bacillus oceanisediminis 2691 strain=2691'
'Bacillus megaterium QM B1551 strain=QM B1551'
'Bacillus megaterium DSM 319 strain=DSM 319'
'Bacillus megaterium WSH-002 strain=WSH-002'
'Bacillus sp. IHB B 7164 strain=IHB B 7164'
'Bacillus megaterium NBRC 15308 = ATCC 14581 strain=ATCC 14581'
'Anoxybacillus gonensis strain=G2'
'Anoxybacillus flavithermus WK1 strain=WK1'
'Anoxybacillus sp. B2M1'
'Anoxybacillus amylolyticus strain=DSM 15939'
'Geobacillus sp. WCH70 strain=WCH70'
'Parageobacillus thermoglucosidasius C56-YS93 strain=C56-YS93'
'Geobacillus sp. Y4.1MC1 strain=Y4.1MC1'
'Parageobacillus thermoglucosidans strain=NCIMB 11955'
'Geobacillus genomosp. 3 strain=JF8'
'Geobacillus thermodenitrificans NG80-2 strain=NG80-2'
'Geobacillus subterraneus strain=KCTC3922 T '
'Geobacillus sp. LC300 strain=LC300'
'Geobacillus sp. 12AMOR1 strain=12AMOR1'
'Geobacillus sp. C56-T3 strain=C56-T3'
'Geobacillus thermoleovorans strain=KCTC 3570'
'Geobacillus stearothermophilus 10 strain=10'
'Geobacillus thermoleovorans CCB US3 UF5 strain=CCB US3 UF5'
'Geobacillus thermoleovorans strain=FJAT-2391'
'Geobacillus kaustophilus HTA426 strain=HTA426'
'Geobacillus sp. GHH01 strain=GHH01'
'Bacillus sp. WP8 strain=WP8'
'Bacillus pumilus strain=PDSLzg-1'
'Bacillus pumilus SAFR-032 strain=SAFR-032'
'Bacillus pumilus strain=SH-B9'
'Bacillus pumilus strain=SH-B11'
'Bacillus pumilus strain=TUAT1'
'Bacillus pumilus strain=W3'
'Bacillus pumilus strain=B6033'
'Bacillus pumilus strain=NJ-M2'
'Bacillus pumilus strain=GR-8'
'Bacillus glycinifermentans'
'Bacillus licheniformis DSM 13 = ATCC 14580 strain=DSM 13'
'Bacillus licheniformis strain=BL1202'
'Bacillus paralicheniformis ATCC 9945a strain=ATCC 9945a'
'Bacillus paralicheniformis strain=BL-09'
'Bacillus atrophaeus 1942 strain=1942'
'Bacillus subtilis strain=T30'
'Bacillus subtilis subsp. spizizenii TU-B-10 strain=TU-B-10'
'Bacillus subtilis subsp. inaquosorum strain=DE111'
'Bacillus sp. JS strain=JS'
'Bacillus sp. YP1 strain=YP1'
'Bacillus subtilis subsp. subtilis str. RO-NN-1 strain=RO-NN-1'
'Bacillus subtilis HJ5 strain=HJ5'
'Bacillus subtilis strain=BS38'
'Bacillus subtilis subsp. subtilis strain=D12-5'
'Bacillus subtilis BSn5 strain=BSn5'
'Bacillus sp. FJAT-14266 strain=FJAT-14266'
'Bacillus subtilis strain=UD1022'
'Bacillus subtilis PY79 strain=PY79'
'Bacillus subtilis subsp. subtilis str. 168 strain=168'
'Bacillus subtilis strain=BS16045'
'Bacillus subtilis strain=SG6'
'Bacillus sp. LM 4-2 strain=LM 4-2'
'Bacillus subtilis strain=HRBS-10TDI13'
'Bacillus subtilis subsp. natto BEST195 strain=BEST195'
'Bacillus sp. SDLI1 strain=SDLI1'
'Bacillus subtilis strain=ATCC 13952'
'Bacillus amyloliquefaciens strain=RD7-7'
'Bacillus velezensis strain=D2-2'
'Bacillus amyloliquefaciens IT-45 strain=IT-45'
'Bacillus amyloliquefaciens strain=MBE1283'
'Bacillus velezensis strain=JJ-D34'
'Bacillus velezensis strain=M75'
'Bacillus amyloliquefaciens strain=L-S60'
'Bacillus velezensis CAU B946 strain=CAU B946'
'Bacillus amyloliquefaciens LFB112 strain=LFB112'
'Bacillus amyloliquefaciens DSM 7 strain=DSM 7'
'Bacillus amyloliquefaciens UMAF6614 strain=UMAF6614'
'Bacillus velezensis YAU B9601-Y2 strain=YAU B9601-Y2'
'Bacillus velezensis strain=S3-1'
'Bacillus velezensis SQR9 strain=SQR9'
'Bacillus velezensis FZB42 strain=FZB42'
'Bacillus subtilis strain=Bs-916'

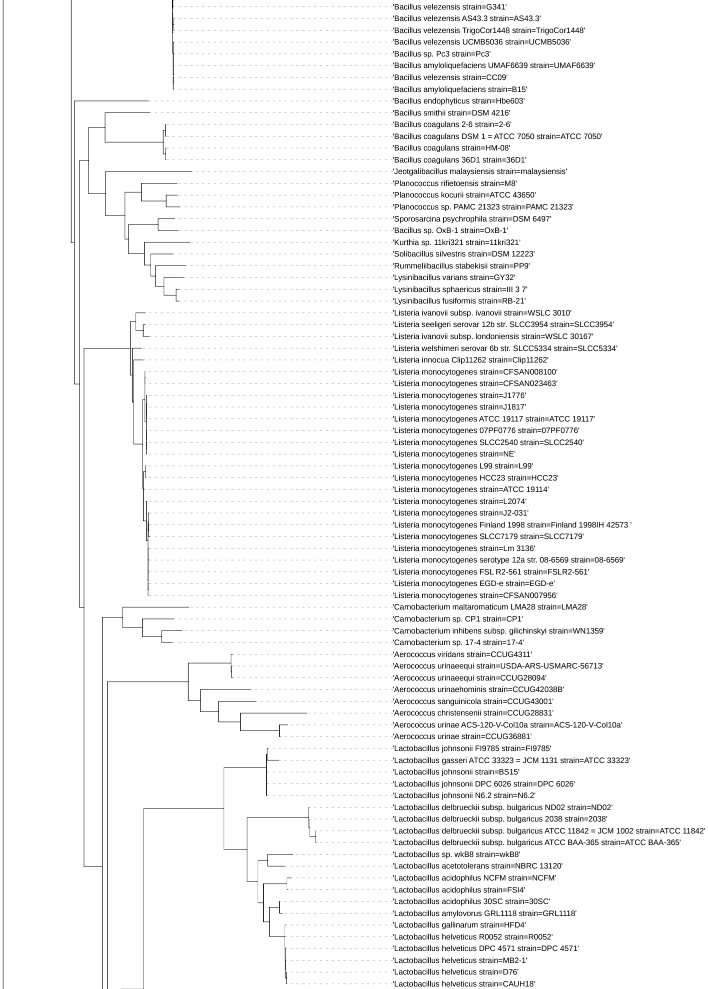
'Bacillus velezensis strain=G341'
'Bacillus velezensis AS43.3 strain=AS43.3'
'Bacillus velezensis TrigoCor1448 strain=TrigoCor1448'
'Bacillus velezensis UCMB5036 strain=UCMB5036'
'Bacillus sp. Pc3 strain=Pc3'
'Bacillus amyloliquefaciens UMAF6639 strain=UMAF6639'
'Bacillus velezensis strain=CC09'
'Bacillus amyloliquefaciens strain=B15'
'Bacillus endophyticus strain=Hbe603'
'Bacillus smithii strain=DSM 4216'
'Bacillus coagulans 2-6 strain=2-6'
'Bacillus coagulans DSM 1 = ATCC 7050 strain=ATCC 7050'
'Bacillus coagulans strain=HM-08'
'Bacillus coagulans 36D1 strain=36D1'
'Jeotgalibacillus malaysiensis strain=malaysiensis'
'Planococcus rifietoensis strain=M8'
'Planococcus kocurii strain=ATCC 43650'
'Planococcus sp. PAMC 21323 strain=PAMC 21323'
'Sporosarcina psychrophila strain=DSM 6497'
'Bacillus sp. OxB-1 strain=OxB-1'
'Kurthia sp. 11kri321 strain=11kri321'
'Solibacillus silvestris strain=DSM 12223'
'Rummeliibacillus stabekisii strain=PP9'
'Lysinibacillus varians strain=GY32'
'Lysinibacillus sphaericus strain=III 3 7'
'Lysinibacillus fusiformis strain=RB-21'
'Listeria ivanovii subsp. ivanovii strain=WSLC 3010'
'Listeria seeligeri serovar 12b str. SLCC3954 strain=SLCC3954'
'Listeria ivanovii subsp. londoniensis strain=WSLC 30167'
'Listeria welshimeri serovar 6b str. SLCC5334 strain=SLCC5334'
'Listeria innocua Clip11262 strain=Clip11262'
'Listeria monocytogenes strain=CFSAN008100'
'Listeria monocytogenes strain=CFSAN023463'
'Listeria monocytogenes strain=J1776'
'Listeria monocytogenes strain=J1817'
'Listeria monocytogenes ATCC 19117 strain=ATCC 19117'
'Listeria monocytogenes 07PF0776 strain=07PF0776'
'Listeria monocytogenes SLCC2540 strain=SLCC2540'
'Listeria monocytogenes strain=NE'
'Listeria monocytogenes L99 strain=L99'
'Listeria monocytogenes HCC23 strain=HCC23'
'Listeria monocytogenes strain=ATCC 19114'
'Listeria monocytogenes strain=L2074'
'Listeria monocytogenes strain=J2-031'
'Listeria monocytogenes Finland 1998 strain=Finland 1998IH 42573 '
'Listeria monocytogenes SLCC7179 strain=SLCC7179'
'Listeria monocytogenes strain=Lm 3136'
'Listeria monocytogenes serotype 12a str. 08-6569 strain=08-6569'
'Listeria monocytogenes FSL R2-561 strain=FSLR2-561'
'Listeria monocytogenes EGD-e strain=EGD-e'
'Listeria monocytogenes strain=CFSAN007956'
'Carnobacterium maltaromaticum LMA28 strain=LMA28'
'Carnobacterium sp. CP1 strain=CP1'
'Carnobacterium inhibens subsp. gilichinskyi strain=WN1359'
'Carnobacterium sp. 17-4 strain=17-4'
'Aerococcus viridans strain=CCUG4311'
'Aerococcus urinaeequi strain=USDA-ARS-USMARC-56713'
'Aerococcus urinaeequi strain=CCUG28094'
'Aerococcus urinaehominis strain=CCUG42038B'
'Aerococcus sanguinicola strain=CCUG43001'
'Aerococcus christensenii strain=CCUG28831'
'Aerococcus urinae ACS-120-V-Col10a strain=ACS-120-V-Col10a'
'Aerococcus urinae strain=CCUG36881'
'Lactobacillus johnsonii FI9785 strain=FI9785'
'Lactobacillus gasseri ATCC 33323 = JCM 1131 strain=ATCC 33323'
'Lactobacillus johnsonii strain=BS15'
'Lactobacillus johnsonii DPC 6026 strain=DPC 6026'
'Lactobacillus johnsonii N6.2 strain=N6.2'
'Lactobacillus delbrueckii subsp. bulgaricus ND02 strain=ND02'
'Lactobacillus delbrueckii subsp. bulgaricus 2038 strain=2038'
'Lactobacillus delbrueckii subsp. bulgaricus ATCC 11842 = JCM 1002 strain=ATCC 11842'
'Lactobacillus delbrueckii subsp. bulgaricus ATCC BAA-365 strain=ATCC BAA-365'
'Lactobacillus sp. wkB8 strain=wkB8'
'Lactobacillus acetotolerans strain=NBRC 13120'
'Lactobacillus acidophilus NCFM strain=NCFM'
'Lactobacillus acidophilus strain=FSI4'
'Lactobacillus acidophilus 30SC strain=30SC'
'Lactobacillus amylovorus GRL1118 strain=GRL1118'
'Lactobacillus gallinarum strain=HFD4'
'Lactobacillus helveticus R0052 strain=R0052'
'Lactobacillus helveticus DPC 4571 strain=DPC 4571'
'Lactobacillus helveticus strain=MB2-1'
'Lactobacillus helveticus strain=D76'
'Lactobacillus helveticus strain=CAUH18'

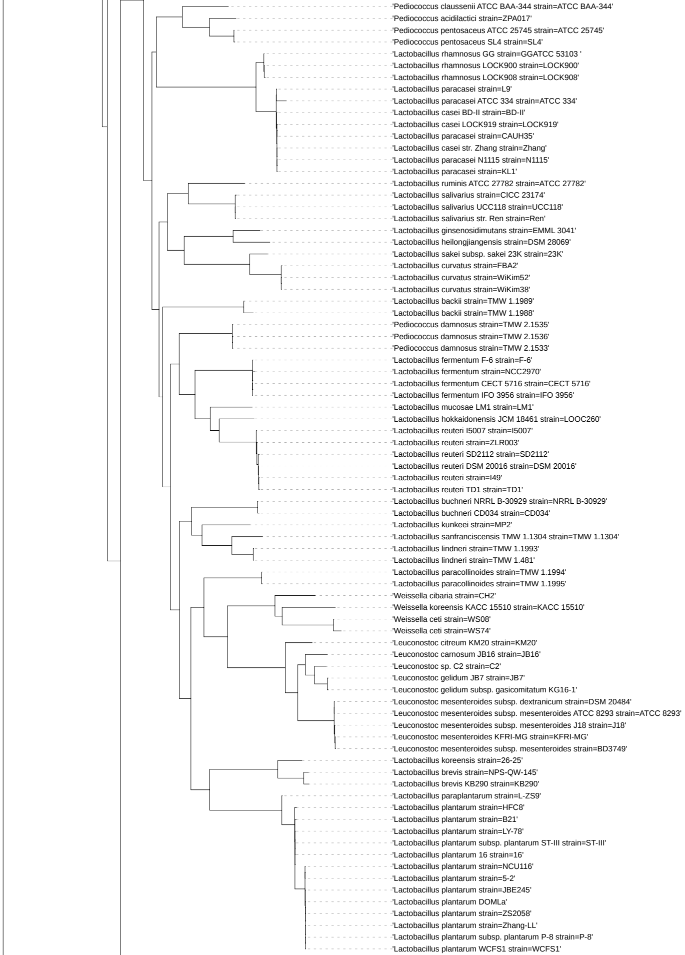
'Pediococcus claussenii ATCC BAA-344 strain=ATCC BAA-344'
'Pediococcus acidilactici strain=ZPA017'
'Pediococcus pentosaceus ATCC 25745 strain=ATCC 25745'
'Pediococcus pentosaceus SL4 strain=SL4'
'Lactobacillus rhamnosus GG strain=GGATCC 53103 '
'Lactobacillus rhamnosus LOCK900 strain=LOCK900'
'Lactobacillus rhamnosus LOCK908 strain=LOCK908'
'Lactobacillus paracasei strain=L9'
'Lactobacillus paracasei ATCC 334 strain=ATCC 334'
'Lactobacillus casei BD-II strain=BD-II'
'Lactobacillus casei LOCK919 strain=LOCK919'
'Lactobacillus paracasei strain=CAUH35'
'Lactobacillus casei str. Zhang strain=Zhang'
'Lactobacillus paracasei N1115 strain=N1115'
'Lactobacillus paracasei strain=KL1'
'Lactobacillus ruminis ATCC 27782 strain=ATCC 27782'
'Lactobacillus salivarius strain=CICC 23174'
'Lactobacillus salivarius UCC118 strain=UCC118'
'Lactobacillus salivarius str. Ren strain=Ren'
'Lactobacillus ginsenosidimutans strain=EMML 3041'
'Lactobacillus heilongjiangensis strain=DSM 28069'
'Lactobacillus sakei subsp. sakei 23K strain=23K'
'Lactobacillus curvatus strain=FBA2'
'Lactobacillus curvatus strain=WiKim52'
'Lactobacillus curvatus strain=WiKim38'
'Lactobacillus backii strain=TMW 1.1989'
'Lactobacillus backii strain=TMW 1.1988'
'Pediococcus damnosus strain=TMW 2.1535'
'Pediococcus damnosus strain=TMW 2.1536'
'Pediococcus damnosus strain=TMW 2.1533'
'Lactobacillus fermentum F-6 strain=F-6'
'Lactobacillus fermentum strain=NCC2970'
'Lactobacillus fermentum CECT 5716 strain=CECT 5716'
'Lactobacillus fermentum IFO 3956 strain=IFO 3956'
'Lactobacillus mucosae LM1 strain=LM1'
'Lactobacillus hokkaidonensis JCM 18461 strain=LOOC260'
'Lactobacillus reuteri I5007 strain=I5007'
'Lactobacillus reuteri strain=ZLR003'
'Lactobacillus reuteri SD2112 strain=SD2112'
'Lactobacillus reuteri DSM 20016 strain=DSM 20016'
'Lactobacillus reuteri strain=I49'
'Lactobacillus reuteri TD1 strain=TD1'
'Lactobacillus buchneri NRRL B-30929 strain=NRRL B-30929'
'Lactobacillus buchneri CD034 strain=CD034'
'Lactobacillus kunkeei strain=MP2'
'Lactobacillus sanfranciscensis TMW 1.1304 strain=TMW 1.1304'
'Lactobacillus lindneri strain=TMW 1.1993'
'Lactobacillus lindneri strain=TMW 1.481'
'Lactobacillus paracollinoides strain=TMW 1.1994'
'Lactobacillus paracollinoides strain=TMW 1.1995'
'Weissella cibaria strain=CH2'
'Weissella koreensis KACC 15510 strain=KACC 15510'
'Weissella ceti strain=WS08'
'Weissella ceti strain=WS74'
'Leuconostoc citreum KM20 strain=KM20'
'Leuconostoc carnosum JB16 strain=JB16'
'Leuconostoc sp. C2 strain=C2'
'Leuconostoc gelidum JB7 strain=JB7'
'Leuconostoc gelidum subsp. gasicomitatum KG16-1'
'Leuconostoc mesenteroides subsp. dextranicum strain=DSM 20484'
'Leuconostoc mesenteroides subsp. mesenteroides ATCC 8293 strain=ATCC 8293'
'Leuconostoc mesenteroides subsp. mesenteroides J18 strain=J18'
'Leuconostoc mesenteroides KFRI-MG strain=KFRI-MG'
'Leuconostoc mesenteroides subsp. mesenteroides strain=BD3749'
'Lactobacillus koreensis strain=26-25'
'Lactobacillus brevis strain=NPS-QW-145'
'Lactobacillus brevis KB290 strain=KB290'
'Lactobacillus paraplantarum strain=L-ZS9'
'Lactobacillus plantarum strain=HFC8'
'Lactobacillus plantarum strain=B21'
'Lactobacillus plantarum strain=LY-78'
'Lactobacillus plantarum subsp. plantarum ST-III strain=ST-III'
'Lactobacillus plantarum 16 strain=16'
'Lactobacillus plantarum strain=NCU116'
'Lactobacillus plantarum strain=5-2'
'Lactobacillus plantarum strain=JBE245'
'Lactobacillus plantarum DOMLa'
'Lactobacillus plantarum strain=ZS2058'
'Lactobacillus plantarum strain=Zhang-LL'
'Lactobacillus plantarum subsp. plantarum P-8 strain=P-8'
'Lactobacillus plantarum WCFS1 strain=WCFS1'

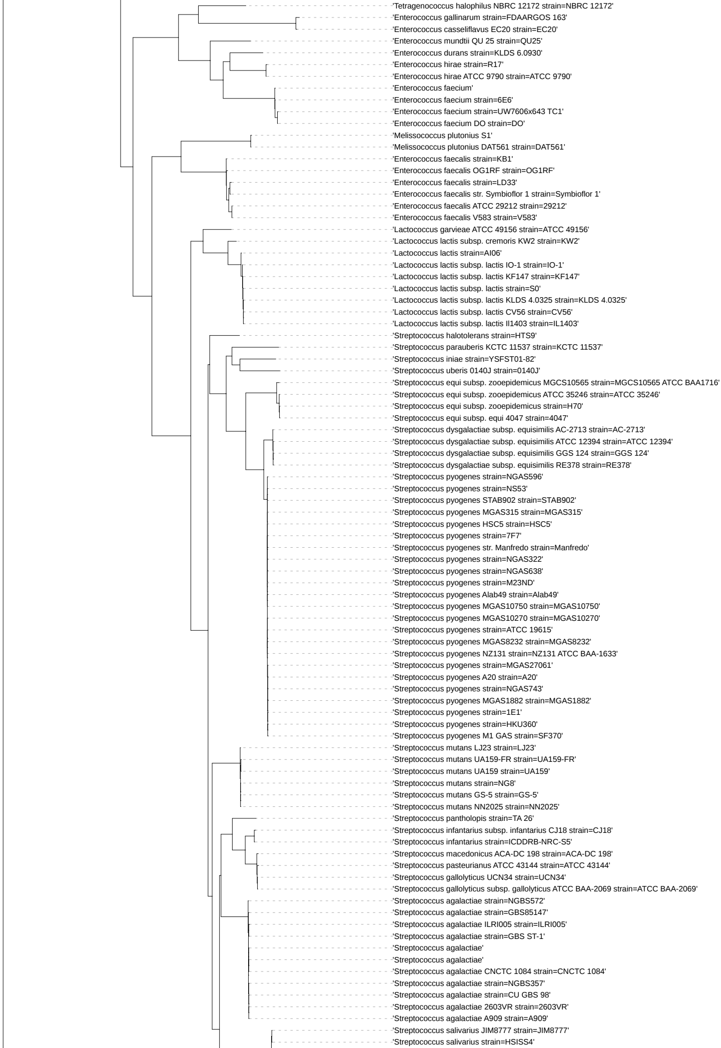

'Tetragenococcus halophilus NBRC 12172 strain=NBRC 12172'
'Enterococcus gallinarum strain=FDAARGOS 163'
'Enterococcus casseliflavus EC20 strain=EC20'
'Enterococcus mundtii QU 25 strain=QU25'
'Enterococcus durans strain=KLDS 6.0930'
'Enterococcus hirae strain=R17'
'Enterococcus hirae ATCC 9790 strain=ATCC 9790'
'Enterococcus faecium'
'Enterococcus faecium strain=6E6'
'Enterococcus faecium strain=UW7606x643 TC1'
'Enterococcus faecium DO strain=DO'
'Melissococcus plutonius S1'
'Melissococcus plutonius DAT561 strain=DAT561'
'Enterococcus faecalis strain=KB1'
'Enterococcus faecalis OG1RF strain=OG1RF'
'Enterococcus faecalis strain=LD33'
'Enterococcus faecalis str. Symbioflor 1 strain=Symbioflor 1'
'Enterococcus faecalis ATCC 29212 strain=29212'
'Enterococcus faecalis V583 strain=V583'
'Lactococcus garvieae ATCC 49156 strain=ATCC 49156'
'Lactococcus lactis subsp. cremoris KW2 strain=KW2'
'Lactococcus lactis strain=AI06'
'Lactococcus lactis subsp. lactis IO-1 strain=IO-1'
'Lactococcus lactis subsp. lactis KF147 strain=KF147'
'Lactococcus lactis subsp. lactis strain=S0'
'Lactococcus lactis subsp. lactis KLDS 4.0325 strain=KLDS 4.0325'
'Lactococcus lactis subsp. lactis CV56 strain=CV56'
'Lactococcus lactis subsp. lactis Il1403 strain=IL1403'
'Streptococcus halotolerans strain=HTS9'
'Streptococcus parauberis KCTC 11537 strain=KCTC 11537'
'Streptococcus iniae strain=YSFST01-82'
'Streptococcus uberis 0140J strain=0140J'
'Streptococcus equi subsp. zooepidemicus MGCS10565 strain=MGCS10565 ATCC BAA1716'
'Streptococcus equi subsp. zooepidemicus ATCC 35246 strain=ATCC 35246'
'Streptococcus equi subsp. zooepidemicus strain=H70'
'Streptococcus equi subsp. equi 4047 strain=4047'
'Streptococcus dysgalactiae subsp. equisimilis AC-2713 strain=AC-2713'
'Streptococcus dysgalactiae subsp. equisimilis ATCC 12394 strain=ATCC 12394'
'Streptococcus dysgalactiae subsp. equisimilis GGS 124 strain=GGS 124'
'Streptococcus dysgalactiae subsp. equisimilis RE378 strain=RE378'
'Streptococcus pyogenes strain=NGAS596'
'Streptococcus pyogenes strain=NS53'
'Streptococcus pyogenes STAB902 strain=STAB902'
'Streptococcus pyogenes MGAS315 strain=MGAS315'
'Streptococcus pyogenes HSC5 strain=HSC5'
'Streptococcus pyogenes strain=7F7'
'Streptococcus pyogenes str. Manfredo strain=Manfredo'
'Streptococcus pyogenes strain=NGAS322'
'Streptococcus pyogenes strain=NGAS638'
'Streptococcus pyogenes strain=M23ND'
'Streptococcus pyogenes Alab49 strain=Alab49'
'Streptococcus pyogenes MGAS10750 strain=MGAS10750'
'Streptococcus pyogenes MGAS10270 strain=MGAS10270'
'Streptococcus pyogenes strain=ATCC 19615'
'Streptococcus pyogenes MGAS8232 strain=MGAS8232'
'Streptococcus pyogenes NZ131 strain=NZ131 ATCC BAA-1633'
'Streptococcus pyogenes strain=MGAS27061'
'Streptococcus pyogenes A20 strain=A20'
'Streptococcus pyogenes strain=NGAS743'
'Streptococcus pyogenes MGAS1882 strain=MGAS1882'
'Streptococcus pyogenes strain=1E1'
'Streptococcus pyogenes strain=HKU360'
'Streptococcus pyogenes M1 GAS strain=SF370'
'Streptococcus mutans LJ23 strain=LJ23'
'Streptococcus mutans UA159-FR strain=UA159-FR'
'Streptococcus mutans UA159 strain=UA159'
'Streptococcus mutans strain=NG8'
'Streptococcus mutans GS-5 strain=GS-5'
'Streptococcus mutans NN2025 strain=NN2025'
'Streptococcus pantholopis strain=TA 26'
'Streptococcus infantarius subsp. infantarius CJ18 strain=CJ18'
'Streptococcus infantarius strain=ICDDRB-NRC-S5'
'Streptococcus macedonicus ACA-DC 198 strain=ACA-DC 198'
'Streptococcus pasteurianus ATCC 43144 strain=ATCC 43144'
'Streptococcus gallolyticus UCN34 strain=UCN34'
'Streptococcus gallolyticus subsp. gallolyticus ATCC BAA-2069 strain=ATCC BAA-2069'
'Streptococcus agalactiae strain=NGBS572'
'Streptococcus agalactiae strain=GBS85147'
'Streptococcus agalactiae ILRI005 strain=ILRI005'
'Streptococcus agalactiae strain=GBS ST-1'
'Streptococcus agalactiae'
'Streptococcus agalactiae'
'Streptococcus agalactiae CNCTC 1084 strain=CNCTC 1084'
'Streptococcus agalactiae strain=NGBS357'
'Streptococcus agalactiae strain=CU GBS 98'
'Streptococcus agalactiae 2603VR strain=2603VR'
'Streptococcus agalactiae A909 strain=A909'
'Streptococcus salivarius JIM8777 strain=JIM8777'
'Streptococcus salivarius strain=HSISS4'

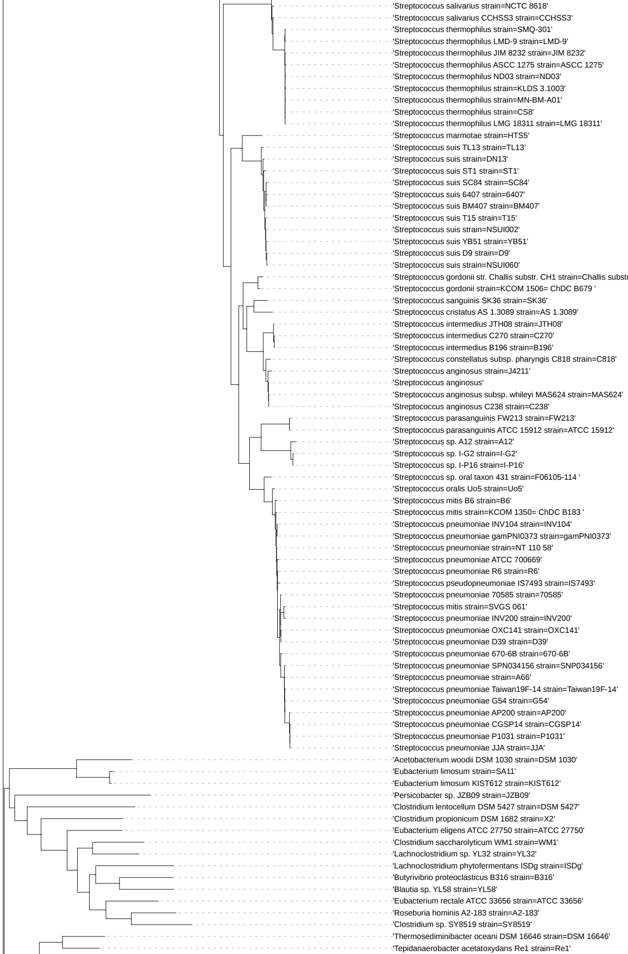
'Streptococcus salivarius strain=NCTC 8618'
'Streptococcus salivarius CCHSS3 strain=CCHSS3'
'Streptococcus thermophilus strain=SMQ-301'
'Streptococcus thermophilus LMD-9 strain=LMD-9'
'Streptococcus thermophilus JIM 8232 strain=JIM 8232'
'Streptococcus thermophilus ASCC 1275 strain=ASCC 1275'
'Streptococcus thermophilus ND03 strain=ND03'
'Streptococcus thermophilus strain=KLDS 3.1003'
'Streptococcus thermophilus strain=MN-BM-A01'
'Streptococcus thermophilus strain=CS8'
'Streptococcus thermophilus LMG 18311 strain=LMG 18311'
'Streptococcus marmotae strain=HTS5'
'Streptococcus suis TL13 strain=TL13'
'Streptococcus suis strain=DN13'
'Streptococcus suis ST1 strain=ST1'
'Streptococcus suis SC84 strain=SC84'
'Streptococcus suis 6407 strain=6407'
'Streptococcus suis BM407 strain=BM407'
'Streptococcus suis T15 strain=T15'
'Streptococcus suis strain=NSUI002'
'Streptococcus suis YB51 strain=YB51'
'Streptococcus suis D9 strain=D9'
'Streptococcus suis strain=NSUI060'
'Streptococcus gordonii str. Challis substr. CH1 strain=Challis substr. CH1'
'Streptococcus gordonii strain=KCOM 1506= ChDC B679 '
'Streptococcus sanguinis SK36 strain=SK36'
'Streptococcus cristatus AS 1.3089 strain=AS 1.3089'
'Streptococcus intermedius JTH08 strain=JTH08'
'Streptococcus intermedius C270 strain=C270'
'Streptococcus intermedius B196 strain=B196'
'Streptococcus constellatus subsp. pharyngis C818 strain=C818'
'Streptococcus anginosus strain=J4211'
'Streptococcus anginosus'
'Streptococcus anginosus subsp. whileyi MAS624 strain=MAS624'
'Streptococcus anginosus C238 strain=C238'
'Streptococcus parasanguinis FW213 strain=FW213'
'Streptococcus parasanguinis ATCC 15912 strain=ATCC 15912'
'Streptococcus sp. A12 strain=A12'
'Streptococcus sp. I-G2 strain=I-G2'
'Streptococcus sp. I-P16 strain=I-P16'
'Streptococcus sp. oral taxon 431 strain=F06105-114 '
'Streptococcus oralis Uo5 strain=Uo5'
'Streptococcus mitis B6 strain=B6'
'Streptococcus mitis strain=KCOM 1350= ChDC B183 '
'Streptococcus pneumoniae INV104 strain=INV104'
'Streptococcus pneumoniae gamPNI0373 strain=gamPNI0373'
'Streptococcus pneumoniae strain=NT 110 58'
'Streptococcus pneumoniae ATCC 700669'
'Streptococcus pneumoniae R6 strain=R6'
'Streptococcus pseudopneumoniae IS7493 strain=IS7493'
'Streptococcus pneumoniae 70585 strain=70585'
'Streptococcus mitis strain=SVGS 061'
'Streptococcus pneumoniae INV200 strain=INV200'
'Streptococcus pneumoniae OXC141 strain=OXC141'
'Streptococcus pneumoniae D39 strain=D39'
'Streptococcus pneumoniae 670-6B strain=670-6B'
'Streptococcus pneumoniae SPN034156 strain=SNP034156'
'Streptococcus pneumoniae strain=A66'
'Streptococcus pneumoniae Taiwan19F-14 strain=Taiwan19F-14'
'Streptococcus pneumoniae G54 strain=G54'
'Streptococcus pneumoniae AP200 strain=AP200'
'Streptococcus pneumoniae CGSP14 strain=CGSP14'
'Streptococcus pneumoniae P1031 strain=P1031'
'Streptococcus pneumoniae JJA strain=JJA'
'Acetobacterium woodii DSM 1030 strain=DSM 1030'
'Eubacterium limosum strain=SA11'
'Eubacterium limosum KIST612 strain=KIST612'
'Persicobacter sp. JZB09 strain=JZB09'
'Clostridium lentocellum DSM 5427 strain=DSM 5427'
'Clostridium propionicum DSM 1682 strain=X2'
'Eubacterium eligens ATCC 27750 strain=ATCC 27750'
'Clostridium saccharolyticum WM1 strain=WM1'
'Lachnoclostridium sp. YL32 strain=YL32'
'Lachnoclostridium phytofermentans ISDg strain=ISDg'
'Butyrivibrio proteoclasticus B316 strain=B316'
'Blautia sp. YL58 strain=YL58'
'Eubacterium rectale ATCC 33656 strain=ATCC 33656'
'Roseburia hominis A2-183 strain=A2-183'
'Clostridium sp. SY8519 strain=SY8519'
'Thermosediminibacter oceani DSM 16646 strain=DSM 16646'
'Tepidanaerobacter acetatoxydans Re1 strain=Re1'

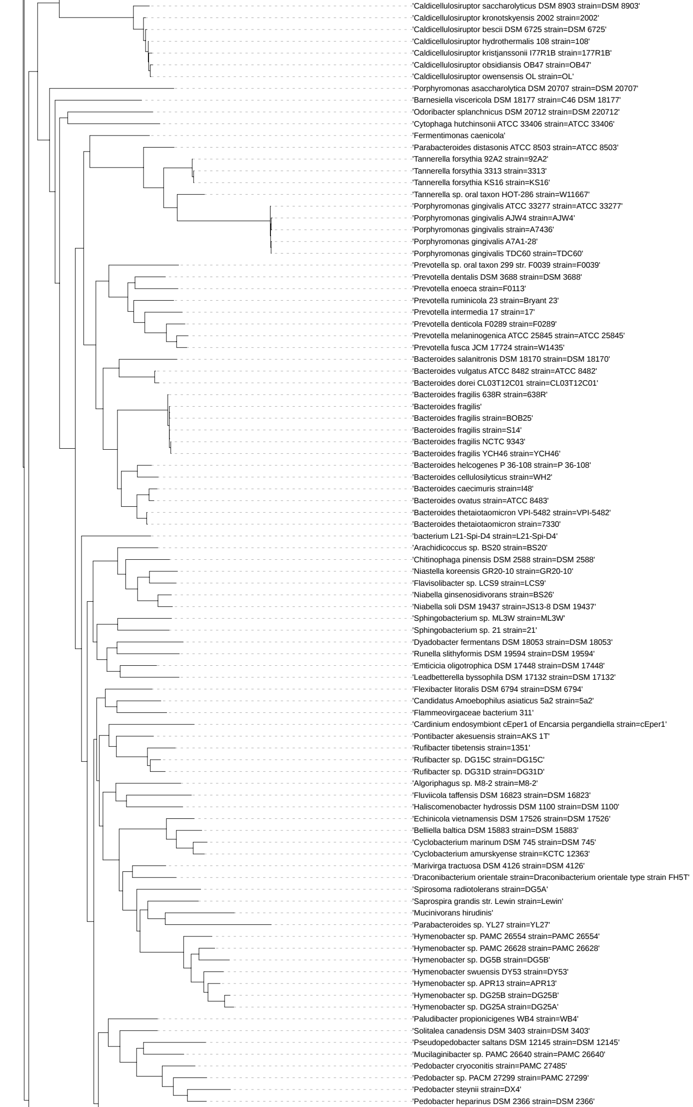
'Caldicellulosiruptor saccharolyticus DSM 8903 strain=DSM 8903'
'Caldicellulosiruptor kronotskyensis 2002 strain=2002'
'Caldicellulosiruptor bescii DSM 6725 strain=DSM 6725'
'Caldicellulosiruptor hydrothermalis 108 strain=108'
'Caldicellulosiruptor kristjanssonii I77R1B strain=177R1B'
'Caldicellulosiruptor obsidiansis OB47 strain=OB47'
'Caldicellulosiruptor owensensis OL strain=OL'
'Porphyromonas asaccharolytica DSM 20707 strain=DSM 20707'
'Barnesiella viscericola DSM 18177 strain=C46 DSM 18177'
'Odoribacter splanchnicus DSM 20712 strain=DSM 220712'
'Cytophaga hutchinsonii ATCC 33406 strain=ATCC 33406'
'Fermentimonas caenicola'
'Parabacteroides distasonis ATCC 8503 strain=ATCC 8503'
'Tannerella forsythia 92A2 strain=92A2'
'Tannerella forsythia 3313 strain=3313'
'Tannerella forsythia KS16 strain=KS16'
'Tannerella sp. oral taxon HOT-286 strain=W11667'
'Porphyromonas gingivalis ATCC 33277 strain=ATCC 33277'
'Porphyromonas gingivalis AJW4 strain=AJW4'
'Porphyromonas gingivalis strain=A7436'
'Porphyromonas gingivalis A7A1-28'
'Porphyromonas gingivalis TDC60 strain=TDC60'
'Prevotella sp. oral taxon 299 str. F0039 strain=F0039'
'Prevotella dentalis DSM 3688 strain=DSM 3688'
'Prevotella enoeca strain=F0113'
'Prevotella ruminicola 23 strain=Bryant 23'
'Prevotella intermedia 17 strain=17'
'Prevotella denticola F0289 strain=F0289'
'Prevotella melaninogenica ATCC 25845 strain=ATCC 25845'
'Prevotella fusca JCM 17724 strain=W1435'
'Bacteroides salanitronis DSM 18170 strain=DSM 18170'
'Bacteroides vulgatus ATCC 8482 strain=ATCC 8482'
'Bacteroides dorei CL03T12C01 strain=CL03T12C01'
'Bacteroides fragilis 638R strain=638R'
'Bacteroides fragilis'
'Bacteroides fragilis strain=BOB25'
'Bacteroides fragilis strain=S14'
'Bacteroides fragilis NCTC 9343'
'Bacteroides fragilis YCH46 strain=YCH46'
'Bacteroides helcogenes P 36-108 strain=P 36-108'
'Bacteroides cellulosilyticus strain=WH2'
'Bacteroides caecimuris strain=I48'
'Bacteroides ovatus strain=ATCC 8483'
'Bacteroides thetaiotaomicron VPI-5482 strain=VPI-5482'
'Bacteroides thetaiotaomicron strain=7330'
'bacterium L21-Spi-D4 strain=L21-Spi-D4'
'Arachidicoccus sp. BS20 strain=BS20'
'Chitinophaga pinensis DSM 2588 strain=DSM 2588'
'Niastella koreensis GR20-10 strain=GR20-10'
'Flavisolibacter sp. LCS9 strain=LCS9'
'Niabella ginsenosidivorans strain=BS26'
'Niabella soli DSM 19437 strain=JS13-8 DSM 19437'
'Sphingobacterium sp. ML3W strain=ML3W'
'Sphingobacterium sp. 21 strain=21'
'Dyadobacter fermentans DSM 18053 strain=DSM 18053'
'Runella slithyformis DSM 19594 strain=DSM 19594'
'Emticicia oligotrophica DSM 17448 strain=DSM 17448'
'Leadbetterella byssophila DSM 17132 strain=DSM 17132'
'Flexibacter litoralis DSM 6794 strain=DSM 6794'
'Candidatus Amoebophilus asiaticus 5a2 strain=5a2'
'Flammeovirgaceae bacterium 311'
'Cardinium endosymbiont cEper1 of Encarsia pergandiella strain=cEper1'
'Pontibacter akesuensis strain=AKS 1T'
'Rufibacter tibetensis strain=1351'
'Rufibacter sp. DG15C strain=DG15C'
'Rufibacter sp. DG31D strain=DG31D'
'Algoriphagus sp. M8-2 strain=M8-2'
'Fluviicola taffensis DSM 16823 strain=DSM 16823'
'Haliscomenobacter hydrossis DSM 1100 strain=DSM 1100'
'Echinicola vietnamensis DSM 17526 strain=DSM 17526'
'Belliella baltica DSM 15883 strain=DSM 15883'
'Cyclobacterium marinum DSM 745 strain=DSM 745'
'Cyclobacterium amurskyense strain=KCTC 12363'
'Marivirga tractuosa DSM 4126 strain=DSM 4126'
'Draconibacterium orientale strain=Draconibacterium orientale type strain FH5T'
'Spirosoma radiotolerans strain=DG5A'
'Saprospira grandis str. Lewin strain=Lewin'
'Mucinivorans hirudinis'
'Parabacteroides sp. YL27 strain=YL27'
'Hymenobacter sp. PAMC 26554 strain=PAMC 26554'
'Hymenobacter sp. PAMC 26628 strain=PAMC 26628'
'Hymenobacter sp. DG5B strain=DG5B'
'Hymenobacter swuensis DY53 strain=DY53'
'Hymenobacter sp. APR13 strain=APR13'
'Hymenobacter sp. DG25B strain=DG25B'
'Hymenobacter sp. DG25A strain=DG25A'
'Paludibacter propionicigenes WB4 strain=WB4'
'Solitalea canadensis DSM 3403 strain=DSM 3403'
'Pseudopedobacter saltans DSM 12145 strain=DSM 12145'
'Mucilaginibacter sp. PAMC 26640 strain=PAMC 26640'
'Pedobacter cryoconitis strain=PAMC 27485'
'Pedobacter sp. PACM 27299 strain=PAMC 27299'
'Pedobacter steynii strain=DX4'
'Pedobacter heparinus DSM 2366 strain=DSM 2366'

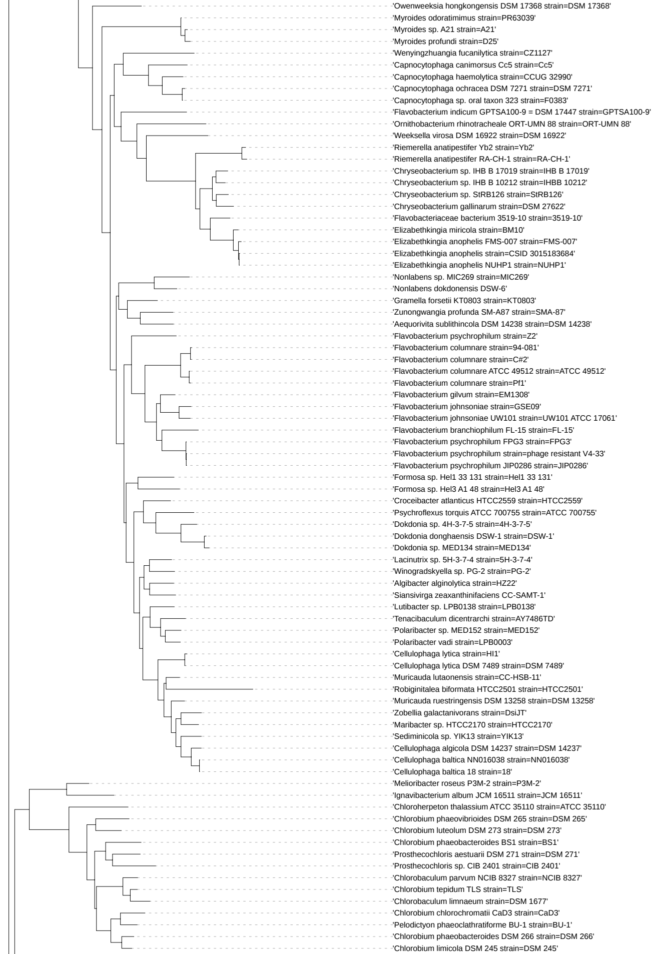
'Owenweeksia hongkongensis DSM 17368 strain=DSM 17368'
'Myroides odoratimimus strain=PR63039'
'Myroides sp. A21 strain=A21'
'Myroides profundi strain=D25'
'Wenyingzhuangia fucanilytica strain=CZ1127'
'Capnocytophaga canimorsus Cc5 strain=Cc5'
'Capnocytophaga haemolytica strain=CCUG 32990'
'Capnocytophaga ochracea DSM 7271 strain=DSM 7271'
'Capnocytophaga sp. oral taxon 323 strain=F0383'
'Flavobacterium indicum GPTSA100-9 = DSM 17447 strain=GPTSA100-9'
'Ornithobacterium rhinotracheale ORT-UMN 88 strain=ORT-UMN 88'
'Weeksella virosa DSM 16922 strain=DSM 16922'
'Riemerella anatipestifer Yb2 strain=Yb2'
'Riemerella anatipestifer RA-CH-1 strain=RA-CH-1'
'Chryseobacterium sp. IHB B 17019 strain=IHB B 17019'
'Chryseobacterium sp. IHB B 10212 strain=IHBB 10212'
'Chryseobacterium sp. StRB126 strain=StRB126'
'Chryseobacterium gallinarum strain=DSM 27622'
'Flavobacteriaceae bacterium 3519-10 strain=3519-10'
'Elizabethkingia miricola strain=BM10'
'Elizabethkingia anophelis FMS-007 strain=FMS-007'
'Elizabethkingia anophelis strain=CSID 3015183684'
'Elizabethkingia anophelis NUHP1 strain=NUHP1'
'Nonlabens sp. MIC269 strain=MIC269'
'Nonlabens dokdonensis DSW-6'
'Gramella forsetii KT0803 strain=KT0803'
'Zunongwangia profunda SM-A87 strain=SMA-87'
'Aequorivita sublithincola DSM 14238 strain=DSM 14238'
'Flavobacterium psychrophilum strain=Z2'
'Flavobacterium columnare strain=94-081'
'Flavobacterium columnare strain=C#2'
'Flavobacterium columnare ATCC 49512 strain=ATCC 49512'
'Flavobacterium columnare strain=Pf1'
'Flavobacterium gilvum strain=EM1308'
'Flavobacterium johnsoniae strain=GSE09'
'Flavobacterium johnsoniae UW101 strain=UW101 ATCC 17061'
'Flavobacterium branchiophilum FL-15 strain=FL-15'
'Flavobacterium psychrophilum FPG3 strain=FPG3'
'Flavobacterium psychrophilum strain=phage resistant V4-33'
'Flavobacterium psychrophilum JIP0286 strain=JIP0286'
'Formosa sp. Hel1 33 131 strain=Hel1 33 131'
'Formosa sp. Hel3 A1 48 strain=Hel3 A1 48'
'Croceibacter atlanticus HTCC2559 strain=HTCC2559'
'Psychroflexus torquis ATCC 700755 strain=ATCC 700755'
'Dokdonia sp. 4H-3-7-5 strain=4H-3-7-5'
'Dokdonia donghaensis DSW-1 strain=DSW-1'
'Dokdonia sp. MED134 strain=MED134'
'Lacinutrix sp. 5H-3-7-4 strain=5H-3-7-4'
'Winogradskyella sp. PG-2 strain=PG-2'
'Algibacter alginolytica strain=HZ22'
'Siansivirga zeaxanthinifaciens CC-SAMT-1'
'Lutibacter sp. LPB0138 strain=LPB0138'
'Tenacibaculum dicentrarchi strain=AY7486TD'
'Polaribacter sp. MED152 strain=MED152'
'Polaribacter vadi strain=LPB0003'
'Cellulophaga lytica strain=HI1'
'Cellulophaga lytica DSM 7489 strain=DSM 7489'
'Muricauda lutaonensis strain=CC-HSB-11'
'Robiginitalea biformata HTCC2501 strain=HTCC2501'
'Muricauda ruestringensis DSM 13258 strain=DSM 13258'
'Zobellia galactanivorans strain=DsiJT'
'Maribacter sp. HTCC2170 strain=HTCC2170'
'Sediminicola sp. YIK13 strain=YIK13'
'Cellulophaga algicola DSM 14237 strain=DSM 14237'
'Cellulophaga baltica NN016038 strain=NN016038'
'Cellulophaga baltica 18 strain=18'
'Melioribacter roseus P3M-2 strain=P3M-2'
'Ignavibacterium album JCM 16511 strain=JCM 16511'
'Chloroherpeton thalassium ATCC 35110 strain=ATCC 35110'
'Chlorobium phaeovibrioides DSM 265 strain=DSM 265'
'Chlorobium luteolum DSM 273 strain=DSM 273'
'Chlorobium phaeobacteroides BS1 strain=BS1'
'Prosthecochloris aestuarii DSM 271 strain=DSM 271'
'Prosthecochloris sp. CIB 2401 strain=CIB 2401'
'Chlorobaculum parvum NCIB 8327 strain=NCIB 8327'
'Chlorobium tepidum TLS strain=TLS'
'Chlorobaculum limnaeum strain=DSM 1677'
'Chlorobium chlorochromatii CaD3 strain=CaD3'
'Pelodictyon phaeoclathratiforme BU-1 strain=BU-1'
'Chlorobium phaeobacteroides DSM 266 strain=DSM 266'
'Chlorobium limicola DSM 245 strain=DSM 245'

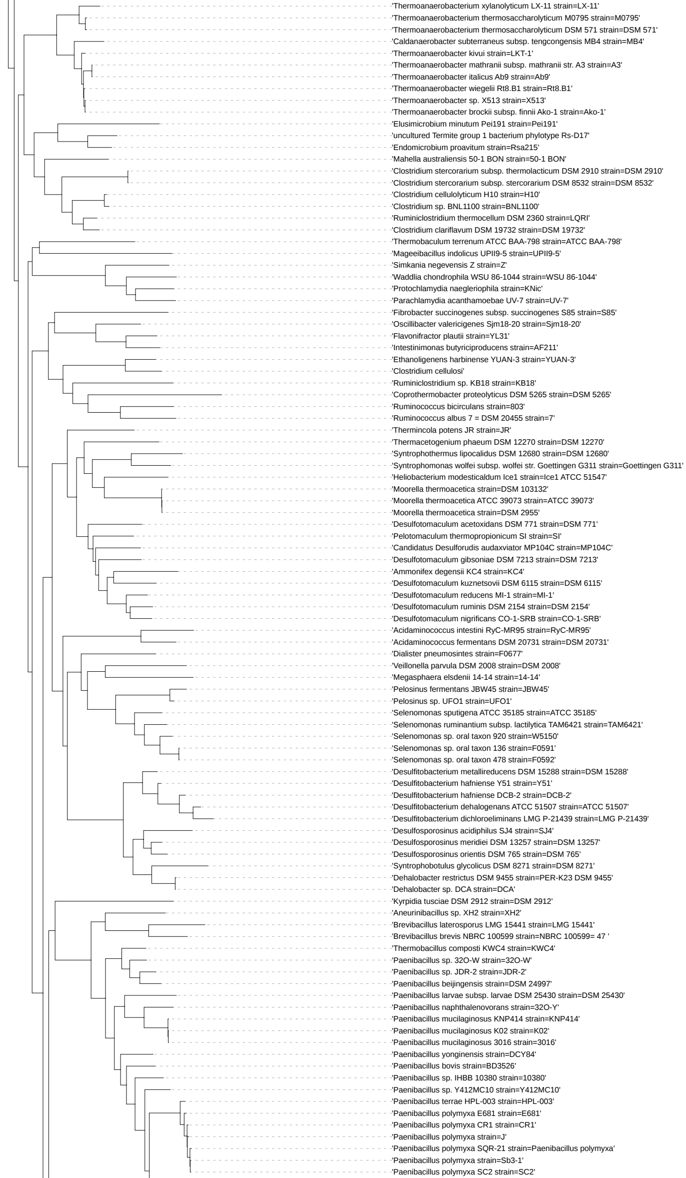
'Thermoanaerobacterium xylanolyticum LX-11 strain=LX-11'
'Thermoanaerobacterium thermosaccharolyticum M0795 strain=M0795'
'Thermoanaerobacterium thermosaccharolyticum DSM 571 strain=DSM 571'
'Caldanaerobacter subterraneus subsp. tengcongensis MB4 strain=MB4'
'Thermoanaerobacter kivui strain=LKT-1'
'Thermoanaerobacter mathranii subsp. mathranii str. A3 strain=A3'
'Thermoanaerobacter italicus Ab9 strain=Ab9'
'Thermoanaerobacter wiegelii Rt8.B1 strain=Rt8.B1'
'Thermoanaerobacter sp. X513 strain=X513'
'Thermoanaerobacter brockii subsp. finnii Ako-1 strain=Ako-1'
'Elusimicrobium minutum Pei191 strain=Pei191'
'uncultured Termite group 1 bacterium phylotype Rs-D17'
'Endomicrobium proavitum strain=Rsa215'
'Mahella australiensis 50-1 BON strain=50-1 BON'
'Clostridium stercorarium subsp. thermolacticum DSM 2910 strain=DSM 2910'
'Clostridium stercorarium subsp. stercorarium DSM 8532 strain=DSM 8532'
'Clostridium cellulolyticum H10 strain=H10'
'Clostridium sp. BNL1100 strain=BNL1100'
'Ruminiclostridium thermocellum DSM 2360 strain=LQRI'
'Clostridium clariflavum DSM 19732 strain=DSM 19732'
'Thermobaculum terrenum ATCC BAA-798 strain=ATCC BAA-798'
'Mageeibacillus indolicus UPII9-5 strain=UPII9-5'
'Simkania negevensis Z strain=Z'
'Waddlia chondrophila WSU 86-1044 strain=WSU 86-1044'
'Protochlamydia naegleriophila strain=KNic'
'Parachlamydia acanthamoebae UV-7 strain=UV-7'
'Fibrobacter succinogenes subsp. succinogenes S85 strain=S85'
'Oscillibacter valericigenes Sjm18-20 strain=Sjm18-20'
'Flavonifractor plautii strain=YL31'
'Intestinimonas butyriciproducens strain=AF211'
'Ethanoligenens harbinense YUAN-3 strain=YUAN-3'
'Clostridium cellulosi'
'Ruminiclostridium sp. KB18 strain=KB18'
'Coprothermobacter proteolyticus DSM 5265 strain=DSM 5265'
'Ruminococcus bicirculans strain=803'
'Ruminococcus albus 7 = DSM 20455 strain=7'
'Thermincola potens JR strain=JR'
'Thermacetogenium phaeum DSM 12270 strain=DSM 12270'
'Syntrophothermus lipocalidus DSM 12680 strain=DSM 12680'
'Syntrophomonas wolfei subsp. wolfei str. Goettingen G311 strain=Goettingen G311'
'Heliobacterium modesticaldum Ice1 strain=Ice1 ATCC 51547'
'Moorella thermoacetica strain=DSM 103132'
'Moorella thermoacetica ATCC 39073 strain=ATCC 39073'
'Moorella thermoacetica strain=DSM 2955'
'Desulfotomaculum acetoxidans DSM 771 strain=DSM 771'
'Pelotomaculum thermopropionicum SI strain=SI'
'Candidatus Desulforudis audaxviator MP104C strain=MP104C'
'Desulfotomaculum gibsoniae DSM 7213 strain=DSM 7213'
'Ammonifex degensii KC4 strain=KC4'
'Desulfotomaculum kuznetsovii DSM 6115 strain=DSM 6115'
'Desulfotomaculum reducens MI-1 strain=MI-1'
'Desulfotomaculum ruminis DSM 2154 strain=DSM 2154'
'Desulfotomaculum nigrificans CO-1-SRB strain=CO-1-SRB'
'Acidaminococcus intestini RyC-MR95 strain=RyC-MR95'
'Acidaminococcus fermentans DSM 20731 strain=DSM 20731'
'Dialister pneumosintes strain=F0677'
'Veillonella parvula DSM 2008 strain=DSM 2008'
'Megasphaera elsdenii 14-14 strain=14-14'
'Pelosinus fermentans JBW45 strain=JBW45'
'Pelosinus sp. UFO1 strain=UFO1'
'Selenomonas sputigena ATCC 35185 strain=ATCC 35185'
'Selenomonas ruminantium subsp. lactilytica TAM6421 strain=TAM6421'
'Selenomonas sp. oral taxon 920 strain=W5150'
'Selenomonas sp. oral taxon 136 strain=F0591'
'Selenomonas sp. oral taxon 478 strain=F0592'
'Desulfitobacterium metallireducens DSM 15288 strain=DSM 15288'
'Desulfitobacterium hafniense Y51 strain=Y51'
'Desulfitobacterium hafniense DCB-2 strain=DCB-2'
'Desulfitobacterium dehalogenans ATCC 51507 strain=ATCC 51507'
'Desulfitobacterium dichloroeliminans LMG P-21439 strain=LMG P-21439'
'Desulfosporosinus acidiphilus SJ4 strain=SJ4'
'Desulfosporosinus meridiei DSM 13257 strain=DSM 13257'
'Desulfosporosinus orientis DSM 765 strain=DSM 765'
'Syntrophobotulus glycolicus DSM 8271 strain=DSM 8271'
'Dehalobacter restrictus DSM 9455 strain=PER-K23 DSM 9455'
'Dehalobacter sp. DCA strain=DCA'
'Kyrpidia tusciae DSM 2912 strain=DSM 2912'
'Aneurinibacillus sp. XH2 strain=XH2'
'Brevibacillus laterosporus LMG 15441 strain=LMG 15441'
'Brevibacillus brevis NBRC 100599 strain=NBRC 100599= 47 '
'Thermobacillus composti KWC4 strain=KWC4'
'Paenibacillus sp. 32O-W strain=32O-W'
'Paenibacillus sp. JDR-2 strain=JDR-2'
'Paenibacillus beijingensis strain=DSM 24997'
'Paenibacillus larvae subsp. larvae DSM 25430 strain=DSM 25430'
'Paenibacillus naphthalenovorans strain=32O-Y'
'Paenibacillus mucilaginosus KNP414 strain=KNP414'
'Paenibacillus mucilaginosus K02 strain=K02'
'Paenibacillus mucilaginosus 3016 strain=3016'
'Paenibacillus yonginensis strain=DCY84'
'Paenibacillus bovis strain=BD3526'
'Paenibacillus sp. IHBB 10380 strain=10380'
'Paenibacillus sp. Y412MC10 strain=Y412MC10'
'Paenibacillus terrae HPL-003 strain=HPL-003'
'Paenibacillus polymyxa E681 strain=E681'
'Paenibacillus polymyxa CR1 strain=CR1'
'Paenibacillus polymyxa strain=J'
'Paenibacillus polymyxa SQR-21 strain=Paenibacillus polymyxa'
'Paenibacillus polymyxa strain=Sb3-1'
'Paenibacillus polymyxa SC2 strain=SC2'

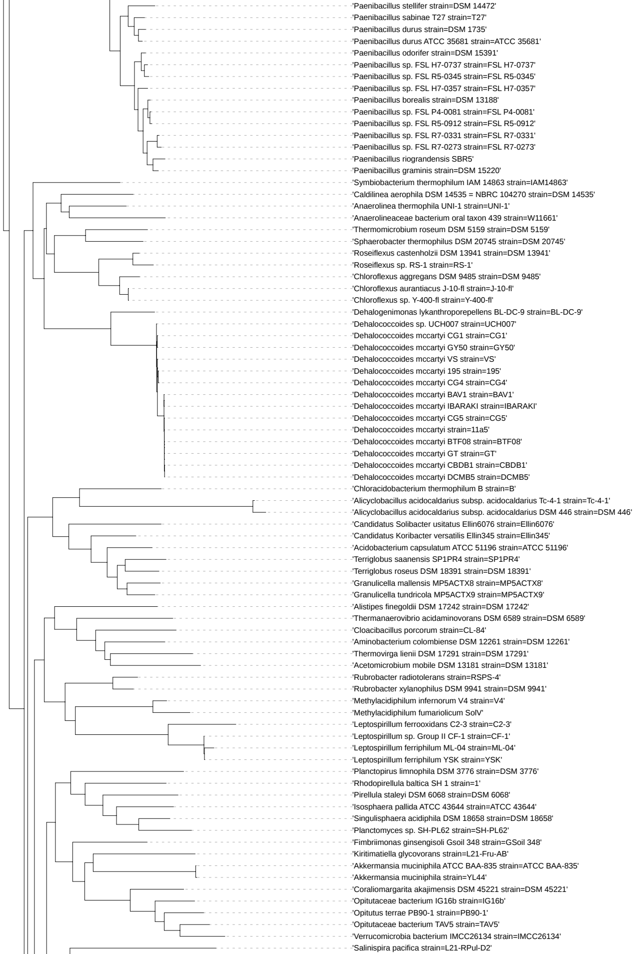
'Paenibacillus stellifer strain=DSM 14472'
'Paenibacillus sabinae T27 strain=T27'
'Paenibacillus durus strain=DSM 1735'
'Paenibacillus durus ATCC 35681 strain=ATCC 35681'
'Paenibacillus odorifer strain=DSM 15391'
'Paenibacillus sp. FSL H7-0737 strain=FSL H7-0737'
'Paenibacillus sp. FSL R5-0345 strain=FSL R5-0345'
'Paenibacillus sp. FSL H7-0357 strain=FSL H7-0357'
'Paenibacillus borealis strain=DSM 13188'
'Paenibacillus sp. FSL P4-0081 strain=FSL P4-0081'
'Paenibacillus sp. FSL R5-0912 strain=FSL R5-0912'
'Paenibacillus sp. FSL R7-0331 strain=FSL R7-0331'
'Paenibacillus sp. FSL R7-0273 strain=FSL R7-0273'
'Paenibacillus riograndensis SBR5'
'Paenibacillus graminis strain=DSM 15220'
'Symbiobacterium thermophilum IAM 14863 strain=IAM14863'
'Caldilinea aerophila DSM 14535 = NBRC 104270 strain=DSM 14535'
'Anaerolinea thermophila UNI-1 strain=UNI-1'
'Anaerolineaceae bacterium oral taxon 439 strain=W11661'
'Thermomicrobium roseum DSM 5159 strain=DSM 5159'
'Sphaerobacter thermophilus DSM 20745 strain=DSM 20745'
'Roseiflexus castenholzii DSM 13941 strain=DSM 13941'
'Roseiflexus sp. RS-1 strain=RS-1'
'Chloroflexus aggregans DSM 9485 strain=DSM 9485'
'Chloroflexus aurantiacus J-10-fl strain=J-10-fl'
'Chloroflexus sp. Y-400-fl strain=Y-400-fl'
'Dehalogenimonas lykanthroporepellens BL-DC-9 strain=BL-DC-9'
'Dehalococcoides sp. UCH007 strain=UCH007'
'Dehalococcoides mccartyi CG1 strain=CG1'
'Dehalococcoides mccartyi GY50 strain=GY50'
'Dehalococcoides mccartyi VS strain=VS'
'Dehalococcoides mccartyi 195 strain=195'
'Dehalococcoides mccartyi CG4 strain=CG4'
'Dehalococcoides mccartyi BAV1 strain=BAV1'
'Dehalococcoides mccartyi IBARAKI strain=IBARAKI'
'Dehalococcoides mccartyi CG5 strain=CG5'
'Dehalococcoides mccartyi strain=11a5'
'Dehalococcoides mccartyi BTF08 strain=BTF08'
'Dehalococcoides mccartyi GT strain=GT'
'Dehalococcoides mccartyi CBDB1 strain=CBDB1'
'Dehalococcoides mccartyi DCMB5 strain=DCMB5'
'Chloracidobacterium thermophilum B strain=B'
'Alicyclobacillus acidocaldarius subsp. acidocaldarius Tc-4-1 strain=Tc-4-1'
'Alicyclobacillus acidocaldarius subsp. acidocaldarius DSM 446 strain=DSM 446'
'Candidatus Solibacter usitatus Ellin6076 strain=Ellin6076'
'Candidatus Koribacter versatilis Ellin345 strain=Ellin345'
'Acidobacterium capsulatum ATCC 51196 strain=ATCC 51196'
'Terriglobus saanensis SP1PR4 strain=SP1PR4'
'Terriglobus roseus DSM 18391 strain=DSM 18391'
'Granulicella mallensis MP5ACTX8 strain=MP5ACTX8'
'Granulicella tundricola MP5ACTX9 strain=MP5ACTX9'
'Alistipes finegoldii DSM 17242 strain=DSM 17242'
'Thermanaerovibrio acidaminovorans DSM 6589 strain=DSM 6589'
'Cloacibacillus porcorum strain=CL-84'
'Aminobacterium colombiense DSM 12261 strain=DSM 12261'
'Thermovirga lienii DSM 17291 strain=DSM 17291'
'Acetomicrobium mobile DSM 13181 strain=DSM 13181'
'Rubrobacter radiotolerans strain=RSPS-4'
'Rubrobacter xylanophilus DSM 9941 strain=DSM 9941'
'Methylacidiphilum infernorum V4 strain=V4'
'Methylacidiphilum fumariolicum SolV'
'Leptospirillum ferrooxidans C2-3 strain=C2-3'
'Leptospirillum sp. Group II CF-1 strain=CF-1'
'Leptospirillum ferriphilum ML-04 strain=ML-04'
'Leptospirillum ferriphilum YSK strain=YSK'
'Planctopirus limnophila DSM 3776 strain=DSM 3776'
'Rhodopirellula baltica SH 1 strain=1'
'Pirellula staleyi DSM 6068 strain=DSM 6068'
'Isosphaera pallida ATCC 43644 strain=ATCC 43644'
'Singulisphaera acidiphila DSM 18658 strain=DSM 18658'
'Planctomyces sp. SH-PL62 strain=SH-PL62'
'Fimbriimonas ginsengisoli Gsoil 348 strain=GSoil 348'
'Kiritimatiella glycovorans strain=L21-Fru-AB'
'Akkermansia muciniphila ATCC BAA-835 strain=ATCC BAA-835'
'Akkermansia muciniphila strain=YL44'
'Coraliomargarita akajimensis DSM 45221 strain=DSM 45221'
'Opitutaceae bacterium IG16b strain=IG16b'
'Opitutus terrae PB90-1 strain=PB90-1'
'Opitutaceae bacterium TAV5 strain=TAV5'
'Verrucomicrobia bacterium IMCC26134 strain=IMCC26134'
'Salinispira pacifica strain=L21-RPul-D2'

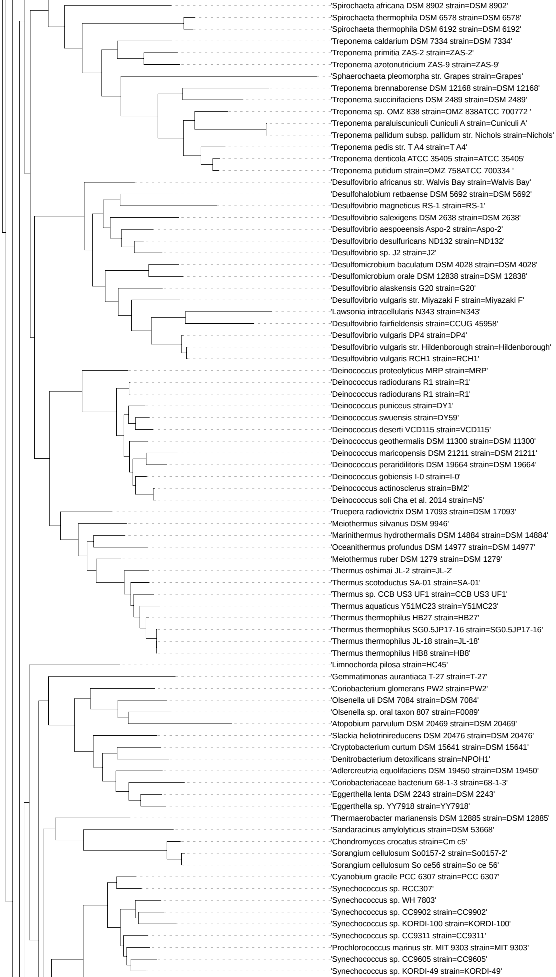
'Spirochaeta africana DSM 8902 strain=DSM 8902'
'Spirochaeta thermophila DSM 6578 strain=DSM 6578'
'Spirochaeta thermophila DSM 6192 strain=DSM 6192'
'Treponema caldarium DSM 7334 strain=DSM 7334'
'Treponema primitia ZAS-2 strain=ZAS-2'
'Treponema azotonutricium ZAS-9 strain=ZAS-9'
'Sphaerochaeta pleomorpha str. Grapes strain=Grapes'
'Treponema brennaborense DSM 12168 strain=DSM 12168'
'Treponema succinifaciens DSM 2489 strain=DSM 2489'
'Treponema sp. OMZ 838 strain=OMZ 838ATCC 700772 '
'Treponema paraluiscuniculi Cuniculi A strain=Cuniculi A'
'Treponema pallidum subsp. pallidum str. Nichols strain=Nichols'
'Treponema pedis str. T A4 strain=T A4'
'Treponema denticola ATCC 35405 strain=ATCC 35405'
'Treponema putidum strain=OMZ 758ATCC 700334 '
'Desulfovibrio africanus str. Walvis Bay strain=Walvis Bay'
'Desulfohalobium retbaense DSM 5692 strain=DSM 5692'
'Desulfovibrio magneticus RS-1 strain=RS-1'
'Desulfovibrio salexigens DSM 2638 strain=DSM 2638'
'Desulfovibrio aespoeensis Aspo-2 strain=Aspo-2'
'Desulfovibrio desulfuricans ND132 strain=ND132'
'Desulfovibrio sp. J2 strain=J2'
'Desulfomicrobium baculatum DSM 4028 strain=DSM 4028'
'Desulfomicrobium orale DSM 12838 strain=DSM 12838'
'Desulfovibrio alaskensis G20 strain=G20'
'Desulfovibrio vulgaris str. Miyazaki F strain=Miyazaki F'
'Lawsonia intracellularis N343 strain=N343'
'Desulfovibrio fairfieldensis strain=CCUG 45958'
'Desulfovibrio vulgaris DP4 strain=DP4'
'Desulfovibrio vulgaris str. Hildenborough strain=Hildenborough'
'Desulfovibrio vulgaris RCH1 strain=RCH1'
'Deinococcus proteolyticus MRP strain=MRP'
'Deinococcus radiodurans R1 strain=R1'
'Deinococcus radiodurans R1 strain=R1'
'Deinococcus puniceus strain=DY1'
'Deinococcus swuensis strain=DY59'
'Deinococcus deserti VCD115 strain=VCD115'
'Deinococcus geothermalis DSM 11300 strain=DSM 11300'
'Deinococcus maricopensis DSM 21211 strain=DSM 21211'
'Deinococcus peraridilitoris DSM 19664 strain=DSM 19664'
'Deinococcus gobiensis I-0 strain=I-0'
'Deinococcus actinosclerus strain=BM2'
'Deinococcus soli Cha et al. 2014 strain=N5'
'Truepera radiovictrix DSM 17093 strain=DSM 17093'
'Meiothermus silvanus DSM 9946'
'Marinithermus hydrothermalis DSM 14884 strain=DSM 14884'
'Oceanithermus profundus DSM 14977 strain=DSM 14977'
'Meiothermus ruber DSM 1279 strain=DSM 1279'
'Thermus oshimai JL-2 strain=JL-2'
'Thermus scotoductus SA-01 strain=SA-01'
'Thermus sp. CCB US3 UF1 strain=CCB US3 UF1'
'Thermus aquaticus Y51MC23 strain=Y51MC23'
'Thermus thermophilus HB27 strain=HB27'
'Thermus thermophilus SG0.5JP17-16 strain=SG0.5JP17-16'
'Thermus thermophilus JL-18 strain=JL-18'
'Thermus thermophilus HB8 strain=HB8'
'Limnochorda pilosa strain=HC45'
'Gemmatimonas aurantiaca T-27 strain=T-27'
'Coriobacterium glomerans PW2 strain=PW2'
'Olsenella uli DSM 7084 strain=DSM 7084'
'Olsenella sp. oral taxon 807 strain=F0089'
'Atopobium parvulum DSM 20469 strain=DSM 20469'
'Slackia heliotrinireducens DSM 20476 strain=DSM 20476'
'Cryptobacterium curtum DSM 15641 strain=DSM 15641'
'Denitrobacterium detoxificans strain=NPOH1'
'Adlercreutzia equolifaciens DSM 19450 strain=DSM 19450'
'Coriobacteriaceae bacterium 68-1-3 strain=68-1-3'
'Eggerthella lenta DSM 2243 strain=DSM 2243'
'Eggerthella sp. YY7918 strain=YY7918'
'Thermaerobacter marianensis DSM 12885 strain=DSM 12885'
'Sandaracinus amylolyticus strain=DSM 53668'
'Chondromyces crocatus strain=Cm c5'
'Sorangium cellulosum So0157-2 strain=So0157-2'
'Sorangium cellulosum So ce56 strain=So ce 56'
'Cyanobium gracile PCC 6307 strain=PCC 6307'
'Synechococcus sp. RCC307'
'Synechococcus sp. WH 7803'
'Synechococcus sp. CC9902 strain=CC9902'
'Synechococcus sp. KORDI-100 strain=KORDI-100'
'Synechococcus sp. CC9311 strain=CC9311'
'Prochlorococcus marinus str. MIT 9303 strain=MIT 9303'
'Synechococcus sp. CC9605 strain=CC9605'
'Synechococcus sp. KORDI-49 strain=KORDI-49'

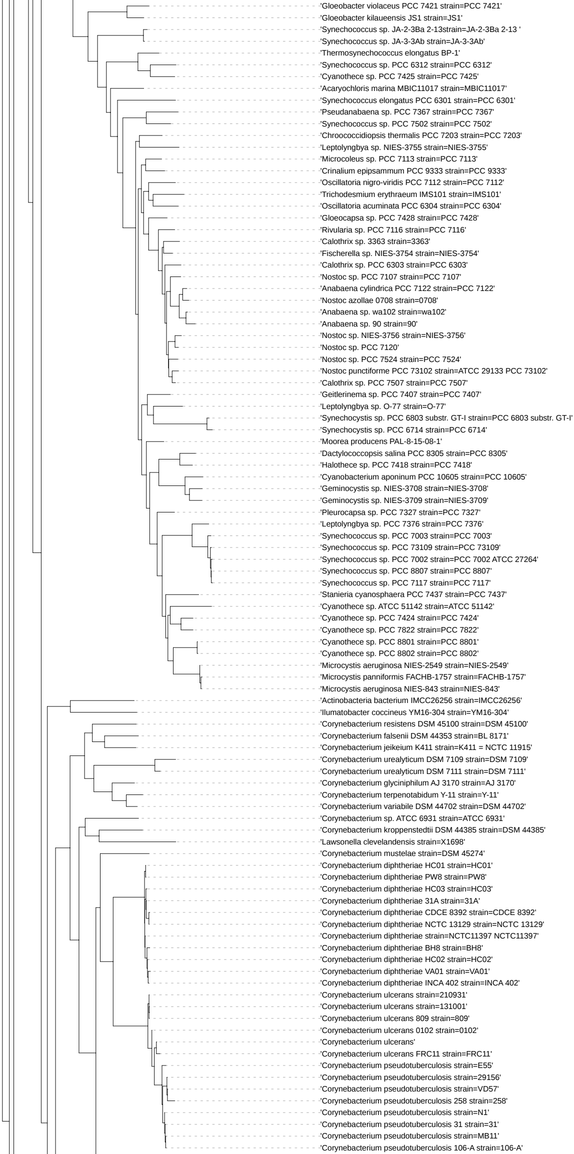
'Gloeobacter violaceus PCC 7421 strain=PCC 7421'
'Gloeobacter kilaueensis JS1 strain=JS1'
'Synechococcus sp. JA-2-3Ba 2-13strain=JA-2-3Ba 2-13 '
'Synechococcus sp. JA-3-3Ab strain=JA-3-3Ab'
'Thermosynechococcus elongatus BP-1'
'Synechococcus sp. PCC 6312 strain=PCC 6312'
'Cyanothece sp. PCC 7425 strain=PCC 7425'
'Acaryochloris marina MBIC11017 strain=MBIC11017'
'Synechococcus elongatus PCC 6301 strain=PCC 6301'
'Pseudanabaena sp. PCC 7367 strain=PCC 7367'
'Synechococcus sp. PCC 7502 strain=PCC 7502'
'Chroococcidiopsis thermalis PCC 7203 strain=PCC 7203'
'Leptolyngbya sp. NIES-3755 strain=NIES-3755'
'Microcoleus sp. PCC 7113 strain=PCC 7113'
'Crinalium epipsammum PCC 9333 strain=PCC 9333'
'Oscillatoria nigro-viridis PCC 7112 strain=PCC 7112'
'Trichodesmium erythraeum IMS101 strain=IMS101'
'Oscillatoria acuminata PCC 6304 strain=PCC 6304'
'Gloeocapsa sp. PCC 7428 strain=PCC 7428'
'Rivularia sp. PCC 7116 strain=PCC 7116'
'Calothrix sp. 3363 strain=3363'
'Fischerella sp. NIES-3754 strain=NIES-3754'
'Calothrix sp. PCC 6303 strain=PCC 6303'
'Nostoc sp. PCC 7107 strain=PCC 7107'
'Anabaena cylindrica PCC 7122 strain=PCC 7122'
'Nostoc azollae 0708 strain=0708'
'Anabaena sp. wa102 strain=wa102'
'Anabaena sp. 90 strain=90'
'Nostoc sp. NIES-3756 strain=NIES-3756'
'Nostoc sp. PCC 7120'
'Nostoc sp. PCC 7524 strain=PCC 7524'
'Nostoc punctiforme PCC 73102 strain=ATCC 29133 PCC 73102'
'Calothrix sp. PCC 7507 strain=PCC 7507'
'Geitlerinema sp. PCC 7407 strain=PCC 7407'
'Leptolyngbya sp. O-77 strain=O-77'
'Synechocystis sp. PCC 6803 substr. GT-I strain=PCC 6803 substr. GT-I'
'Synechocystis sp. PCC 6714 strain=PCC 6714'
'Moorea producens PAL-8-15-08-1'
'Dactylococcopsis salina PCC 8305 strain=PCC 8305'
'Halothece sp. PCC 7418 strain=PCC 7418'
'Cyanobacterium aponinum PCC 10605 strain=PCC 10605'
'Geminocystis sp. NIES-3708 strain=NIES-3708'
'Geminocystis sp. NIES-3709 strain=NIES-3709'
'Pleurocapsa sp. PCC 7327 strain=PCC 7327'
'Leptolyngbya sp. PCC 7376 strain=PCC 7376'
'Synechococcus sp. PCC 7003 strain=PCC 7003'
'Synechococcus sp. PCC 73109 strain=PCC 73109'
'Synechococcus sp. PCC 7002 strain=PCC 7002 ATCC 27264'
'Synechococcus sp. PCC 8807 strain=PCC 8807'
'Synechococcus sp. PCC 7117 strain=PCC 7117'
'Stanieria cyanosphaera PCC 7437 strain=PCC 7437'
'Cyanothece sp. ATCC 51142 strain=ATCC 51142'
'Cyanothece sp. PCC 7424 strain=PCC 7424'
'Cyanothece sp. PCC 7822 strain=PCC 7822'
'Cyanothece sp. PCC 8801 strain=PCC 8801'
'Cyanothece sp. PCC 8802 strain=PCC 8802'
'Microcystis aeruginosa NIES-2549 strain=NIES-2549'
'Microcystis panniformis FACHB-1757 strain=FACHB-1757'
'Microcystis aeruginosa NIES-843 strain=NIES-843'
'Actinobacteria bacterium IMCC26256 strain=IMCC26256'
'Ilumatobacter coccineus YM16-304 strain=YM16-304'
'Corynebacterium resistens DSM 45100 strain=DSM 45100'
'Corynebacterium falsenii DSM 44353 strain=BL 8171'
'Corynebacterium jeikeium K411 strain=K411 = NCTC 11915'
'Corynebacterium urealyticum DSM 7109 strain=DSM 7109'
'Corynebacterium urealyticum DSM 7111 strain=DSM 7111'
'Corynebacterium glyciniphilum AJ 3170 strain=AJ 3170'
'Corynebacterium terpenotabidum Y-11 strain=Y-11'
'Corynebacterium variabile DSM 44702 strain=DSM 44702'
'Corynebacterium sp. ATCC 6931 strain=ATCC 6931'
'Corynebacterium kroppenstedtii DSM 44385 strain=DSM 44385'
'Lawsonella clevelandensis strain=X1698'
'Corynebacterium mustelae strain=DSM 45274'
'Corynebacterium diphtheriae HC01 strain=HC01'
'Corynebacterium diphtheriae PW8 strain=PW8'
'Corynebacterium diphtheriae HC03 strain=HC03'
'Corynebacterium diphtheriae 31A strain=31A'
'Corynebacterium diphtheriae CDCE 8392 strain=CDCE 8392'
'Corynebacterium diphtheriae NCTC 13129 strain=NCTC 13129'
'Corynebacterium diphtheriae strain=NCTC11397 NCTC11397'
'Corynebacterium diphtheriae BH8 strain=BH8'
'Corynebacterium diphtheriae HC02 strain=HC02'
'Corynebacterium diphtheriae VA01 strain=VA01'
'Corynebacterium diphtheriae INCA 402 strain=INCA 402'
'Corynebacterium ulcerans strain=210931'
'Corynebacterium ulcerans strain=131001'
'Corynebacterium ulcerans 809 strain=809'
'Corynebacterium ulcerans 0102 strain=0102'
'Corynebacterium ulcerans'
'Corynebacterium ulcerans FRC11 strain=FRC11'
'Corynebacterium pseudotuberculosis strain=E55'
'Corynebacterium pseudotuberculosis strain=29156'
'Corynebacterium pseudotuberculosis strain=VD57'
'Corynebacterium pseudotuberculosis 258 strain=258'
'Corynebacterium pseudotuberculosis strain=N1'
'Corynebacterium pseudotuberculosis 31 strain=31'
'Corynebacterium pseudotuberculosis strain=MB11'
'Corynebacterium pseudotuberculosis 106-A strain=106-A'

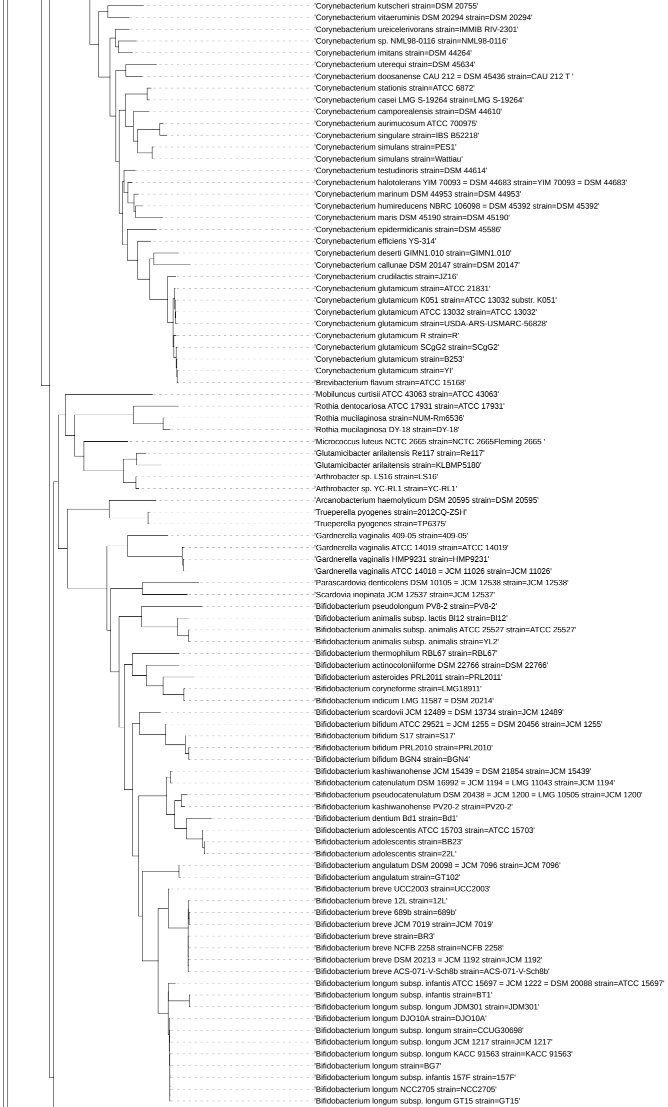
'Corynebacterium kutscheri strain=DSM 20755'
'Corynebacterium vitaeruminis DSM 20294 strain=DSM 20294'
'Corynebacterium ureicelerivorans strain=IMMIB RIV-2301'
'Corynebacterium sp. NML98-0116 strain=NML98-0116'
'Corynebacterium imitans strain=DSM 44264'
'Corynebacterium uterequi strain=DSM 45634'
'Corynebacterium doosanense CAU 212 = DSM 45436 strain=CAU 212 T '
'Corynebacterium stationis strain=ATCC 6872'
'Corynebacterium casei LMG S-19264 strain=LMG S-19264'
'Corynebacterium camporealensis strain=DSM 44610'
'Corynebacterium aurimucosum ATCC 700975'
'Corynebacterium singulare strain=IBS B52218'
'Corynebacterium simulans strain=PES1'
'Corynebacterium simulans strain=Wattiau'
'Corynebacterium testudinoris strain=DSM 44614'
'Corynebacterium halotolerans YIM 70093 = DSM 44683 strain=YIM 70093 = DSM 44683'
'Corynebacterium marinum DSM 44953 strain=DSM 44953'
'Corynebacterium humireducens NBRC 106098 = DSM 45392 strain=DSM 45392'
'Corynebacterium maris DSM 45190 strain=DSM 45190'
'Corynebacterium epidermidicanis strain=DSM 45586'
'Corynebacterium efficiens YS-314'
'Corynebacterium deserti GIMN1.010 strain=GIMN1.010'
'Corynebacterium callunae DSM 20147 strain=DSM 20147'
'Corynebacterium crudilactis strain=JZ16'
'Corynebacterium glutamicum strain=ATCC 21831'
'Corynebacterium glutamicum K051 strain=ATCC 13032 substr. K051'
'Corynebacterium glutamicum ATCC 13032 strain=ATCC 13032'
'Corynebacterium glutamicum strain=USDA-ARS-USMARC-56828'
'Corynebacterium glutamicum R strain=R'
'Corynebacterium glutamicum SCgG2 strain=SCgG2'
'Corynebacterium glutamicum strain=B253'
'Corynebacterium glutamicum strain=YI'
'Brevibacterium flavum strain=ATCC 15168'
'Mobiluncus curtisii ATCC 43063 strain=ATCC 43063'
'Rothia dentocariosa ATCC 17931 strain=ATCC 17931'
'Rothia mucilaginosa strain=NUM-Rm6536'
'Rothia mucilaginosa DY-18 strain=DY-18'
'Micrococcus luteus NCTC 2665 strain=NCTC 2665Fleming 2665 '
'Glutamicibacter arilaitensis Re117 strain=Re117'
'Glutamicibacter arilaitensis strain=KLBMP5180'
'Arthrobacter sp. LS16 strain=LS16'
'Arthrobacter sp. YC-RL1 strain=YC-RL1'
'Arcanobacterium haemolyticum DSM 20595 strain=DSM 20595'
'Trueperella pyogenes strain=2012CQ-ZSH'
'Trueperella pyogenes strain=TP6375'
'Gardnerella vaginalis 409-05 strain=409-05'
'Gardnerella vaginalis ATCC 14019 strain=ATCC 14019'
'Gardnerella vaginalis HMP9231 strain=HMP9231'
'Gardnerella vaginalis ATCC 14018 = JCM 11026 strain=JCM 11026'
'Parascardovia denticolens DSM 10105 = JCM 12538 strain=JCM 12538'
'Scardovia inopinata JCM 12537 strain=JCM 12537'
'Bifidobacterium pseudolongum PV8-2 strain=PV8-2'
'Bifidobacterium animalis subsp. lactis Bl12 strain=Bl12'
'Bifidobacterium animalis subsp. animalis ATCC 25527 strain=ATCC 25527'
'Bifidobacterium animalis subsp. animalis strain=YL2'
'Bifidobacterium thermophilum RBL67 strain=RBL67'
'Bifidobacterium actinocoloniiforme DSM 22766 strain=DSM 22766'
'Bifidobacterium asteroides PRL2011 strain=PRL2011'
'Bifidobacterium coryneforme strain=LMG18911'
'Bifidobacterium indicum LMG 11587 = DSM 20214'
'Bifidobacterium scardovii JCM 12489 = DSM 13734 strain=JCM 12489'
'Bifidobacterium bifidum ATCC 29521 = JCM 1255 = DSM 20456 strain=JCM 1255'
'Bifidobacterium bifidum S17 strain=S17'
'Bifidobacterium bifidum PRL2010 strain=PRL2010'
'Bifidobacterium bifidum BGN4 strain=BGN4'
'Bifidobacterium kashiwanohense JCM 15439 = DSM 21854 strain=JCM 15439'
'Bifidobacterium catenulatum DSM 16992 = JCM 1194 = LMG 11043 strain=JCM 1194'
'Bifidobacterium pseudocatenulatum DSM 20438 = JCM 1200 = LMG 10505 strain=JCM 1200'
'Bifidobacterium kashiwanohense PV20-2 strain=PV20-2'
'Bifidobacterium dentium Bd1 strain=Bd1'
'Bifidobacterium adolescentis ATCC 15703 strain=ATCC 15703'
'Bifidobacterium adolescentis strain=BB23'
'Bifidobacterium adolescentis strain=22L'
'Bifidobacterium angulatum DSM 20098 = JCM 7096 strain=JCM 7096'
'Bifidobacterium angulatum strain=GT102'
'Bifidobacterium breve UCC2003 strain=UCC2003'
'Bifidobacterium breve 12L strain=12L'
'Bifidobacterium breve 689b strain=689b'
'Bifidobacterium breve JCM 7019 strain=JCM 7019'
'Bifidobacterium breve strain=BR3'
'Bifidobacterium breve NCFB 2258 strain=NCFB 2258'
'Bifidobacterium breve DSM 20213 = JCM 1192 strain=JCM 1192'
'Bifidobacterium breve ACS-071-V-Sch8b strain=ACS-071-V-Sch8b'
'Bifidobacterium longum subsp. infantis ATCC 15697 = JCM 1222 = DSM 20088 strain=ATCC 15697'
'Bifidobacterium longum subsp. infantis strain=BT1'
'Bifidobacterium longum subsp. longum JDM301 strain=JDM301'
'Bifidobacterium longum DJO10A strain=DJO10A'
'Bifidobacterium longum subsp. longum strain=CCUG30698'
'Bifidobacterium longum subsp. longum JCM 1217 strain=JCM 1217'
'Bifidobacterium longum subsp. longum KACC 91563 strain=KACC 91563'
'Bifidobacterium longum strain=BG7'
'Bifidobacterium longum subsp. infantis 157F strain=157F'
'Bifidobacterium longum NCC2705 strain=NCC2705'
'Bifidobacterium longum subsp. longum GT15 strain=GT15'

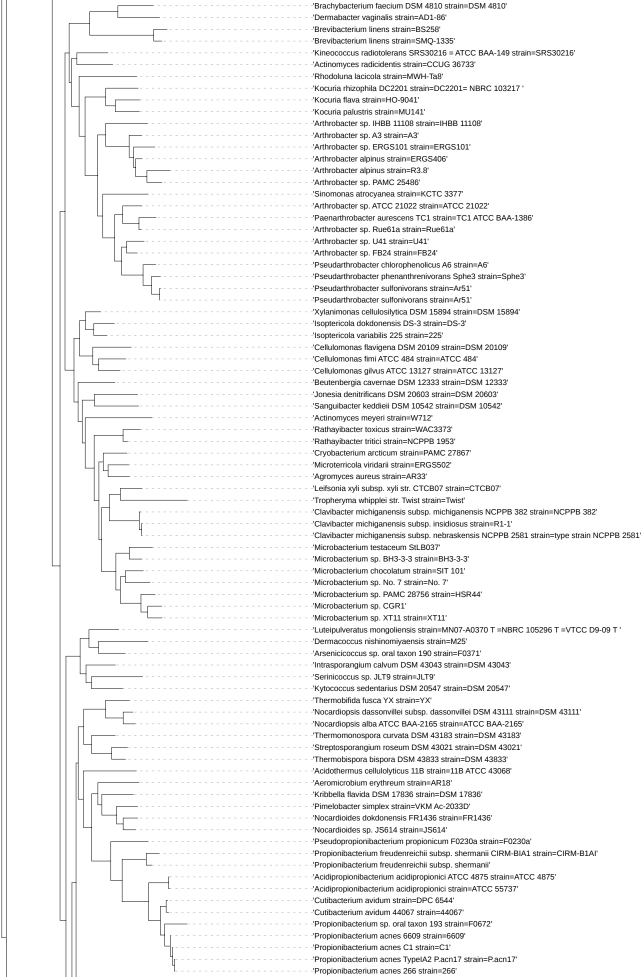
'Brachybacterium faecium DSM 4810 strain=DSM 4810'
'Dermabacter vaginalis strain=AD1-86'
'Brevibacterium linens strain=BS258'
'Brevibacterium linens strain=SMQ-1335'
'Kineococcus radiotolerans SRS30216 = ATCC BAA-149 strain=SRS30216'
'Actinomyces radicidentis strain=CCUG 36733'
'Rhodoluna lacicola strain=MWH-Ta8'
'Kocuria rhizophila DC2201 strain=DC2201= NBRC 103217 '
'Kocuria flava strain=HO-9041'
'Kocuria palustris strain=MU141'
'Arthrobacter sp. IHBB 11108 strain=IHBB 11108'
'Arthrobacter sp. A3 strain=A3'
'Arthrobacter sp. ERGS101 strain=ERGS101'
'Arthrobacter alpinus strain=ERGS406'
'Arthrobacter alpinus strain=R3.8'
'Arthrobacter sp. PAMC 25486'
'Sinomonas atrocyanea strain=KCTC 3377'
'Arthrobacter sp. ATCC 21022 strain=ATCC 21022'
'Paenarthrobacter aurescens TC1 strain=TC1 ATCC BAA-1386'
'Arthrobacter sp. Rue61a strain=Rue61a'
'Arthrobacter sp. U41 strain=U41'
'Arthrobacter sp. FB24 strain=FB24'
'Pseudarthrobacter chlorophenolicus A6 strain=A6'
'Pseudarthrobacter phenanthrenivorans Sphe3 strain=Sphe3'
'Pseudarthrobacter sulfonivorans strain=Ar51'
'Pseudarthrobacter sulfonivorans strain=Ar51'
'Xylanimonas cellulosilytica DSM 15894 strain=DSM 15894'
'Isoptericola dokdonensis DS-3 strain=DS-3'
'Isoptericola variabilis 225 strain=225'
'Cellulomonas flavigena DSM 20109 strain=DSM 20109'
'Cellulomonas fimi ATCC 484 strain=ATCC 484'
'Cellulomonas gilvus ATCC 13127 strain=ATCC 13127'
'Beutenbergia cavernae DSM 12333 strain=DSM 12333'
'Jonesia denitrificans DSM 20603 strain=DSM 20603'
'Sanguibacter keddieii DSM 10542 strain=DSM 10542'
'Actinomyces meyeri strain=W712'
'Rathayibacter toxicus strain=WAC3373'
'Rathayibacter tritici strain=NCPPB 1953'
'Cryobacterium arcticum strain=PAMC 27867'
'Microterricola viridarii strain=ERGS502'
'Agromyces aureus strain=AR33'
'Leifsonia xyli subsp. xyli str. CTCB07 strain=CTCB07'
'Tropheryma whipplei str. Twist strain=Twist'
'Clavibacter michiganensis subsp. michiganensis NCPPB 382 strain=NCPPB 382'
'Clavibacter michiganensis subsp. insidiosus strain=R1-1'
'Clavibacter michiganensis subsp. nebraskensis NCPPB 2581 strain=type strain NCPPB 2581'
'Microbacterium testaceum StLB037'
'Microbacterium sp. BH3-3-3 strain=BH3-3-3'
'Microbacterium chocolatum strain=SIT 101'
'Microbacterium sp. No. 7 strain=No. 7'
'Microbacterium sp. PAMC 28756 strain=HSR44'
'Microbacterium sp. CGR1'
'Microbacterium sp. XT11 strain=XT11'
'Luteipulveratus mongoliensis strain=MN07-A0370 T =NBRC 105296 T =VTCC D9-09 T '
'Dermacoccus nishinomiyaensis strain=M25'
'Arsenicicoccus sp. oral taxon 190 strain=F0371'
'Intrasporangium calvum DSM 43043 strain=DSM 43043'
'Serinicoccus sp. JLT9 strain=JLT9'
'Kytococcus sedentarius DSM 20547 strain=DSM 20547'
'Thermobifida fusca YX strain=YX'
'Nocardiopsis dassonvillei subsp. dassonvillei DSM 43111 strain=DSM 43111'
'Nocardiopsis alba ATCC BAA-2165 strain=ATCC BAA-2165'
'Thermomonospora curvata DSM 43183 strain=DSM 43183'
'Streptosporangium roseum DSM 43021 strain=DSM 43021'
'Thermobispora bispora DSM 43833 strain=DSM 43833'
'Acidothermus cellulolyticus 11B strain=11B ATCC 43068'
'Aeromicrobium erythreum strain=AR18'
'Kribbella flavida DSM 17836 strain=DSM 17836'
'Pimelobacter simplex strain=VKM Ac-2033D'
'Nocardioides dokdonensis FR1436 strain=FR1436'
'Nocardioides sp. JS614 strain=JS614'
'Pseudopropionibacterium propionicum F0230a strain=F0230a'
'Propionibacterium freudenreichii subsp. shermanii CIRM-BIA1 strain=CIRM-B1AI'
'Propionibacterium freudenreichii subsp. shermanii'
'Acidipropionibacterium acidipropionici ATCC 4875 strain=ATCC 4875'
'Acidipropionibacterium acidipropionici strain=ATCC 55737'
'Cutibacterium avidum strain=DPC 6544'
'Cutibacterium avidum 44067 strain=44067'
'Propionibacterium sp. oral taxon 193 strain=F0672'
'Propionibacterium acnes 6609 strain=6609'
'Propionibacterium acnes C1 strain=C1'
'Propionibacterium acnes TypeIA2 P.acn17 strain=P.acn17'
'Propionibacterium acnes 266 strain=266'

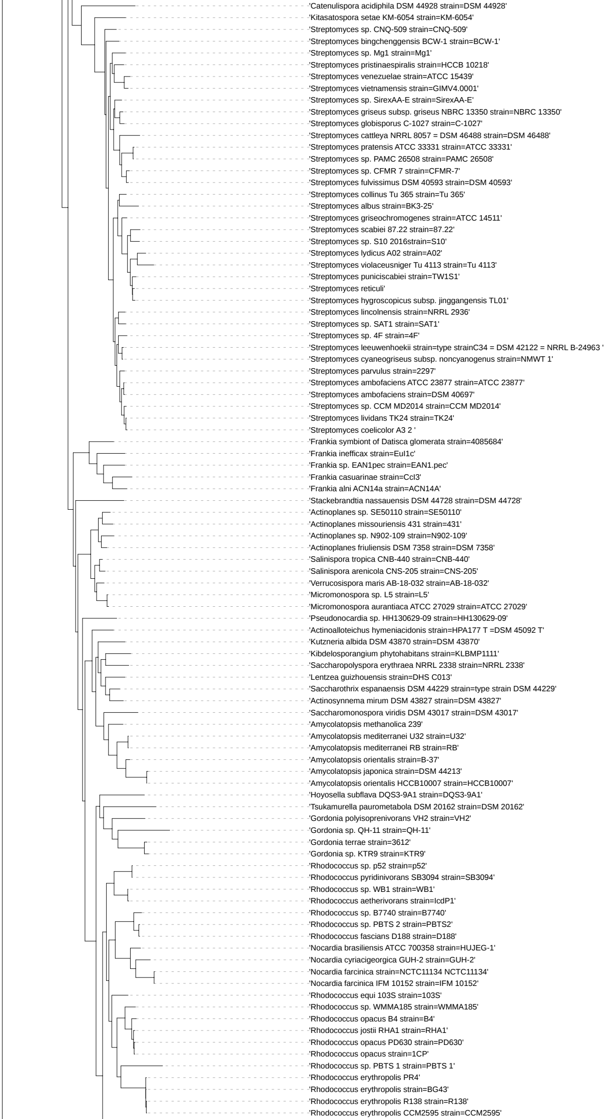
'Catenulispora acidiphila DSM 44928 strain=DSM 44928'
'Kitasatospora setae KM-6054 strain=KM-6054'
'Streptomyces sp. CNQ-509 strain=CNQ-509'
'Streptomyces bingchenggensis BCW-1 strain=BCW-1'
'Streptomyces sp. Mg1 strain=Mg1'
'Streptomyces pristinaespiralis strain=HCCB 10218'
'Streptomyces venezuelae strain=ATCC 15439'
'Streptomyces vietnamensis strain=GIMV4.0001'
'Streptomyces sp. SirexAA-E strain=SirexAA-E'
'Streptomyces griseus subsp. griseus NBRC 13350 strain=NBRC 13350'
'Streptomyces globisporus C-1027 strain=C-1027'
'Streptomyces cattleya NRRL 8057 = DSM 46488 strain=DSM 46488'
'Streptomyces pratensis ATCC 33331 strain=ATCC 33331'
'Streptomyces sp. PAMC 26508 strain=PAMC 26508'
'Streptomyces sp. CFMR 7 strain=CFMR-7'
'Streptomyces fulvissimus DSM 40593 strain=DSM 40593'
'Streptomyces collinus Tu 365 strain=Tu 365'
'Streptomyces albus strain=BK3-25'
'Streptomyces griseochromogenes strain=ATCC 14511'
'Streptomyces scabiei 87.22 strain=87.22'
'Streptomyces sp. S10 2016strain=S10'
'Streptomyces lydicus A02 strain=A02'
'Streptomyces violaceusniger Tu 4113 strain=Tu 4113'
'Streptomyces puniciscabiei strain=TW1S1'
'Streptomyces reticuli'
'Streptomyces hygroscopicus subsp. jinggangensis TL01'
'Streptomyces lincolnensis strain=NRRL 2936'
'Streptomyces sp. SAT1 strain=SAT1'
'Streptomyces sp. 4F strain=4F'
'Streptomyces leeuwenhoekii strain=type strainC34 = DSM 42122 = NRRL B-24963 '
'Streptomyces cyaneogriseus subsp. noncyanogenus strain=NMWT 1'
'Streptomyces parvulus strain=2297'
'Streptomyces ambofaciens ATCC 23877 strain=ATCC 23877'
'Streptomyces ambofaciens strain=DSM 40697'
'Streptomyces sp. CCM MD2014 strain=CCM MD2014'
'Streptomyces lividans TK24 strain=TK24'
'Streptomyces coelicolor A3 2 '
'Frankia symbiont of Datisca glomerata strain=4085684'
'Frankia inefficax strain=EuI1c'
'Frankia sp. EAN1pec strain=EAN1.pec'
'Frankia casuarinae strain=CcI3'
'Frankia alni ACN14a strain=ACN14A'
'Stackebrandtia nassauensis DSM 44728 strain=DSM 44728'
'Actinoplanes sp. SE50110 strain=SE50110'
'Actinoplanes missouriensis 431 strain=431'
'Actinoplanes sp. N902-109 strain=N902-109'
'Actinoplanes friuliensis DSM 7358 strain=DSM 7358'
'Salinispora tropica CNB-440 strain=CNB-440'
'Salinispora arenicola CNS-205 strain=CNS-205'
'Verrucosispora maris AB-18-032 strain=AB-18-032'
'Micromonospora sp. L5 strain=L5'
'Micromonospora aurantiaca ATCC 27029 strain=ATCC 27029'
'Pseudonocardia sp. HH130629-09 strain=HH130629-09'
'Actinoalloteichus hymeniacidonis strain=HPA177 T =DSM 45092 T'
'Kutzneria albida DSM 43870 strain=DSM 43870'
'Kibdelosporangium phytohabitans strain=KLBMP1111'
'Saccharopolyspora erythraea NRRL 2338 strain=NRRL 2338'
'Lentzea guizhouensis strain=DHS C013'
'Saccharothrix espanaensis DSM 44229 strain=type strain DSM 44229'
'Actinosynnema mirum DSM 43827 strain=DSM 43827'
'Saccharomonospora viridis DSM 43017 strain=DSM 43017'
'Amycolatopsis methanolica 239'
'Amycolatopsis mediterranei U32 strain=U32'
'Amycolatopsis mediterranei RB strain=RB'
'Amycolatopsis orientalis strain=B-37'
'Amycolatopsis japonica strain=DSM 44213'
'Amycolatopsis orientalis HCCB10007 strain=HCCB10007'
'Hoyosella subflava DQS3-9A1 strain=DQS3-9A1'
'Tsukamurella paurometabola DSM 20162 strain=DSM 20162'
'Gordonia polyisoprenivorans VH2 strain=VH2'
'Gordonia sp. QH-11 strain=QH-11'
'Gordonia terrae strain=3612'
'Gordonia sp. KTR9 strain=KTR9'
'Rhodococcus sp. p52 strain=p52'
'Rhodococcus pyridinivorans SB3094 strain=SB3094'
'Rhodococcus sp. WB1 strain=WB1'
'Rhodococcus aetherivorans strain=IcdP1'
'Rhodococcus sp. B7740 strain=B7740'
'Rhodococcus sp. PBTS 2 strain=PBTS2'
'Rhodococcus fascians D188 strain=D188'
'Nocardia brasiliensis ATCC 700358 strain=HUJEG-1'
'Nocardia cyriacigeorgica GUH-2 strain=GUH-2'
'Nocardia farcinica strain=NCTC11134 NCTC11134'
'Nocardia farcinica IFM 10152 strain=IFM 10152'
'Rhodococcus equi 103S strain=103S'
'Rhodococcus sp. WMMA185 strain=WMMA185'
'Rhodococcus opacus B4 strain=B4'
'Rhodococcus jostii RHA1 strain=RHA1'
'Rhodococcus opacus PD630 strain=PD630'
'Rhodococcus opacus strain=1CP'
'Rhodococcus sp. PBTS 1 strain=PBTS 1'
'Rhodococcus erythropolis PR4'
'Rhodococcus erythropolis strain=BG43'
'Rhodococcus erythropolis R138 strain=R138'
'Rhodococcus erythropolis CCM2595 strain=CCM2595'

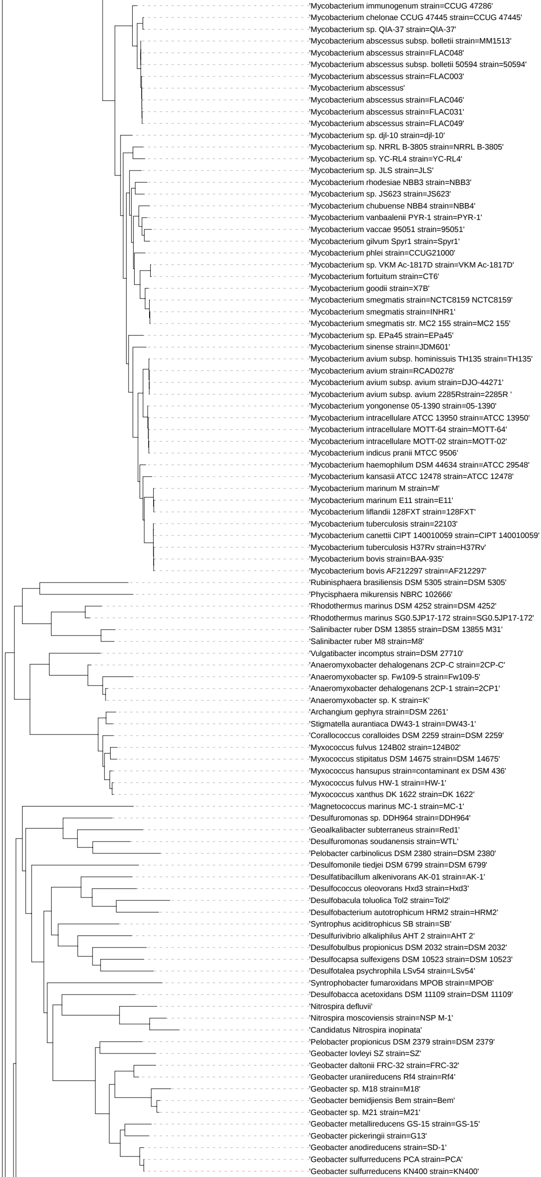
'Mycobacterium immunogenum strain=CCUG 47286'
'Mycobacterium chelonae CCUG 47445 strain=CCUG 47445'
'Mycobacterium sp. QIA-37 strain=QIA-37'
'Mycobacterium abscessus subsp. bolletii strain=MM1513'
'Mycobacterium abscessus strain=FLAC048'
'Mycobacterium abscessus subsp. bolletii 50594 strain=50594'
'Mycobacterium abscessus strain=FLAC003'
'Mycobacterium abscessus'
'Mycobacterium abscessus strain=FLAC046'
'Mycobacterium abscessus strain=FLAC031'
'Mycobacterium abscessus strain=FLAC049'
'Mycobacterium sp. djl-10 strain=djl-10'
'Mycobacterium sp. NRRL B-3805 strain=NRRL B-3805'
'Mycobacterium sp. YC-RL4 strain=YC-RL4'
'Mycobacterium sp. JLS strain=JLS'
'Mycobacterium rhodesiae NBB3 strain=NBB3'
'Mycobacterium sp. JS623 strain=JS623'
'Mycobacterium chubuense NBB4 strain=NBB4'
'Mycobacterium vanbaalenii PYR-1 strain=PYR-1'
'Mycobacterium vaccae 95051 strain=95051'
'Mycobacterium gilvum Spyr1 strain=Spyr1'
'Mycobacterium phlei strain=CCUG21000'
'Mycobacterium sp. VKM Ac-1817D strain=VKM Ac-1817D'
'Mycobacterium fortuitum strain=CT6'
'Mycobacterium goodii strain=X7B'
'Mycobacterium smegmatis strain=NCTC8159 NCTC8159'
'Mycobacterium smegmatis strain=INHR1'
'Mycobacterium smegmatis str. MC2 155 strain=MC2 155'
'Mycobacterium sp. EPa45 strain=EPa45'
'Mycobacterium sinense strain=JDM601'
'Mycobacterium avium subsp. hominissuis TH135 strain=TH135'
'Mycobacterium avium strain=RCAD0278'
'Mycobacterium avium subsp. avium strain=DJO-44271'
'Mycobacterium avium subsp. avium 2285Rstrain=2285R '
'Mycobacterium yongonense 05-1390 strain=05-1390'
'Mycobacterium intracellulare ATCC 13950 strain=ATCC 13950'
'Mycobacterium intracellulare MOTT-64 strain=MOTT-64'
'Mycobacterium intracellulare MOTT-02 strain=MOTT-02'
'Mycobacterium indicus pranii MTCC 9506'
'Mycobacterium haemophilum DSM 44634 strain=ATCC 29548'
'Mycobacterium kansasii ATCC 12478 strain=ATCC 12478'
'Mycobacterium marinum M strain=M'
'Mycobacterium marinum E11 strain=E11'
'Mycobacterium liflandii 128FXT strain=128FXT'
'Mycobacterium tuberculosis strain=22103'
'Mycobacterium canettii CIPT 140010059 strain=CIPT 140010059'
'Mycobacterium tuberculosis H37Rv strain=H37Rv'
'Mycobacterium bovis strain=BAA-935'
'Mycobacterium bovis AF212297 strain=AF212297'
'Rubinisphaera brasiliensis DSM 5305 strain=DSM 5305'
'Phycisphaera mikurensis NBRC 102666'
'Rhodothermus marinus DSM 4252 strain=DSM 4252'
'Rhodothermus marinus SG0.5JP17-172 strain=SG0.5JP17-172'
'Salinibacter ruber DSM 13855 strain=DSM 13855 M31'
'Salinibacter ruber M8 strain=M8'
'Vulgatibacter incomptus strain=DSM 27710'
'Anaeromyxobacter dehalogenans 2CP-C strain=2CP-C'
'Anaeromyxobacter sp. Fw109-5 strain=Fw109-5'
'Anaeromyxobacter dehalogenans 2CP-1 strain=2CP1'
'Anaeromyxobacter sp. K strain=K'
'Archangium gephyra strain=DSM 2261'
'Stigmatella aurantiaca DW43-1 strain=DW43-1'
'Corallococcus coralloides DSM 2259 strain=DSM 2259'
'Myxococcus fulvus 124B02 strain=124B02'
'Myxococcus stipitatus DSM 14675 strain=DSM 14675'
'Myxococcus hansupus strain=contaminant ex DSM 436'
'Myxococcus fulvus HW-1 strain=HW-1'
'Myxococcus xanthus DK 1622 strain=DK 1622'
'Magnetococcus marinus MC-1 strain=MC-1'
'Desulfuromonas sp. DDH964 strain=DDH964'
'Geoalkalibacter subterraneus strain=Red1'
'Desulfuromonas soudanensis strain=WTL'
'Pelobacter carbinolicus DSM 2380 strain=DSM 2380'
'Desulfomonile tiedjei DSM 6799 strain=DSM 6799'
'Desulfatibacillum alkenivorans AK-01 strain=AK-1'
'Desulfococcus oleovorans Hxd3 strain=Hxd3'
'Desulfobacula toluolica Tol2 strain=Tol2'
'Desulfobacterium autotrophicum HRM2 strain=HRM2'
'Syntrophus aciditrophicus SB strain=SB'
'Desulfurivibrio alkaliphilus AHT 2 strain=AHT 2'
'Desulfobulbus propionicus DSM 2032 strain=DSM 2032'
'Desulfocapsa sulfexigens DSM 10523 strain=DSM 10523'
'Desulfotalea psychrophila LSv54 strain=LSv54'
'Syntrophobacter fumaroxidans MPOB strain=MPOB'
'Desulfobacca acetoxidans DSM 11109 strain=DSM 11109'
'Nitrospira defluvii'
'Nitrospira moscoviensis strain=NSP M-1'
'Candidatus Nitrospira inopinata'
'Pelobacter propionicus DSM 2379 strain=DSM 2379'
'Geobacter lovleyi SZ strain=SZ'
'Geobacter daltonii FRC-32 strain=FRC-32'
'Geobacter uraniireducens Rf4 strain=Rf4'
'Geobacter sp. M18 strain=M18'
'Geobacter bemidjiensis Bem strain=Bem'
'Geobacter sp. M21 strain=M21'
'Geobacter metallireducens GS-15 strain=GS-15'
'Geobacter pickeringii strain=G13'
'Geobacter anodireducens strain=SD-1'
'Geobacter sulfurreducens PCA strain=PCA'
'Geobacter sulfurreducens KN400 strain=KN400'

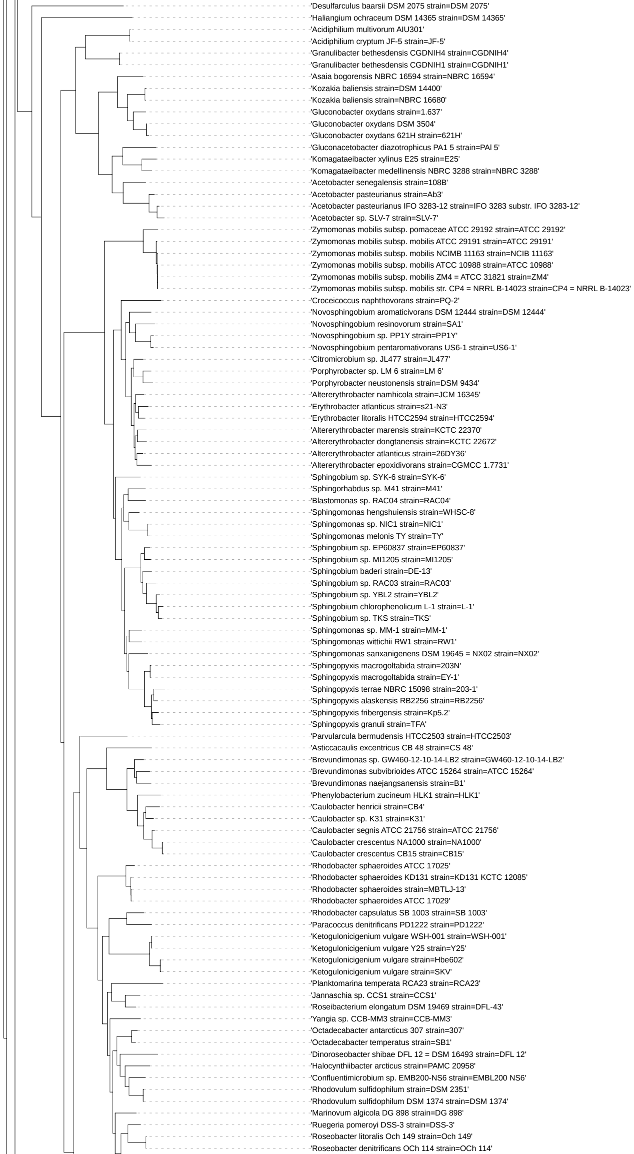
'Desulfarculus baarsii DSM 2075 strain=DSM 2075'
'Haliangium ochraceum DSM 14365 strain=DSM 14365'
'Acidiphilium multivorum AIU301'
'Acidiphilium cryptum JF-5 strain=JF-5'
'Granulibacter bethesdensis CGDNIH4 strain=CGDNIH4'
'Granulibacter bethesdensis CGDNIH1 strain=CGDNIH1'
'Asaia bogorensis NBRC 16594 strain=NBRC 16594'
'Kozakia baliensis strain=DSM 14400'
'Kozakia baliensis strain=NBRC 16680'
'Gluconobacter oxydans strain=1.637'
'Gluconobacter oxydans DSM 3504'
'Gluconobacter oxydans 621H strain=621H'
'Gluconacetobacter diazotrophicus PA1 5 strain=PAl 5'
'Komagataeibacter xylinus E25 strain=E25'
'Komagataeibacter medellinensis NBRC 3288 strain=NBRC 3288'
'Acetobacter senegalensis strain=108B'
'Acetobacter pasteurianus strain=Ab3'
'Acetobacter pasteurianus IFO 3283-12 strain=IFO 3283 substr. IFO 3283-12'
'Acetobacter sp. SLV-7 strain=SLV-7'
'Zymomonas mobilis subsp. pomaceae ATCC 29192 strain=ATCC 29192'
'Zymomonas mobilis subsp. mobilis ATCC 29191 strain=ATCC 29191'
'Zymomonas mobilis subsp. mobilis NCIMB 11163 strain=NCIB 11163'
'Zymomonas mobilis subsp. mobilis ATCC 10988 strain=ATCC 10988'
'Zymomonas mobilis subsp. mobilis ZM4 = ATCC 31821 strain=ZM4'
'Zymomonas mobilis subsp. mobilis str. CP4 = NRRL B-14023 strain=CP4 = NRRL B-14023'
'Croceicoccus naphthovorans strain=PQ-2'
'Novosphingobium aromaticivorans DSM 12444 strain=DSM 12444'
'Novosphingobium resinovorum strain=SA1'
'Novosphingobium sp. PP1Y strain=PP1Y'
'Novosphingobium pentaromativorans US6-1 strain=US6-1'
'Citromicrobium sp. JL477 strain=JL477'
'Porphyrobacter sp. LM 6 strain=LM 6'
'Porphyrobacter neustonensis strain=DSM 9434'
'Altererythrobacter namhicola strain=JCM 16345'
'Erythrobacter atlanticus strain=s21-N3'
'Erythrobacter litoralis HTCC2594 strain=HTCC2594'
'Altererythrobacter marensis strain=KCTC 22370'
'Altererythrobacter dongtanensis strain=KCTC 22672'
'Altererythrobacter atlanticus strain=26DY36'
'Altererythrobacter epoxidivorans strain=CGMCC 1.7731'
'Sphingobium sp. SYK-6 strain=SYK-6'
'Sphingorhabdus sp. M41 strain=M41'
'Blastomonas sp. RAC04 strain=RAC04'
'Sphingomonas hengshuiensis strain=WHSC-8'
'Sphingomonas sp. NIC1 strain=NIC1'
'Sphingomonas melonis TY strain=TY'
'Sphingobium sp. EP60837 strain=EP60837'
'Sphingobium sp. MI1205 strain=MI1205'
'Sphingobium baderi strain=DE-13'
'Sphingobium sp. RAC03 strain=RAC03'
'Sphingobium sp. YBL2 strain=YBL2'
'Sphingobium chlorophenolicum L-1 strain=L-1'
'Sphingobium sp. TKS strain=TKS'
'Sphingomonas sp. MM-1 strain=MM-1'
'Sphingomonas wittichii RW1 strain=RW1'
'Sphingomonas sanxanigenens DSM 19645 = NX02 strain=NX02'
'Sphingopyxis macrogoltabida strain=203N'
'Sphingopyxis macrogoltabida strain=EY-1'
'Sphingopyxis terrae NBRC 15098 strain=203-1'
'Sphingopyxis alaskensis RB2256 strain=RB2256'
'Sphingopyxis fribergensis strain=Kp5.2'
'Sphingopyxis granuli strain=TFA'
'Parvularcula bermudensis HTCC2503 strain=HTCC2503'
'Asticcacaulis excentricus CB 48 strain=CS 48'
'Brevundimonas sp. GW460-12-10-14-LB2 strain=GW460-12-10-14-LB2'
'Brevundimonas subvibrioides ATCC 15264 strain=ATCC 15264'
'Brevundimonas naejangsanensis strain=B1'
'Phenylobacterium zucineum HLK1 strain=HLK1'
'Caulobacter henricii strain=CB4'
'Caulobacter sp. K31 strain=K31'
'Caulobacter segnis ATCC 21756 strain=ATCC 21756'
'Caulobacter crescentus NA1000 strain=NA1000'
'Caulobacter crescentus CB15 strain=CB15'
'Rhodobacter sphaeroides ATCC 17025'
'Rhodobacter sphaeroides KD131 strain=KD131 KCTC 12085'
'Rhodobacter sphaeroides strain=MBTLJ-13'
'Rhodobacter sphaeroides ATCC 17029'
'Rhodobacter capsulatus SB 1003 strain=SB 1003'
'Paracoccus denitrificans PD1222 strain=PD1222'
'Ketogulonicigenium vulgare WSH-001 strain=WSH-001'
'Ketogulonicigenium vulgare Y25 strain=Y25'
'Ketogulonicigenium vulgare strain=Hbe602'
'Ketogulonicigenium vulgare strain=SKV'
'Planktomarina temperata RCA23 strain=RCA23'
'Jannaschia sp. CCS1 strain=CCS1'
'Roseibacterium elongatum DSM 19469 strain=DFL-43'
'Yangia sp. CCB-MM3 strain=CCB-MM3'
'Octadecabacter antarcticus 307 strain=307'
'Octadecabacter temperatus strain=SB1'
'Dinoroseobacter shibae DFL 12 = DSM 16493 strain=DFL 12'
'Halocynthiibacter arcticus strain=PAMC 20958'
'Confluentimicrobium sp. EMB200-NS6 strain=EMBL200 NS6'
'Rhodovulum sulfidophilum strain=DSM 2351'
'Rhodovulum sulfidophilum DSM 1374 strain=DSM 1374'
'Marinovum algicola DG 898 strain=DG 898'
'Ruegeria pomeroyi DSS-3 strain=DSS-3'
'Roseobacter litoralis Och 149 strain=Och 149'
'Roseobacter denitrificans OCh 114 strain=OCh 114'

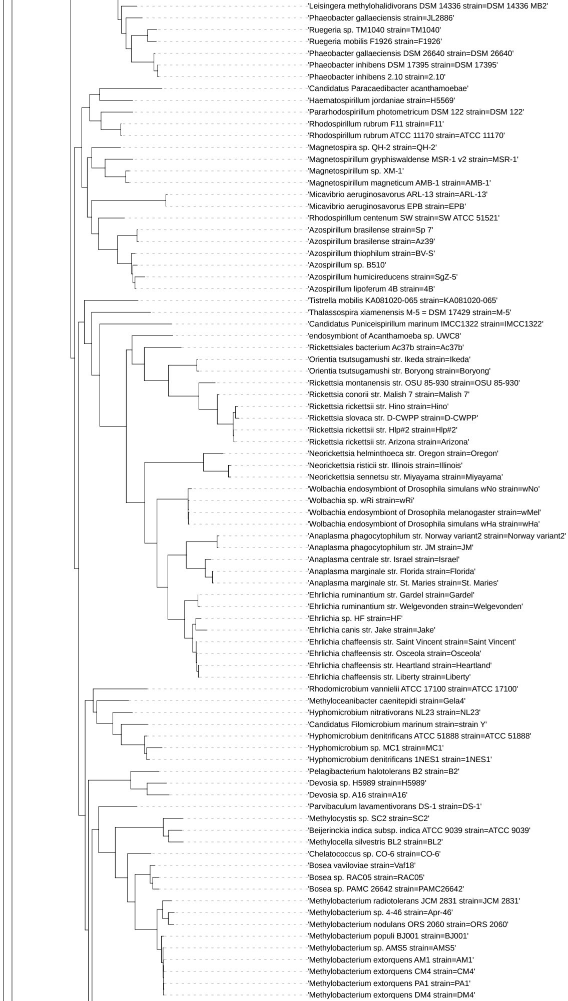
'Leisingera methylohalidivorans DSM 14336 strain=DSM 14336 MB2'
'Phaeobacter gallaeciensis strain=JL2886'
'Ruegeria sp. TM1040 strain=TM1040'
'Ruegeria mobilis F1926 strain=F1926'
'Phaeobacter gallaeciensis DSM 26640 strain=DSM 26640'
'Phaeobacter inhibens DSM 17395 strain=DSM 17395'
'Phaeobacter inhibens 2.10 strain=2.10'
'Candidatus Paracaedibacter acanthamoebae'
'Haematospirillum jordaniae strain=H5569'
'Pararhodospirillum photometricum DSM 122 strain=DSM 122'
'Rhodospirillum rubrum F11 strain=F11'
'Rhodospirillum rubrum ATCC 11170 strain=ATCC 11170'
'Magnetospira sp. QH-2 strain=QH-2'
'Magnetospirillum gryphiswaldense MSR-1 v2 strain=MSR-1'
'Magnetospirillum sp. XM-1'
'Magnetospirillum magneticum AMB-1 strain=AMB-1'
'Micavibrio aeruginosavorus ARL-13 strain=ARL-13'
'Micavibrio aeruginosavorus EPB strain=EPB'
'Rhodospirillum centenum SW strain=SW ATCC 51521'
'Azospirillum brasilense strain=Sp 7'
'Azospirillum brasilense strain=Az39'
'Azospirillum thiophilum strain=BV-S'
'Azospirillum sp. B510'
'Azospirillum humicireducens strain=SgZ-5'
'Azospirillum lipoferum 4B strain=4B'
'Tistrella mobilis KA081020-065 strain=KA081020-065'
'Thalassospira xiamenensis M-5 = DSM 17429 strain=M-5'
'Candidatus Puniceispirillum marinum IMCC1322 strain=IMCC1322'
'endosymbiont of Acanthamoeba sp. UWC8'
'Rickettsiales bacterium Ac37b strain=Ac37b'
'Orientia tsutsugamushi str. Ikeda strain=Ikeda'
'Orientia tsutsugamushi str. Boryong strain=Boryong'
'Rickettsia montanensis str. OSU 85-930 strain=OSU 85-930'
'Rickettsia conorii str. Malish 7 strain=Malish 7'
'Rickettsia rickettsii str. Hino strain=Hino'
'Rickettsia slovaca str. D-CWPP strain=D-CWPP'
'Rickettsia rickettsii str. Hlp#2 strain=Hlp#2'
'Rickettsia rickettsii str. Arizona strain=Arizona'
'Neorickettsia helminthoeca str. Oregon strain=Oregon'
'Neorickettsia risticii str. Illinois strain=Illinois'
'Neorickettsia sennetsu str. Miyayama strain=Miyayama'
'Wolbachia endosymbiont of Drosophila simulans wNo strain=wNo'
'Wolbachia sp. wRi strain=wRi'
'Wolbachia endosymbiont of Drosophila melanogaster strain=wMel'
'Wolbachia endosymbiont of Drosophila simulans wHa strain=wHa'
'Anaplasma phagocytophilum str. Norway variant2 strain=Norway variant2'
'Anaplasma phagocytophilum str. JM strain=JM'
'Anaplasma centrale str. Israel strain=Israel'
'Anaplasma marginale str. Florida strain=Florida'
'Anaplasma marginale str. St. Maries strain=St. Maries'
'Ehrlichia ruminantium str. Gardel strain=Gardel'
'Ehrlichia ruminantium str. Welgevonden strain=Welgevonden'
'Ehrlichia sp. HF strain=HF'
'Ehrlichia canis str. Jake strain=Jake'
'Ehrlichia chaffeensis str. Saint Vincent strain=Saint Vincent'
'Ehrlichia chaffeensis str. Osceola strain=Osceola'
'Ehrlichia chaffeensis str. Heartland strain=Heartland'
'Ehrlichia chaffeensis str. Liberty strain=Liberty'
'Rhodomicrobium vannielii ATCC 17100 strain=ATCC 17100'
'Methyloceanibacter caenitepidi strain=Gela4'
'Hyphomicrobium nitrativorans NL23 strain=NL23'
'Candidatus Filomicrobium marinum strain=strain Y'
'Hyphomicrobium denitrificans ATCC 51888 strain=ATCC 51888'
'Hyphomicrobium sp. MC1 strain=MC1'
'Hyphomicrobium denitrificans 1NES1 strain=1NES1'
'Pelagibacterium halotolerans B2 strain=B2'
'Devosia sp. H5989 strain=H5989'
'Devosia sp. A16 strain=A16'
'Parvibaculum lavamentivorans DS-1 strain=DS-1'
'Methylocystis sp. SC2 strain=SC2'
'Beijerinckia indica subsp. indica ATCC 9039 strain=ATCC 9039'
'Methylocella silvestris BL2 strain=BL2'
'Chelatococcus sp. CO-6 strain=CO-6'
'Bosea vaviloviae strain=Vaf18'
'Bosea sp. RAC05 strain=RAC05'
'Bosea sp. PAMC 26642 strain=PAMC26642'
'Methylobacterium radiotolerans JCM 2831 strain=JCM 2831'
'Methylobacterium sp. 4-46 strain=Apr-46'
'Methylobacterium nodulans ORS 2060 strain=ORS 2060'
'Methylobacterium populi BJ001 strain=BJ001'
'Methylobacterium sp. AMS5 strain=AMS5'
'Methylobacterium extorquens AM1 strain=AM1'
'Methylobacterium extorquens CM4 strain=CM4'
'Methylobacterium extorquens PA1 strain=PA1'
'Methylobacterium extorquens DM4 strain=DM4'

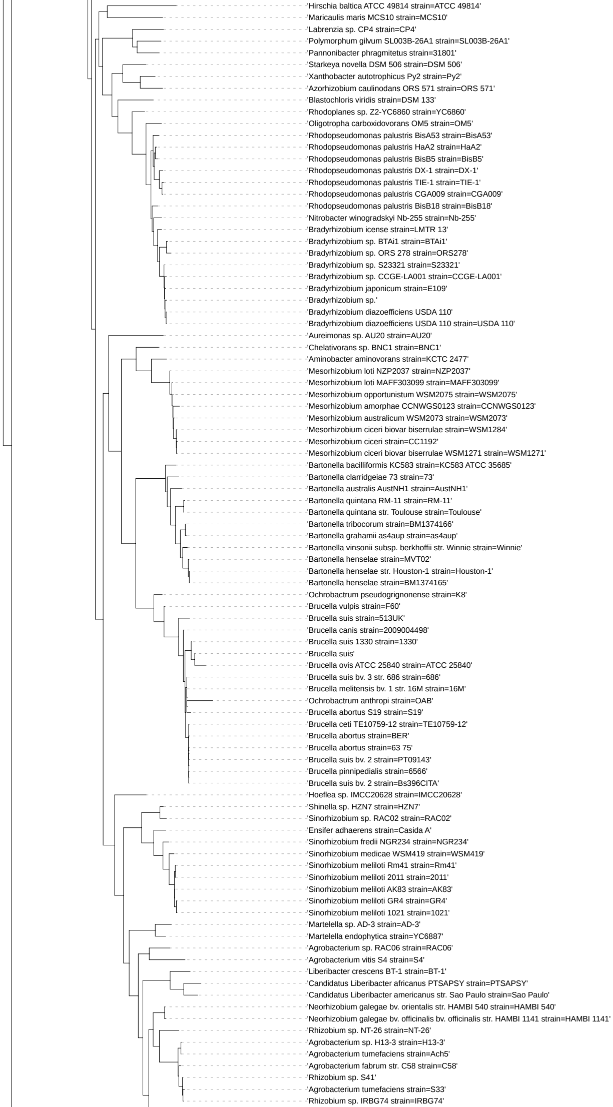
'Hirschia baltica ATCC 49814 strain=ATCC 49814'
'Maricaulis maris MCS10 strain=MCS10'
'Labrenzia sp. CP4 strain=CP4'
'Polymorphum gilvum SL003B-26A1 strain=SL003B-26A1'
'Pannonibacter phragmitetus strain=31801'
'Starkeya novella DSM 506 strain=DSM 506'
'Xanthobacter autotrophicus Py2 strain=Py2'
'Azorhizobium caulinodans ORS 571 strain=ORS 571'
'Blastochloris viridis strain=DSM 133'
'Rhodoplanes sp. Z2-YC6860 strain=YC6860'
'Oligotropha carboxidovorans OM5 strain=OM5'
'Rhodopseudomonas palustris BisA53 strain=BisA53'
'Rhodopseudomonas palustris HaA2 strain=HaA2'
'Rhodopseudomonas palustris BisB5 strain=BisB5'
'Rhodopseudomonas palustris DX-1 strain=DX-1'
'Rhodopseudomonas palustris TIE-1 strain=TIE-1'
'Rhodopseudomonas palustris CGA009 strain=CGA009'
'Rhodopseudomonas palustris BisB18 strain=BisB18'
'Nitrobacter winogradskyi Nb-255 strain=Nb-255'
'Bradyrhizobium icense strain=LMTR 13'
'Bradyrhizobium sp. BTAi1 strain=BTAi1'
'Bradyrhizobium sp. ORS 278 strain=ORS278'
'Bradyrhizobium sp. S23321 strain=S23321'
'Bradyrhizobium sp. CCGE-LA001 strain=CCGE-LA001'
'Bradyrhizobium japonicum strain=E109'
'Bradyrhizobium sp.'
'Bradyrhizobium diazoefficiens USDA 110'
'Bradyrhizobium diazoefficiens USDA 110 strain=USDA 110'
'Aureimonas sp. AU20 strain=AU20'
'Chelativorans sp. BNC1 strain=BNC1'
'Aminobacter aminovorans strain=KCTC 2477'
'Mesorhizobium loti NZP2037 strain=NZP2037'
'Mesorhizobium loti MAFF303099 strain=MAFF303099'
'Mesorhizobium opportunistum WSM2075 strain=WSM2075'
'Mesorhizobium amorphae CCNWGS0123 strain=CCNWGS0123'
'Mesorhizobium australicum WSM2073 strain=WSM2073'
'Mesorhizobium ciceri biovar biserrulae strain=WSM1284'
'Mesorhizobium ciceri strain=CC1192'
'Mesorhizobium ciceri biovar biserrulae WSM1271 strain=WSM1271'
'Bartonella bacilliformis KC583 strain=KC583 ATCC 35685'
'Bartonella clarridgeiae 73 strain=73'
'Bartonella australis AustNH1 strain=AustNH1'
'Bartonella quintana RM-11 strain=RM-11'
'Bartonella quintana str. Toulouse strain=Toulouse'
'Bartonella tribocorum strain=BM1374166'
'Bartonella grahamii as4aup strain=as4aup'
'Bartonella vinsonii subsp. berkhoffii str. Winnie strain=Winnie'
'Bartonella henselae strain=MVT02'
'Bartonella henselae str. Houston-1 strain=Houston-1'
'Bartonella henselae strain=BM1374165'
'Ochrobactrum pseudogrignonense strain=K8'
'Brucella vulpis strain=F60'
'Brucella suis strain=513UK'
'Brucella canis strain=2009004498'
'Brucella suis 1330 strain=1330'
'Brucella suis'
'Brucella ovis ATCC 25840 strain=ATCC 25840'
'Brucella suis bv. 3 str. 686 strain=686'
'Brucella melitensis bv. 1 str. 16M strain=16M'
'Ochrobactrum anthropi strain=OAB'
'Brucella abortus S19 strain=S19'
'Brucella ceti TE10759-12 strain=TE10759-12'
'Brucella abortus strain=BER'
'Brucella abortus strain=63 75'
'Brucella suis bv. 2 strain=PT09143'
'Brucella pinnipedialis strain=6566'
'Brucella suis bv. 2 strain=Bs396CITA'
'Hoeflea sp. IMCC20628 strain=IMCC20628'
'Shinella sp. HZN7 strain=HZN7'
'Sinorhizobium sp. RAC02 strain=RAC02'
'Ensifer adhaerens strain=Casida A'
'Sinorhizobium fredii NGR234 strain=NGR234'
'Sinorhizobium medicae WSM419 strain=WSM419'
'Sinorhizobium meliloti Rm41 strain=Rm41'
'Sinorhizobium meliloti 2011 strain=2011'
'Sinorhizobium meliloti AK83 strain=AK83'
'Sinorhizobium meliloti GR4 strain=GR4'
'Sinorhizobium meliloti 1021 strain=1021'
'Martelella sp. AD-3 strain=AD-3'
'Martelella endophytica strain=YC6887'
'Agrobacterium sp. RAC06 strain=RAC06'
'Agrobacterium vitis S4 strain=S4'
'Liberibacter crescens BT-1 strain=BT-1'
'Candidatus Liberibacter africanus PTSAPSY strain=PTSAPSY'
'Candidatus Liberibacter americanus str. Sao Paulo strain=Sao Paulo'
'Neorhizobium galegae bv. orientalis str. HAMBI 540 strain=HAMBI 540'
'Neorhizobium galegae bv. officinalis bv. officinalis str. HAMBI 1141 strain=HAMBI 1141'
'Rhizobium sp. NT-26 strain=NT-26'
'Agrobacterium sp. H13-3 strain=H13-3'
'Agrobacterium tumefaciens strain=Ach5'
'Agrobacterium fabrum str. C58 strain=C58'
'Rhizobium sp. S41'
'Agrobacterium tumefaciens strain=S33'
'Rhizobium sp. IRBG74 strain=IRBG74'

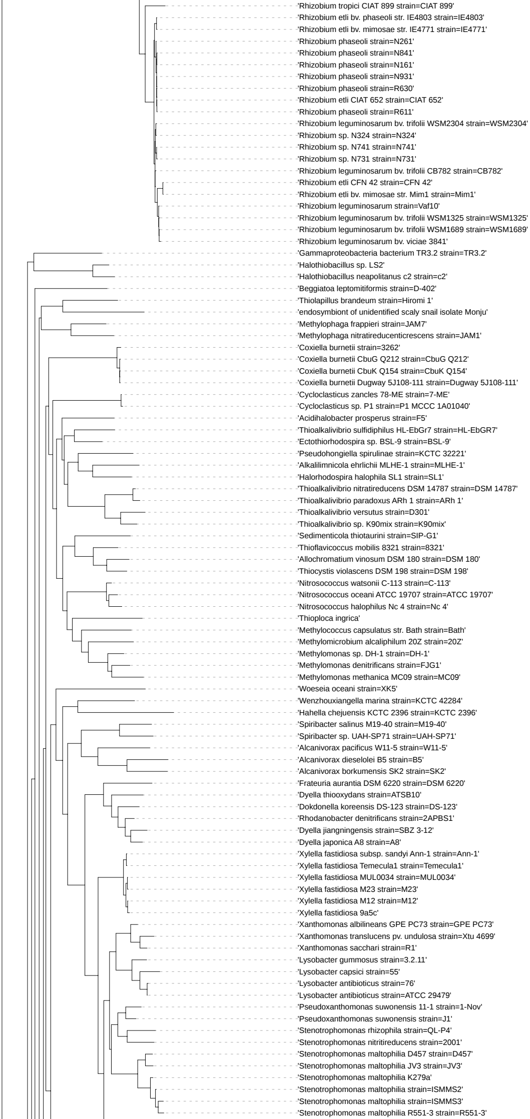
'Rhizobium tropici CIAT 899 strain=CIAT 899'
'Rhizobium etli bv. phaseoli str. IE4803 strain=IE4803'
'Rhizobium etli bv. mimosae str. IE4771 strain=IE4771'
'Rhizobium phaseoli strain=N261'
'Rhizobium phaseoli strain=N841'
'Rhizobium phaseoli strain=N161'
'Rhizobium phaseoli strain=N931'
'Rhizobium phaseoli strain=R630'
'Rhizobium etli CIAT 652 strain=CIAT 652'
'Rhizobium phaseoli strain=R611'
'Rhizobium leguminosarum bv. trifolii WSM2304 strain=WSM2304'
'Rhizobium sp. N324 strain=N324'
'Rhizobium sp. N741 strain=N741'
'Rhizobium sp. N731 strain=N731'
'Rhizobium leguminosarum bv. trifolii CB782 strain=CB782'
'Rhizobium etli CFN 42 strain=CFN 42'
'Rhizobium etli bv. mimosae str. Mim1 strain=Mim1'
'Rhizobium leguminosarum strain=Vaf10'
'Rhizobium leguminosarum bv. trifolii WSM1325 strain=WSM1325'
'Rhizobium leguminosarum bv. trifolii WSM1689 strain=WSM1689'
'Rhizobium leguminosarum bv. viciae 3841'
'Gammaproteobacteria bacterium TR3.2 strain=TR3.2'
'Halothiobacillus sp. LS2'
'Halothiobacillus neapolitanus c2 strain=c2'
'Beggiatoa leptomitiformis strain=D-402'
'Thiolapillus brandeum strain=Hiromi 1'
'endosymbiont of unidentified scaly snail isolate Monju'
'Methylophaga frappieri strain=JAM7'
'Methylophaga nitratireducenticrescens strain=JAM1'
'Coxiella burnetii strain=3262'
'Coxiella burnetii CbuG Q212 strain=CbuG Q212'
'Coxiella burnetii CbuK Q154 strain=CbuK Q154'
'Coxiella burnetii Dugway 5J108-111 strain=Dugway 5J108-111'
'Cycloclasticus zancles 78-ME strain=7-ME'
'Cycloclasticus sp. P1 strain=P1 MCCC 1A01040'
'Acidihalobacter prosperus strain=F5'
'Thioalkalivibrio sulfidiphilus HL-EbGr7 strain=HL-EbGR7'
'Ectothiorhodospira sp. BSL-9 strain=BSL-9'
'Pseudohongiella spirulinae strain=KCTC 32221'
'Alkalilimnicola ehrlichii MLHE-1 strain=MLHE-1'
'Halorhodospira halophila SL1 strain=SL1'
'Thioalkalivibrio nitratireducens DSM 14787 strain=DSM 14787'
'Thioalkalivibrio paradoxus ARh 1 strain=ARh 1'
'Thioalkalivibrio versutus strain=D301'
'Thioalkalivibrio sp. K90mix strain=K90mix'
'Sedimenticola thiotaurini strain=SIP-G1'
'Thioflavicoccus mobilis 8321 strain=8321'
'Allochromatium vinosum DSM 180 strain=DSM 180'
'Thiocystis violascens DSM 198 strain=DSM 198'
'Nitrosococcus watsonii C-113 strain=C-113'
'Nitrosococcus oceani ATCC 19707 strain=ATCC 19707'
'Nitrosococcus halophilus Nc 4 strain=Nc 4'
'Thioploca ingrica'
'Methylococcus capsulatus str. Bath strain=Bath'
'Methylomicrobium alcaliphilum 20Z strain=20Z'
'Methylomonas sp. DH-1 strain=DH-1'
'Methylomonas denitrificans strain=FJG1'
'Methylomonas methanica MC09 strain=MC09'
'Woeseia oceani strain=XK5'
'Wenzhouxiangella marina strain=KCTC 42284'
'Hahella chejuensis KCTC 2396 strain=KCTC 2396'
'Spiribacter salinus M19-40 strain=M19-40'
'Spiribacter sp. UAH-SP71 strain=UAH-SP71'
'Alcanivorax pacificus W11-5 strain=W11-5'
'Alcanivorax dieselolei B5 strain=B5'
'Alcanivorax borkumensis SK2 strain=SK2'
'Frateuria aurantia DSM 6220 strain=DSM 6220'
'Dyella thiooxydans strain=ATSB10'
'Dokdonella koreensis DS-123 strain=DS-123'
'Rhodanobacter denitrificans strain=2APBS1'
'Dyella jiangningensis strain=SBZ 3-12'
'Dyella japonica A8 strain=A8'
'Xylella fastidiosa subsp. sandyi Ann-1 strain=Ann-1'
'Xylella fastidiosa Temecula1 strain=Temecula1'
'Xylella fastidiosa MUL0034 strain=MUL0034'
'Xylella fastidiosa M23 strain=M23'
'Xylella fastidiosa M12 strain=M12'
'Xylella fastidiosa 9a5c'
'Xanthomonas albilineans GPE PC73 strain=GPE PC73'
'Xanthomonas translucens pv. undulosa strain=Xtu 4699'
'Xanthomonas sacchari strain=R1'
'Lysobacter gummosus strain=3.2.11'
'Lysobacter capsici strain=55'
'Lysobacter antibioticus strain=76'
'Lysobacter antibioticus strain=ATCC 29479'
'Pseudoxanthomonas suwonensis 11-1 strain=1-Nov'
'Pseudoxanthomonas suwonensis strain=J1'
'Stenotrophomonas rhizophila strain=QL-P4'
'Stenotrophomonas nitritireducens strain=2001'
'Stenotrophomonas maltophilia D457 strain=D457'
'Stenotrophomonas maltophilia JV3 strain=JV3'
'Stenotrophomonas maltophilia K279a'
'Stenotrophomonas maltophilia strain=ISMMS2'
'Stenotrophomonas maltophilia strain=ISMMS3'
'Stenotrophomonas maltophilia R551-3 strain=R551-3'

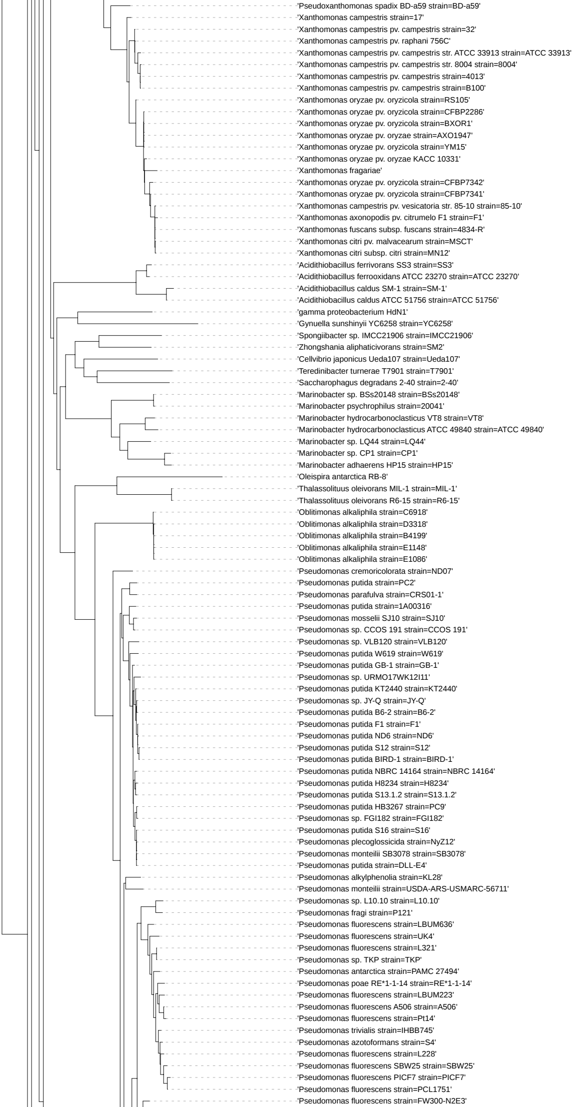
'Pseudoxanthomonas spadix BD-a59 strain=BD-a59'
'Xanthomonas campestris strain=17'
'Xanthomonas campestris pv. campestris strain=32'
'Xanthomonas campestris pv. raphani 756C'
'Xanthomonas campestris pv. campestris str. ATCC 33913 strain=ATCC 33913'
'Xanthomonas campestris pv. campestris str. 8004 strain=8004'
'Xanthomonas campestris pv. campestris strain=4013'
'Xanthomonas campestris pv. campestris strain=B100'
'Xanthomonas oryzae pv. oryzicola strain=RS105'
'Xanthomonas oryzae pv. oryzicola strain=CFBP2286'
'Xanthomonas oryzae pv. oryzicola strain=BXOR1'
'Xanthomonas oryzae pv. oryzae strain=AXO1947'
'Xanthomonas oryzae pv. oryzicola strain=YM15'
'Xanthomonas oryzae pv. oryzae KACC 10331'
'Xanthomonas fragariae'
'Xanthomonas oryzae pv. oryzicola strain=CFBP7342'
'Xanthomonas oryzae pv. oryzicola strain=CFBP7341'
'Xanthomonas campestris pv. vesicatoria str. 85-10 strain=85-10'
'Xanthomonas axonopodis pv. citrumelo F1 strain=F1'
'Xanthomonas fuscans subsp. fuscans strain=4834-R'
'Xanthomonas citri pv. malvacearum strain=MSCT'
'Xanthomonas citri subsp. citri strain=MN12'
'Acidithiobacillus ferrivorans SS3 strain=SS3'
'Acidithiobacillus ferrooxidans ATCC 23270 strain=ATCC 23270'
'Acidithiobacillus caldus SM-1 strain=SM-1'
'Acidithiobacillus caldus ATCC 51756 strain=ATCC 51756'
'gamma proteobacterium HdN1'
'Gynuella sunshinyii YC6258 strain=YC6258'
'Spongiibacter sp. IMCC21906 strain=IMCC21906'
'Zhongshania aliphaticivorans strain=SM2'
'Cellvibrio japonicus Ueda107 strain=Ueda107'
'Teredinibacter turnerae T7901 strain=T7901'
'Saccharophagus degradans 2-40 strain=2-40'
'Marinobacter sp. BSs20148 strain=BSs20148'
'Marinobacter psychrophilus strain=20041'
'Marinobacter hydrocarbonoclasticus VT8 strain=VT8'
'Marinobacter hydrocarbonoclasticus ATCC 49840 strain=ATCC 49840'
'Marinobacter sp. LQ44 strain=LQ44'
'Marinobacter sp. CP1 strain=CP1'
'Marinobacter adhaerens HP15 strain=HP15'
'Oleispira antarctica RB-8'
'Thalassolituus oleivorans MIL-1 strain=MIL-1'
'Thalassolituus oleivorans R6-15 strain=R6-15'
'Oblitimonas alkaliphila strain=C6918'
'Oblitimonas alkaliphila strain=D3318'
'Oblitimonas alkaliphila strain=B4199'
'Oblitimonas alkaliphila strain=E1148'
'Oblitimonas alkaliphila strain=E1086'
'Pseudomonas cremoricolorata strain=ND07'
'Pseudomonas putida strain=PC2'
'Pseudomonas parafulva strain=CRS01-1'
'Pseudomonas putida strain=1A00316'
'Pseudomonas mosselii SJ10 strain=SJ10'
'Pseudomonas sp. CCOS 191 strain=CCOS 191'
'Pseudomonas sp. VLB120 strain=VLB120'
'Pseudomonas putida W619 strain=W619'
'Pseudomonas putida GB-1 strain=GB-1'
'Pseudomonas sp. URMO17WK12I11'
'Pseudomonas putida KT2440 strain=KT2440'
'Pseudomonas sp. JY-Q strain=JY-Q'
'Pseudomonas putida B6-2 strain=B6-2'
'Pseudomonas putida F1 strain=F1'
'Pseudomonas putida ND6 strain=ND6'
'Pseudomonas putida S12 strain=S12'
'Pseudomonas putida BIRD-1 strain=BIRD-1'
'Pseudomonas putida NBRC 14164 strain=NBRC 14164'
'Pseudomonas putida H8234 strain=H8234'
'Pseudomonas putida S13.1.2 strain=S13.1.2'
'Pseudomonas putida HB3267 strain=PC9'
'Pseudomonas sp. FGI182 strain=FGI182'
'Pseudomonas putida S16 strain=S16'
'Pseudomonas plecoglossicida strain=NyZ12'
'Pseudomonas monteilii SB3078 strain=SB3078'
'Pseudomonas putida strain=DLL-E4'
'Pseudomonas alkylphenolia strain=KL28'
'Pseudomonas monteilii strain=USDA-ARS-USMARC-56711'
'Pseudomonas sp. L10.10 strain=L10.10'
'Pseudomonas fragi strain=P121'
'Pseudomonas fluorescens strain=LBUM636'
'Pseudomonas fluorescens strain=UK4'
'Pseudomonas fluorescens strain=L321'
'Pseudomonas sp. TKP strain=TKP'
'Pseudomonas antarctica strain=PAMC 27494'
'Pseudomonas poae RE*1-1-14 strain=RE*1-1-14'
'Pseudomonas fluorescens strain=LBUM223'
'Pseudomonas fluorescens A506 strain=A506'
'Pseudomonas fluorescens strain=Pt14'
'Pseudomonas trivialis strain=IHBB745'
'Pseudomonas azotoformans strain=S4'
'Pseudomonas fluorescens strain=L228'
'Pseudomonas fluorescens SBW25 strain=SBW25'
'Pseudomonas fluorescens PICF7 strain=PICF7'
'Pseudomonas fluorescens strain=PCL1751'
'Pseudomonas fluorescens strain=FW300-N2E3'

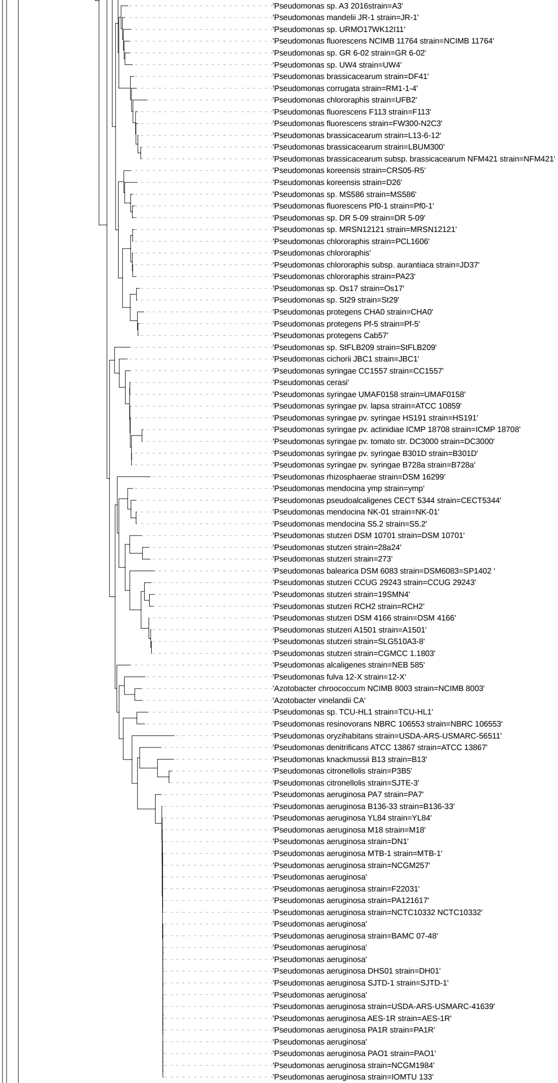
'Pseudomonas sp. A3 2016strain=A3'
'Pseudomonas mandelii JR-1 strain=JR-1'
'Pseudomonas sp. URMO17WK12I11'
'Pseudomonas fluorescens NCIMB 11764 strain=NCIMB 11764'
'Pseudomonas sp. GR 6-02 strain=GR 6-02'
'Pseudomonas sp. UW4 strain=UW4'
'Pseudomonas brassicacearum strain=DF41'
'Pseudomonas corrugata strain=RM1-1-4'
'Pseudomonas chlororaphis strain=UFB2'
'Pseudomonas fluorescens F113 strain=F113'
'Pseudomonas fluorescens strain=FW300-N2C3'
'Pseudomonas brassicacearum strain=L13-6-12'
'Pseudomonas brassicacearum strain=LBUM300'
'Pseudomonas brassicacearum subsp. brassicacearum NFM421 strain=NFM421'
'Pseudomonas koreensis strain=CRS05-R5'
'Pseudomonas koreensis strain=D26'
'Pseudomonas sp. MS586 strain=MS586'
'Pseudomonas fluorescens Pf0-1 strain=Pf0-1'
'Pseudomonas sp. DR 5-09 strain=DR 5-09'
'Pseudomonas sp. MRSN12121 strain=MRSN12121'
'Pseudomonas chlororaphis strain=PCL1606'
'Pseudomonas chlororaphis'
'Pseudomonas chlororaphis subsp. aurantiaca strain=JD37'
'Pseudomonas chlororaphis strain=PA23'
'Pseudomonas sp. Os17 strain=Os17'
'Pseudomonas sp. St29 strain=St29'
'Pseudomonas protegens CHA0 strain=CHA0'
'Pseudomonas protegens Pf-5 strain=Pf-5'
'Pseudomonas protegens Cab57'
'Pseudomonas sp. StFLB209 strain=StFLB209'
'Pseudomonas cichorii JBC1 strain=JBC1'
'Pseudomonas syringae CC1557 strain=CC1557'
'Pseudomonas cerasi'
'Pseudomonas syringae UMAF0158 strain=UMAF0158'
'Pseudomonas syringae pv. lapsa strain=ATCC 10859'
'Pseudomonas syringae pv. syringae HS191 strain=HS191'
'Pseudomonas syringae pv. actinidiae ICMP 18708 strain=ICMP 18708'
'Pseudomonas syringae pv. tomato str. DC3000 strain=DC3000'
'Pseudomonas syringae pv. syringae B301D strain=B301D'
'Pseudomonas syringae pv. syringae B728a strain=B728a'
'Pseudomonas rhizosphaerae strain=DSM 16299'
'Pseudomonas mendocina ymp strain=ymp'
'Pseudomonas pseudoalcaligenes CECT 5344 strain=CECT5344'
'Pseudomonas mendocina NK-01 strain=NK-01'
'Pseudomonas mendocina S5.2 strain=S5.2'
'Pseudomonas stutzeri DSM 10701 strain=DSM 10701'
'Pseudomonas stutzeri strain=28a24'
'Pseudomonas stutzeri strain=273'
'Pseudomonas balearica DSM 6083 strain=DSM6083=SP1402 '
'Pseudomonas stutzeri CCUG 29243 strain=CCUG 29243'
'Pseudomonas stutzeri strain=19SMN4'
'Pseudomonas stutzeri RCH2 strain=RCH2'
'Pseudomonas stutzeri DSM 4166 strain=DSM 4166'
'Pseudomonas stutzeri A1501 strain=A1501'
'Pseudomonas stutzeri strain=SLG510A3-8'
'Pseudomonas stutzeri strain=CGMCC 1.1803'
'Pseudomonas alcaligenes strain=NEB 585'
'Pseudomonas fulva 12-X strain=12-X'
'Azotobacter chroococcum NCIMB 8003 strain=NCIMB 8003'
'Azotobacter vinelandii CA'
'Pseudomonas sp. TCU-HL1 strain=TCU-HL1'
'Pseudomonas resinovorans NBRC 106553 strain=NBRC 106553'
'Pseudomonas oryzihabitans strain=USDA-ARS-USMARC-56511'
'Pseudomonas denitrificans ATCC 13867 strain=ATCC 13867'
'Pseudomonas knackmussii B13 strain=B13'
'Pseudomonas citronellolis strain=P3B5'
'Pseudomonas citronellolis strain=SJTE-3'
'Pseudomonas aeruginosa PA7 strain=PA7'
'Pseudomonas aeruginosa B136-33 strain=B136-33'
'Pseudomonas aeruginosa YL84 strain=YL84'
'Pseudomonas aeruginosa M18 strain=M18'
'Pseudomonas aeruginosa strain=DN1'
'Pseudomonas aeruginosa MTB-1 strain=MTB-1'
'Pseudomonas aeruginosa strain=NCGM257'
'Pseudomonas aeruginosa'
'Pseudomonas aeruginosa strain=F22031'
'Pseudomonas aeruginosa strain=PA121617'
'Pseudomonas aeruginosa strain=NCTC10332 NCTC10332'
'Pseudomonas aeruginosa'
'Pseudomonas aeruginosa strain=BAMC 07-48'
'Pseudomonas aeruginosa'
'Pseudomonas aeruginosa'
'Pseudomonas aeruginosa DHS01 strain=DH01'
'Pseudomonas aeruginosa SJTD-1 strain=SJTD-1'
'Pseudomonas aeruginosa'
'Pseudomonas aeruginosa strain=USDA-ARS-USMARC-41639'
'Pseudomonas aeruginosa AES-1R strain=AES-1R'
'Pseudomonas aeruginosa PA1R strain=PA1R'
'Pseudomonas aeruginosa'
'Pseudomonas aeruginosa PAO1 strain=PAO1'
'Pseudomonas aeruginosa strain=NCGM1984'
'Pseudomonas aeruginosa strain=IOMTU 133'

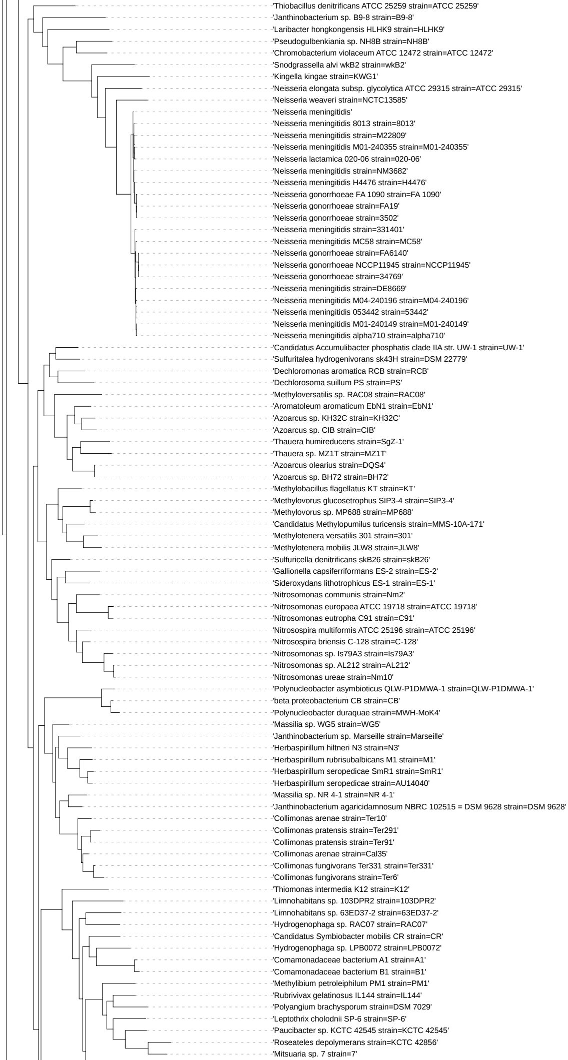

'Thiobacillus denitrificans ATCC 25259 strain=ATCC 25259'
'Janthinobacterium sp. B9-8 strain=B9-8'
'Laribacter hongkongensis HLHK9 strain=HLHK9'
'Pseudogulbenkiania sp. NH8B strain=NH8B'
'Chromobacterium violaceum ATCC 12472 strain=ATCC 12472'
'Snodgrassella alvi wkB2 strain=wkB2'
'Kingella kingae strain=KWG1'
'Neisseria elongata subsp. glycolytica ATCC 29315 strain=ATCC 29315'
'Neisseria weaveri strain=NCTC13585'
'Neisseria meningitidis'
'Neisseria meningitidis 8013 strain=8013'
'Neisseria meningitidis strain=M22809'
'Neisseria meningitidis M01-240355 strain=M01-240355'
'Neisseria lactamica 020-06 strain=020-06'
'Neisseria meningitidis strain=NM3682'
'Neisseria meningitidis H4476 strain=H4476'
'Neisseria gonorrhoeae FA 1090 strain=FA 1090'
'Neisseria gonorrhoeae strain=FA19'
'Neisseria gonorrhoeae strain=3502'
'Neisseria meningitidis strain=331401'
'Neisseria meningitidis MC58 strain=MC58'
'Neisseria gonorrhoeae strain=FA6140'
'Neisseria gonorrhoeae NCCP11945 strain=NCCP11945'
'Neisseria gonorrhoeae strain=34769'
'Neisseria meningitidis strain=DE8669'
'Neisseria meningitidis M04-240196 strain=M04-240196'
'Neisseria meningitidis 053442 strain=53442'
'Neisseria meningitidis M01-240149 strain=M01-240149'
'Neisseria meningitidis alpha710 strain=alpha710'
'Candidatus Accumulibacter phosphatis clade IIA str. UW-1 strain=UW-1'
'Sulfuritalea hydrogenivorans sk43H strain=DSM 22779'
'Dechloromonas aromatica RCB strain=RCB'
'Dechlorosoma suillum PS strain=PS'
'Methyloversatilis sp. RAC08 strain=RAC08'
'Aromatoleum aromaticum EbN1 strain=EbN1'
'Azoarcus sp. KH32C strain=KH32C'
'Azoarcus sp. CIB strain=CIB'
'Thauera humireducens strain=SgZ-1'
'Thauera sp. MZ1T strain=MZ1T'
'Azoarcus olearius strain=DQS4'
'Azoarcus sp. BH72 strain=BH72'
'Methylobacillus flagellatus KT strain=KT'
'Methylovorus glucosetrophus SIP3-4 strain=SIP3-4'
'Methylovorus sp. MP688 strain=MP688'
'Candidatus Methylopumilus turicensis strain=MMS-10A-171'
'Methylotenera versatilis 301 strain=301'
'Methylotenera mobilis JLW8 strain=JLW8'
'Sulfuricella denitrificans skB26 strain=skB26'
'Gallionella capsiferriformans ES-2 strain=ES-2'
'Sideroxydans lithotrophicus ES-1 strain=ES-1'
'Nitrosomonas communis strain=Nm2'
'Nitrosomonas europaea ATCC 19718 strain=ATCC 19718'
'Nitrosomonas eutropha C91 strain=C91'
'Nitrosospira multiformis ATCC 25196 strain=ATCC 25196'
'Nitrosospira briensis C-128 strain=C-128'
'Nitrosomonas sp. Is79A3 strain=Is79A3'
'Nitrosomonas sp. AL212 strain=AL212'
'Nitrosomonas ureae strain=Nm10'
'Polynucleobacter asymbioticus QLW-P1DMWA-1 strain=QLW-P1DMWA-1'
'beta proteobacterium CB strain=CB'
'Polynucleobacter duraquae strain=MWH-MoK4'
'Massilia sp. WG5 strain=WG5'
'Janthinobacterium sp. Marseille strain=Marseille'
'Herbaspirillum hiltneri N3 strain=N3'
'Herbaspirillum rubrisubalbicans M1 strain=M1'
'Herbaspirillum seropedicae SmR1 strain=SmR1'
'Herbaspirillum seropedicae strain=AU14040'
'Massilia sp. NR 4-1 strain=NR 4-1'
'Janthinobacterium agaricidamnosum NBRC 102515 = DSM 9628 strain=DSM 9628'
'Collimonas arenae strain=Ter10'
'Collimonas pratensis strain=Ter291'
'Collimonas pratensis strain=Ter91'
'Collimonas arenae strain=Cal35'
'Collimonas fungivorans Ter331 strain=Ter331'
'Collimonas fungivorans strain=Ter6'
'Thiomonas intermedia K12 strain=K12'
'Limnohabitans sp. 103DPR2 strain=103DPR2'
'Limnohabitans sp. 63ED37-2 strain=63ED37-2'
'Hydrogenophaga sp. RAC07 strain=RAC07'
'Candidatus Symbiobacter mobilis CR strain=CR'
'Hydrogenophaga sp. LPB0072 strain=LPB0072'
'Comamonadaceae bacterium A1 strain=A1'
'Comamonadaceae bacterium B1 strain=B1'
'Methylibium petroleiphilum PM1 strain=PM1'
'Rubrivivax gelatinosus IL144 strain=IL144'
'Polyangium brachysporum strain=DSM 7029'
'Leptothrix cholodnii SP-6 strain=SP-6'
'Paucibacter sp. KCTC 42545 strain=KCTC 42545'
'Roseateles depolymerans strain=KCTC 42856'
'Mitsuaria sp. 7 strain=7'

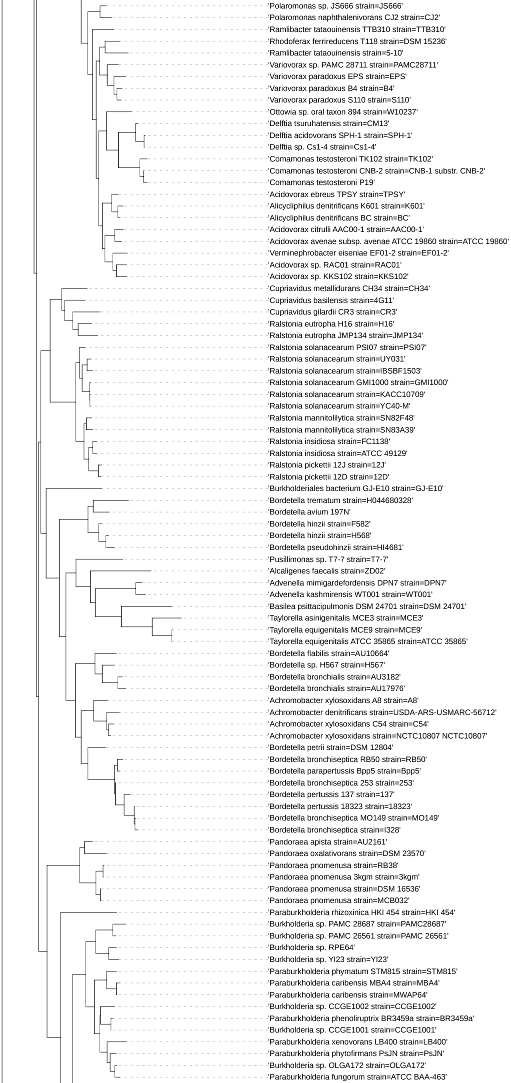
'Polaromonas sp. JS666 strain=JS666'
'Polaromonas naphthalenivorans CJ2 strain=CJ2'
'Ramlibacter tataouinensis TTB310 strain=TTB310'
'Rhodoferax ferrireducens T118 strain=DSM 15236'
'Ramlibacter tataouinensis strain=5-10'
'Variovorax sp. PAMC 28711 strain=PAMC28711'
'Variovorax paradoxus EPS strain=EPS'
'Variovorax paradoxus B4 strain=B4'
'Variovorax paradoxus S110 strain=S110'
'Ottowia sp. oral taxon 894 strain=W10237'
'Delftia tsuruhatensis strain=CM13'
'Delftia acidovorans SPH-1 strain=SPH-1'
'Delftia sp. Cs1-4 strain=Cs1-4'
'Comamonas testosteroni TK102 strain=TK102'
'Comamonas testosteroni CNB-2 strain=CNB-1 substr. CNB-2'
'Comamonas testosteroni P19'
'Acidovorax ebreus TPSY strain=TPSY'
'Alicycliphilus denitrificans K601 strain=K601'
'Alicycliphilus denitrificans BC strain=BC'
'Acidovorax citrulli AAC00-1 strain=AAC00-1'
'Acidovorax avenae subsp. avenae ATCC 19860 strain=ATCC 19860'
'Verminephrobacter eiseniae EF01-2 strain=EF01-2'
'Acidovorax sp. RAC01 strain=RAC01'
'Acidovorax sp. KKS102 strain=KKS102'
'Cupriavidus metallidurans CH34 strain=CH34'
'Cupriavidus basilensis strain=4G11'
'Cupriavidus gilardii CR3 strain=CR3'
'Ralstonia eutropha H16 strain=H16'
'Ralstonia eutropha JMP134 strain=JMP134'
'Ralstonia solanacearum PSI07 strain=PSI07'
'Ralstonia solanacearum strain=UY031'
'Ralstonia solanacearum strain=IBSBF1503'
'Ralstonia solanacearum GMI1000 strain=GMI1000'
'Ralstonia solanacearum strain=KACC10709'
'Ralstonia solanacearum strain=YC40-M'
'Ralstonia mannitolilytica strain=SN82F48'
'Ralstonia mannitolilytica strain=SN83A39'
'Ralstonia insidiosa strain=FC1138'
'Ralstonia insidiosa strain=ATCC 49129'
'Ralstonia pickettii 12J strain=12J'
'Ralstonia pickettii 12D strain=12D'
'Burkholderiales bacterium GJ-E10 strain=GJ-E10'
'Bordetella trematum strain=H044680328'
'Bordetella avium 197N'
'Bordetella hinzii strain=F582'
'Bordetella hinzii strain=H568'
'Bordetella pseudohinzii strain=HI4681'
'Pusillimonas sp. T7-7 strain=T7-7'
'Alcaligenes faecalis strain=ZD02'
'Advenella mimigardefordensis DPN7 strain=DPN7'
'Advenella kashmirensis WT001 strain=WT001'
'Basilea psittacipulmonis DSM 24701 strain=DSM 24701'
'Taylorella asinigenitalis MCE3 strain=MCE3'
'Taylorella equigenitalis MCE9 strain=MCE9'
'Taylorella equigenitalis ATCC 35865 strain=ATCC 35865'
'Bordetella flabilis strain=AU10664'
'Bordetella sp. H567 strain=H567'
'Bordetella bronchialis strain=AU3182'
'Bordetella bronchialis strain=AU17976'
'Achromobacter xylosoxidans A8 strain=A8'
'Achromobacter denitrificans strain=USDA-ARS-USMARC-56712'
'Achromobacter xylosoxidans C54 strain=C54'
'Achromobacter xylosoxidans strain=NCTC10807 NCTC10807'
'Bordetella petrii strain=DSM 12804'
'Bordetella bronchiseptica RB50 strain=RB50'
'Bordetella parapertussis Bpp5 strain=Bpp5'
'Bordetella bronchiseptica 253 strain=253'
'Bordetella pertussis 137 strain=137'
'Bordetella pertussis 18323 strain=18323'
'Bordetella bronchiseptica MO149 strain=MO149'
'Bordetella bronchiseptica strain=I328'
'Pandoraea apista strain=AU2161'
'Pandoraea oxalativorans strain=DSM 23570'
'Pandoraea pnomenusa strain=RB38'
'Pandoraea pnomenusa 3kgm strain=3kgm'
'Pandoraea pnomenusa strain=DSM 16536'
'Pandoraea pnomenusa strain=MCB032'
'Paraburkholderia rhizoxinica HKI 454 strain=HKI 454'
'Burkholderia sp. PAMC 28687 strain=PAMC28687'
'Burkholderia sp. PAMC 26561 strain=PAMC 26561'
'Burkholderia sp. RPE64'
'Burkholderia sp. YI23 strain=YI23'
'Paraburkholderia phymatum STM815 strain=STM815'
'Paraburkholderia caribensis MBA4 strain=MBA4'
'Paraburkholderia caribensis strain=MWAP64'
'Burkholderia sp. CCGE1002 strain=CCGE1002'
'Paraburkholderia phenoliruptrix BR3459a strain=BR3459a'
'Burkholderia sp. CCGE1001 strain=CCGE1001'
'Paraburkholderia xenovorans LB400 strain=LB400'
'Paraburkholderia phytofirmans PsJN strain=PsJN'
'Burkholderia sp. OLGA172 strain=OLGA172'
'Paraburkholderia fungorum strain=ATCC BAA-463'

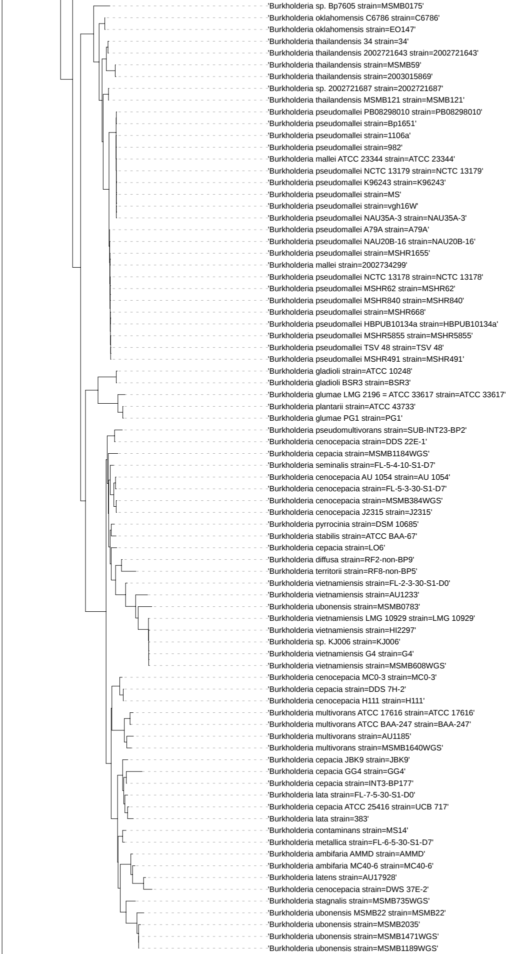
'Burkholderia sp. Bp7605 strain=MSMB0175'
'Burkholderia oklahomensis C6786 strain=C6786'
'Burkholderia oklahomensis strain=EO147'
'Burkholderia thailandensis 34 strain=34'
'Burkholderia thailandensis 2002721643 strain=2002721643'
'Burkholderia thailandensis strain=MSMB59'
'Burkholderia thailandensis strain=2003015869'
'Burkholderia sp. 2002721687 strain=2002721687'
'Burkholderia thailandensis MSMB121 strain=MSMB121'
'Burkholderia pseudomallei PB08298010 strain=PB08298010'
'Burkholderia pseudomallei strain=Bp1651'
'Burkholderia pseudomallei strain=1106a'
'Burkholderia pseudomallei strain=982'
'Burkholderia mallei ATCC 23344 strain=ATCC 23344'
'Burkholderia pseudomallei NCTC 13179 strain=NCTC 13179'
'Burkholderia pseudomallei K96243 strain=K96243'
'Burkholderia pseudomallei strain=MS'
'Burkholderia pseudomallei strain=vgh16W'
'Burkholderia pseudomallei NAU35A-3 strain=NAU35A-3'
'Burkholderia pseudomallei A79A strain=A79A'
'Burkholderia pseudomallei NAU20B-16 strain=NAU20B-16'
'Burkholderia pseudomallei strain=MSHR1655'
'Burkholderia mallei strain=2002734299'
'Burkholderia pseudomallei NCTC 13178 strain=NCTC 13178'
'Burkholderia pseudomallei MSHR62 strain=MSHR62'
'Burkholderia pseudomallei MSHR840 strain=MSHR840'
'Burkholderia pseudomallei strain=MSHR668'
'Burkholderia pseudomallei HBPUB10134a strain=HBPUB10134a'
'Burkholderia pseudomallei MSHR5855 strain=MSHR5855'
'Burkholderia pseudomallei TSV 48 strain=TSV 48'
'Burkholderia pseudomallei MSHR491 strain=MSHR491'
'Burkholderia gladioli strain=ATCC 10248'
'Burkholderia gladioli BSR3 strain=BSR3'
'Burkholderia glumae LMG 2196 = ATCC 33617 strain=ATCC 33617'
'Burkholderia plantarii strain=ATCC 43733'
'Burkholderia glumae PG1 strain=PG1'
'Burkholderia pseudomultivorans strain=SUB-INT23-BP2'
'Burkholderia cenocepacia strain=DDS 22E-1'
'Burkholderia cepacia strain=MSMB1184WGS'
'Burkholderia seminalis strain=FL-5-4-10-S1-D7'
'Burkholderia cenocepacia AU 1054 strain=AU 1054'
'Burkholderia cenocepacia strain=FL-5-3-30-S1-D7'
'Burkholderia cenocepacia strain=MSMB384WGS'
'Burkholderia cenocepacia J2315 strain=J2315'
'Burkholderia pyrrocinia strain=DSM 10685'
'Burkholderia stabilis strain=ATCC BAA-67'
'Burkholderia cepacia strain=LO6'
'Burkholderia diffusa strain=RF2-non-BP9'
'Burkholderia territorii strain=RF8-non-BP5'
'Burkholderia vietnamiensis strain=FL-2-3-30-S1-D0'
'Burkholderia vietnamiensis strain=AU1233'
'Burkholderia ubonensis strain=MSMB0783'
'Burkholderia vietnamiensis LMG 10929 strain=LMG 10929'
'Burkholderia vietnamiensis strain=HI2297'
'Burkholderia sp. KJ006 strain=KJ006'
'Burkholderia vietnamiensis G4 strain=G4'
'Burkholderia vietnamiensis strain=MSMB608WGS'
'Burkholderia cenocepacia MC0-3 strain=MC0-3'
'Burkholderia cepacia strain=DDS 7H-2'
'Burkholderia cenocepacia H111 strain=H111'
'Burkholderia multivorans ATCC 17616 strain=ATCC 17616'
'Burkholderia multivorans ATCC BAA-247 strain=BAA-247'
'Burkholderia multivorans strain=AU1185'
'Burkholderia multivorans strain=MSMB1640WGS'
'Burkholderia cepacia JBK9 strain=JBK9'
'Burkholderia cepacia GG4 strain=GG4'
'Burkholderia cepacia strain=INT3-BP177'
'Burkholderia lata strain=FL-7-5-30-S1-D0'
'Burkholderia cepacia ATCC 25416 strain=UCB 717'
'Burkholderia lata strain=383'
'Burkholderia contaminans strain=MS14'
'Burkholderia metallica strain=FL-6-5-30-S1-D7'
'Burkholderia ambifaria AMMD strain=AMMD'
'Burkholderia ambifaria MC40-6 strain=MC40-6'
'Burkholderia latens strain=AU17928'
'Burkholderia cenocepacia strain=DWS 37E-2'
'Burkholderia stagnalis strain=MSMB735WGS'
'Burkholderia ubonensis MSMB22 strain=MSMB22'
'Burkholderia ubonensis strain=MSMB2035'
'Burkholderia ubonensis strain=MSMB1471WGS'
'Burkholderia ubonensis strain=MSMB1189WGS'

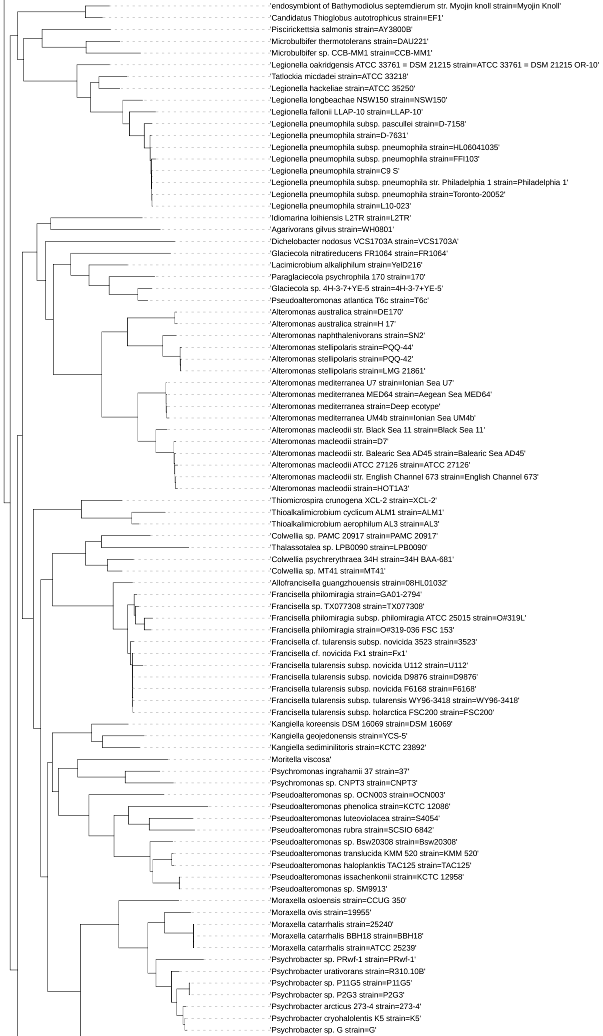
'endosymbiont of Bathymodiolus septemdierum str. Myojin knoll strain=Myojin Knoll'
'Candidatus Thioglobus autotrophicus strain=EF1'
'Piscirickettsia salmonis strain=AY3800B'
'Microbulbifer thermotolerans strain=DAU221'
'Microbulbifer sp. CCB-MM1 strain=CCB-MM1'
'Legionella oakridgensis ATCC 33761 = DSM 21215 strain=ATCC 33761 = DSM 21215 OR-10'
'Tatlockia micdadei strain=ATCC 33218'
'Legionella hackeliae strain=ATCC 35250'
'Legionella longbeachae NSW150 strain=NSW150'
'Legionella fallonii LLAP-10 strain=LLAP-10'
'Legionella pneumophila subsp. pascullei strain=D-7158'
'Legionella pneumophila strain=D-7631'
'Legionella pneumophila subsp. pneumophila strain=HL06041035'
'Legionella pneumophila subsp. pneumophila strain=FFI103'
'Legionella pneumophila strain=C9 S'
'Legionella pneumophila subsp. pneumophila str. Philadelphia 1 strain=Philadelphia 1'
'Legionella pneumophila subsp. pneumophila strain=Toronto-20052'
'Legionella pneumophila strain=L10-023'
'Idiomarina loihiensis L2TR strain=L2TR'
'Agarivorans gilvus strain=WH0801'
'Dichelobacter nodosus VCS1703A strain=VCS1703A'
'Glaciecola nitratireducens FR1064 strain=FR1064'
'Lacimicrobium alkaliphilum strain=YelD216'
'Paraglaciecola psychrophila 170 strain=170'
'Glaciecola sp. 4H-3-7+YE-5 strain=4H-3-7+YE-5'
'Pseudoalteromonas atlantica T6c strain=T6c'
'Alteromonas australica strain=DE170'
'Alteromonas australica strain=H 17'
'Alteromonas naphthalenivorans strain=SN2'
'Alteromonas stellipolaris strain=PQQ-44'
'Alteromonas stellipolaris strain=PQQ-42'
'Alteromonas stellipolaris strain=LMG 21861'
'Alteromonas mediterranea U7 strain=Ionian Sea U7'
'Alteromonas mediterranea MED64 strain=Aegean Sea MED64'
'Alteromonas mediterranea strain=Deep ecotype'
'Alteromonas mediterranea UM4b strain=Ionian Sea UM4b'
'Alteromonas macleodii str. Black Sea 11 strain=Black Sea 11'
'Alteromonas macleodii strain=D7'
'Alteromonas macleodii str. Balearic Sea AD45 strain=Balearic Sea AD45'
'Alteromonas macleodii ATCC 27126 strain=ATCC 27126'
'Alteromonas macleodii str. English Channel 673 strain=English Channel 673'
'Alteromonas macleodii strain=HOT1A3'
'Thiomicrospira crunogena XCL-2 strain=XCL-2'
'Thioalkalimicrobium cyclicum ALM1 strain=ALM1'
'Thioalkalimicrobium aerophilum AL3 strain=AL3'
'Colwellia sp. PAMC 20917 strain=PAMC 20917'
'Thalassotalea sp. LPB0090 strain=LPB0090'
'Colwellia psychrerythraea 34H strain=34H BAA-681'
'Colwellia sp. MT41 strain=MT41'
'Allofrancisella guangzhouensis strain=08HL01032'
'Francisella philomiragia strain=GA01-2794'
'Francisella sp. TX077308 strain=TX077308'
'Francisella philomiragia subsp. philomiragia ATCC 25015 strain=O#319L'
'Francisella philomiragia strain=O#319-036 FSC 153'
'Francisella cf. tularensis subsp. novicida 3523 strain=3523'
'Francisella cf. novicida Fx1 strain=Fx1'
'Francisella tularensis subsp. novicida U112 strain=U112'
'Francisella tularensis subsp. novicida D9876 strain=D9876'
'Francisella tularensis subsp. novicida F6168 strain=F6168'
'Francisella tularensis subsp. tularensis WY96-3418 strain=WY96-3418'
'Francisella tularensis subsp. holarctica FSC200 strain=FSC200'
'Kangiella koreensis DSM 16069 strain=DSM 16069'
'Kangiella geojedonensis strain=YCS-5'
'Kangiella sediminilitoris strain=KCTC 23892'
'Moritella viscosa'
'Psychromonas ingrahamii 37 strain=37'
'Psychromonas sp. CNPT3 strain=CNPT3'
'Pseudoalteromonas sp. OCN003 strain=OCN003'
'Pseudoalteromonas phenolica strain=KCTC 12086'
'Pseudoalteromonas luteoviolacea strain=S4054'
'Pseudoalteromonas rubra strain=SCSIO 6842'
'Pseudoalteromonas sp. Bsw20308 strain=Bsw20308'
'Pseudoalteromonas translucida KMM 520 strain=KMM 520'
'Pseudoalteromonas haloplanktis TAC125 strain=TAC125'
'Pseudoalteromonas issachenkonii strain=KCTC 12958'
'Pseudoalteromonas sp. SM9913'
'Moraxella osloensis strain=CCUG 350'
'Moraxella ovis strain=19955'
'Moraxella catarrhalis strain=25240'
'Moraxella catarrhalis BBH18 strain=BBH18'
'Moraxella catarrhalis strain=ATCC 25239'
'Psychrobacter sp. PRwf-1 strain=PRwf-1'
'Psychrobacter urativorans strain=R310.10B'
'Psychrobacter sp. P11G5 strain=P11G5'
'Psychrobacter sp. P2G3 strain=P2G3'
'Psychrobacter arcticus 273-4 strain=273-4'
'Psychrobacter cryohalolentis K5 strain=K5'
'Psychrobacter sp. G strain=G'

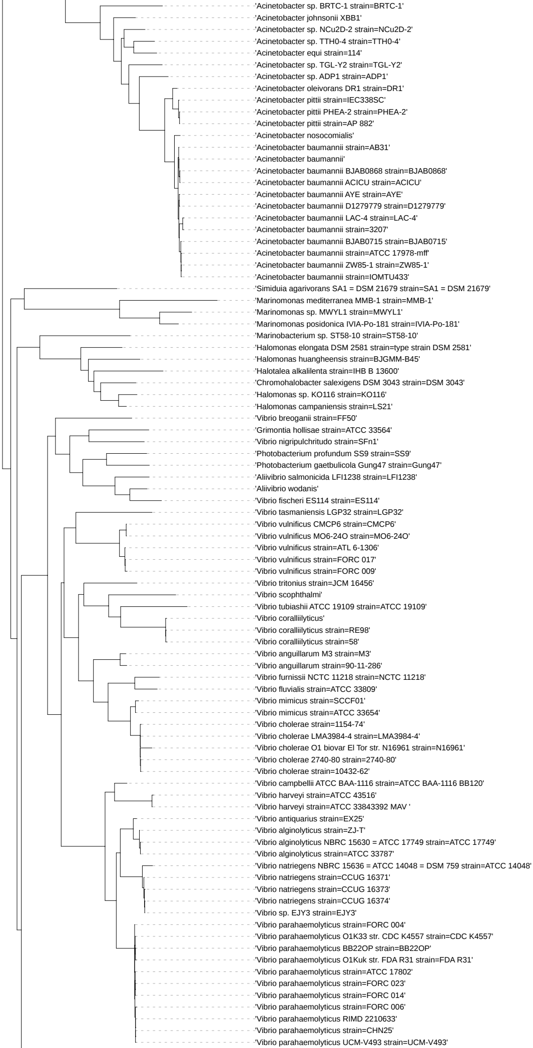
'Acinetobacter sp. BRTC-1 strain=BRTC-1'
'Acinetobacter johnsonii XBB1'
'Acinetobacter sp. NCu2D-2 strain=NCu2D-2'
'Acinetobacter sp. TTH0-4 strain=TTH0-4'
'Acinetobacter equi strain=114'
'Acinetobacter sp. TGL-Y2 strain=TGL-Y2'
'Acinetobacter sp. ADP1 strain=ADP1'
'Acinetobacter oleivorans DR1 strain=DR1'
'Acinetobacter pittii strain=IEC338SC'
'Acinetobacter pittii PHEA-2 strain=PHEA-2'
'Acinetobacter pittii strain=AP 882'
'Acinetobacter nosocomialis'
'Acinetobacter baumannii strain=AB31'
'Acinetobacter baumannii'
'Acinetobacter baumannii BJAB0868 strain=BJAB0868'
'Acinetobacter baumannii ACICU strain=ACICU'
'Acinetobacter baumannii AYE strain=AYE'
'Acinetobacter baumannii D1279779 strain=D1279779'
'Acinetobacter baumannii LAC-4 strain=LAC-4'
'Acinetobacter baumannii strain=3207'
'Acinetobacter baumannii BJAB0715 strain=BJAB0715'
'Acinetobacter baumannii strain=ATCC 17978-mff'
'Acinetobacter baumannii ZW85-1 strain=ZW85-1'
'Acinetobacter baumannii strain=IOMTU433'
'Simiduia agarivorans SA1 = DSM 21679 strain=SA1 = DSM 21679'
'Marinomonas mediterranea MMB-1 strain=MMB-1'
'Marinomonas sp. MWYL1 strain=MWYL1'
'Marinomonas posidonica IVIA-Po-181 strain=IVIA-Po-181'
'Marinobacterium sp. ST58-10 strain=ST58-10'
'Halomonas elongata DSM 2581 strain=type strain DSM 2581'
'Halomonas huangheensis strain=BJGMM-B45'
'Halotalea alkalilenta strain=IHB B 13600'
'Chromohalobacter salexigens DSM 3043 strain=DSM 3043'
'Halomonas sp. KO116 strain=KO116'
'Halomonas campaniensis strain=LS21'
'Vibrio breoganii strain=FF50'
'Grimontia hollisae strain=ATCC 33564'
'Vibrio nigripulchritudo strain=SFn1'
'Photobacterium profundum SS9 strain=SS9'
'Photobacterium gaetbulicola Gung47 strain=Gung47'
'Aliivibrio salmonicida LFI1238 strain=LFI1238'
'Aliivibrio wodanis'
'Vibrio fischeri ES114 strain=ES114'
'Vibrio tasmaniensis LGP32 strain=LGP32'
'Vibrio vulnificus CMCP6 strain=CMCP6'
'Vibrio vulnificus MO6-24O strain=MO6-24O'
'Vibrio vulnificus strain=ATL 6-1306'
'Vibrio vulnificus strain=FORC 017'
'Vibrio vulnificus strain=FORC 009'
'Vibrio tritonius strain=JCM 16456'
'Vibrio scophthalmi'
'Vibrio tubiashii ATCC 19109 strain=ATCC 19109'
'Vibrio coralliilyticus'
'Vibrio coralliilyticus strain=RE98'
'Vibrio coralliilyticus strain=58'
'Vibrio anguillarum M3 strain=M3'
'Vibrio anguillarum strain=90-11-286'
'Vibrio furnissii NCTC 11218 strain=NCTC 11218'
'Vibrio fluvialis strain=ATCC 33809'
'Vibrio mimicus strain=SCCF01'
'Vibrio mimicus strain=ATCC 33654'
'Vibrio cholerae strain=1154-74'
'Vibrio cholerae LMA3984-4 strain=LMA3984-4'
'Vibrio cholerae O1 biovar El Tor str. N16961 strain=N16961'
'Vibrio cholerae 2740-80 strain=2740-80'
'Vibrio cholerae strain=10432-62'
'Vibrio campbellii ATCC BAA-1116 strain=ATCC BAA-1116 BB120'
'Vibrio harveyi strain=ATCC 43516'
'Vibrio harveyi strain=ATCC 33843392 MAV '
'Vibrio antiquarius strain=EX25'
'Vibrio alginolyticus strain=ZJ-T'
'Vibrio alginolyticus NBRC 15630 = ATCC 17749 strain=ATCC 17749'
'Vibrio alginolyticus strain=ATCC 33787'
'Vibrio natriegens NBRC 15636 = ATCC 14048 = DSM 759 strain=ATCC 14048'
'Vibrio natriegens strain=CCUG 16371'
'Vibrio natriegens strain=CCUG 16373'
'Vibrio natriegens strain=CCUG 16374'
'Vibrio sp. EJY3 strain=EJY3'
'Vibrio parahaemolyticus strain=FORC 004'
'Vibrio parahaemolyticus O1K33 str. CDC K4557 strain=CDC K4557'
'Vibrio parahaemolyticus BB22OP strain=BB22OP'
'Vibrio parahaemolyticus O1Kuk str. FDA R31 strain=FDA R31'
'Vibrio parahaemolyticus strain=ATCC 17802'
'Vibrio parahaemolyticus strain=FORC 023'
'Vibrio parahaemolyticus strain=FORC 014'
'Vibrio parahaemolyticus strain=FORC 006'
'Vibrio parahaemolyticus RIMD 2210633'
'Vibrio parahaemolyticus strain=CHN25'
'Vibrio parahaemolyticus UCM-V493 strain=UCM-V493'

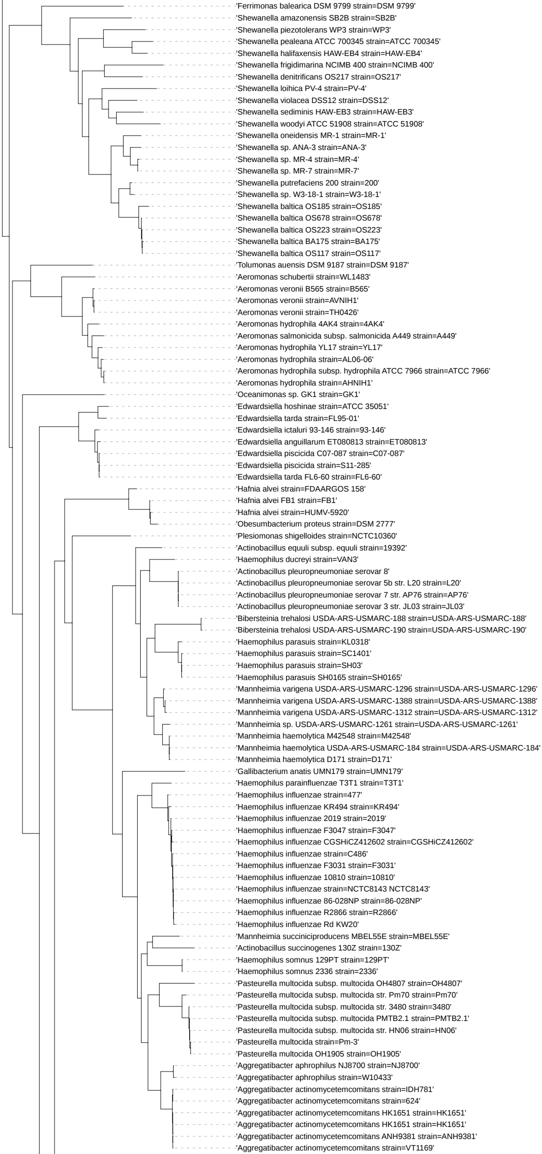
'Ferrimonas balearica DSM 9799 strain=DSM 9799'
'Shewanella amazonensis SB2B strain=SB2B'
'Shewanella piezotolerans WP3 strain=WP3'
'Shewanella pealeana ATCC 700345 strain=ATCC 700345'
'Shewanella halifaxensis HAW-EB4 strain=HAW-EB4'
'Shewanella frigidimarina NCIMB 400 strain=NCIMB 400'
'Shewanella denitrificans OS217 strain=OS217'
'Shewanella loihica PV-4 strain=PV-4'
'Shewanella violacea DSS12 strain=DSS12'
'Shewanella sediminis HAW-EB3 strain=HAW-EB3'
'Shewanella woodyi ATCC 51908 strain=ATCC 51908'
'Shewanella oneidensis MR-1 strain=MR-1'
'Shewanella sp. ANA-3 strain=ANA-3'
'Shewanella sp. MR-4 strain=MR-4'
'Shewanella sp. MR-7 strain=MR-7'
'Shewanella putrefaciens 200 strain=200'
'Shewanella sp. W3-18-1 strain=W3-18-1'
'Shewanella baltica OS185 strain=OS185'
'Shewanella baltica OS678 strain=OS678'
'Shewanella baltica OS223 strain=OS223'
'Shewanella baltica BA175 strain=BA175'
'Shewanella baltica OS117 strain=OS117'
'Tolumonas auensis DSM 9187 strain=DSM 9187'
'Aeromonas schubertii strain=WL1483'
'Aeromonas veronii B565 strain=B565'
'Aeromonas veronii strain=AVNIH1'
'Aeromonas veronii strain=TH0426'
'Aeromonas hydrophila 4AK4 strain=4AK4'
'Aeromonas salmonicida subsp. salmonicida A449 strain=A449'
'Aeromonas hydrophila YL17 strain=YL17'
'Aeromonas hydrophila strain=AL06-06'
'Aeromonas hydrophila subsp. hydrophila ATCC 7966 strain=ATCC 7966'
'Aeromonas hydrophila strain=AHNIH1'
'Oceanimonas sp. GK1 strain=GK1'
'Edwardsiella hoshinae strain=ATCC 35051'
'Edwardsiella tarda strain=FL95-01'
'Edwardsiella ictaluri 93-146 strain=93-146'
'Edwardsiella anguillarum ET080813 strain=ET080813'
'Edwardsiella piscicida C07-087 strain=C07-087'
'Edwardsiella piscicida strain=S11-285'
'Edwardsiella tarda FL6-60 strain=FL6-60'
'Hafnia alvei strain=FDAARGOS 158'
'Hafnia alvei FB1 strain=FB1'
'Hafnia alvei strain=HUMV-5920'
'Obesumbacterium proteus strain=DSM 2777'
'Plesiomonas shigelloides strain=NCTC10360'
'Actinobacillus equuli subsp. equuli strain=19392'
'Haemophilus ducreyi strain=VAN3'
'Actinobacillus pleuropneumoniae serovar 8'
'Actinobacillus pleuropneumoniae serovar 5b str. L20 strain=L20'
'Actinobacillus pleuropneumoniae serovar 7 str. AP76 strain=AP76'
'Actinobacillus pleuropneumoniae serovar 3 str. JL03 strain=JL03'
'Bibersteinia trehalosi USDA-ARS-USMARC-188 strain=USDA-ARS-USMARC-188'
'Bibersteinia trehalosi USDA-ARS-USMARC-190 strain=USDA-ARS-USMARC-190'
'Haemophilus parasuis strain=KL0318'
'Haemophilus parasuis strain=SC1401'
'Haemophilus parasuis strain=SH03'
'Haemophilus parasuis SH0165 strain=SH0165'
'Mannheimia varigena USDA-ARS-USMARC-1296 strain=USDA-ARS-USMARC-1296'
'Mannheimia varigena USDA-ARS-USMARC-1388 strain=USDA-ARS-USMARC-1388'
'Mannheimia varigena USDA-ARS-USMARC-1312 strain=USDA-ARS-USMARC-1312'
'Mannheimia sp. USDA-ARS-USMARC-1261 strain=USDA-ARS-USMARC-1261'
'Mannheimia haemolytica M42548 strain=M42548'
'Mannheimia haemolytica USDA-ARS-USMARC-184 strain=USDA-ARS-USMARC-184'
'Mannheimia haemolytica D171 strain=D171'
'Gallibacterium anatis UMN179 strain=UMN179'
'Haemophilus parainfluenzae T3T1 strain=T3T1'
'Haemophilus influenzae strain=477'
'Haemophilus influenzae KR494 strain=KR494'
'Haemophilus influenzae 2019 strain=2019'
'Haemophilus influenzae F3047 strain=F3047'
'Haemophilus influenzae CGSHiCZ412602 strain=CGSHiCZ412602'
'Haemophilus influenzae strain=C486'
'Haemophilus influenzae F3031 strain=F3031'
'Haemophilus influenzae 10810 strain=10810'
'Haemophilus influenzae strain=NCTC8143 NCTC8143'
'Haemophilus influenzae 86-028NP strain=86-028NP'
'Haemophilus influenzae R2866 strain=R2866'
'Haemophilus influenzae Rd KW20'
'Mannheimia succiniciproducens MBEL55E strain=MBEL55E'
'Actinobacillus succinogenes 130Z strain=130Z'
'Haemophilus somnus 129PT strain=129PT'
'Haemophilus somnus 2336 strain=2336'
'Pasteurella multocida subsp. multocida OH4807 strain=OH4807'
'Pasteurella multocida subsp. multocida str. Pm70 strain=Pm70'
'Pasteurella multocida subsp. multocida str. 3480 strain=3480'
'Pasteurella multocida subsp. multocida PMTB2.1 strain=PMTB2.1'
'Pasteurella multocida subsp. multocida str. HN06 strain=HN06'
'Pasteurella multocida strain=Pm-3'
'Pasteurella multocida OH1905 strain=OH1905'
'Aggregatibacter aphrophilus NJ8700 strain=NJ8700'
'Aggregatibacter aphrophilus strain=W10433'
'Aggregatibacter actinomycetemcomitans strain=IDH781'
'Aggregatibacter actinomycetemcomitans strain=624'
'Aggregatibacter actinomycetemcomitans HK1651 strain=HK1651'
'Aggregatibacter actinomycetemcomitans HK1651 strain=HK1651'
'Aggregatibacter actinomycetemcomitans ANH9381 strain=ANH9381'
'Aggregatibacter actinomycetemcomitans strain=VT1169'

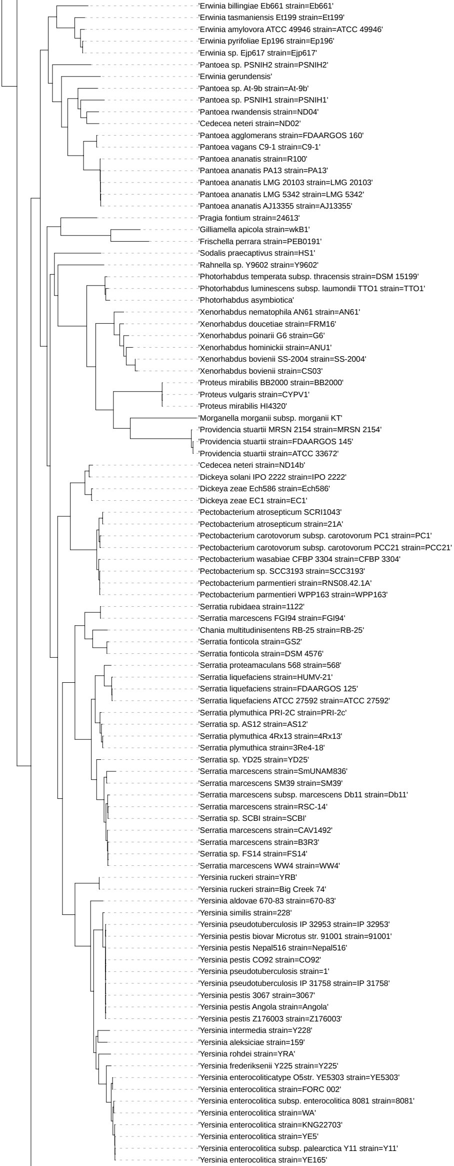
'Erwinia billingiae Eb661 strain=Eb661'
'Erwinia tasmaniensis Et199 strain=Et199'
'Erwinia amylovora ATCC 49946 strain=ATCC 49946'
'Erwinia pyrifoliae Ep196 strain=Ep196'
'Erwinia sp. Ejp617 strain=Ejp617'
'Pantoea sp. PSNIH2 strain=PSNIH2'
'Erwinia gerundensis'
'Pantoea sp. At-9b strain=At-9b'
'Pantoea sp. PSNIH1 strain=PSNIH1'
'Pantoea rwandensis strain=ND04'
'Cedecea neteri strain=ND02'
'Pantoea agglomerans strain=FDAARGOS 160'
'Pantoea vagans C9-1 strain=C9-1'
'Pantoea ananatis strain=R100'
'Pantoea ananatis PA13 strain=PA13'
'Pantoea ananatis LMG 20103 strain=LMG 20103'
'Pantoea ananatis LMG 5342 strain=LMG 5342'
'Pantoea ananatis AJ13355 strain=AJ13355'
'Pragia fontium strain=24613'
'Gilliamella apicola strain=wkB1'
'Frischella perrara strain=PEB0191'
'Sodalis praecaptivus strain=HS1'
'Rahnella sp. Y9602 strain=Y9602'
'Photorhabdus temperata subsp. thracensis strain=DSM 15199'
'Photorhabdus luminescens subsp. laumondii TTO1 strain=TTO1'
'Photorhabdus asymbiotica'
'Xenorhabdus nematophila AN61 strain=AN61'
'Xenorhabdus doucetiae strain=FRM16'
'Xenorhabdus poinarii G6 strain=G6'
'Xenorhabdus hominickii strain=ANU1'
'Xenorhabdus bovienii SS-2004 strain=SS-2004'
'Xenorhabdus bovienii strain=CS03'
'Proteus mirabilis BB2000 strain=BB2000'
'Proteus vulgaris strain=CYPV1'
'Proteus mirabilis HI4320'
'Morganella morganii subsp. morganii KT'
'Providencia stuartii MRSN 2154 strain=MRSN 2154'
'Providencia stuartii strain=FDAARGOS 145'
'Providencia stuartii strain=ATCC 33672'
'Cedecea neteri strain=ND14b'
'Dickeya solani IPO 2222 strain=IPO 2222'
'Dickeya zeae Ech586 strain=Ech586'
'Dickeya zeae EC1 strain=EC1'
'Pectobacterium atrosepticum SCRI1043'
'Pectobacterium atrosepticum strain=21A'
'Pectobacterium carotovorum subsp. carotovorum PC1 strain=PC1'
'Pectobacterium carotovorum subsp. carotovorum PCC21 strain=PCC21'
'Pectobacterium wasabiae CFBP 3304 strain=CFBP 3304'
'Pectobacterium sp. SCC3193 strain=SCC3193'
'Pectobacterium parmentieri strain=RNS08.42.1A'
'Pectobacterium parmentieri WPP163 strain=WPP163'
'Serratia rubidaea strain=1122'
'Serratia marcescens FGI94 strain=FGI94'
'Chania multitudinisentens RB-25 strain=RB-25'
'Serratia fonticola strain=GS2'
'Serratia fonticola strain=DSM 4576'
'Serratia proteamaculans 568 strain=568'
'Serratia liquefaciens strain=HUMV-21'
'Serratia liquefaciens strain=FDAARGOS 125'
'Serratia liquefaciens ATCC 27592 strain=ATCC 27592'
'Serratia plymuthica PRI-2C strain=PRI-2c'
'Serratia sp. AS12 strain=AS12'
'Serratia plymuthica 4Rx13 strain=4Rx13'
'Serratia plymuthica strain=3Re4-18'
'Serratia sp. YD25 strain=YD25'
'Serratia marcescens strain=SmUNAM836'
'Serratia marcescens SM39 strain=SM39'
'Serratia marcescens subsp. marcescens Db11 strain=Db11'
'Serratia marcescens strain=RSC-14'
'Serratia sp. SCBI strain=SCBI'
'Serratia marcescens strain=CAV1492'
'Serratia marcescens strain=B3R3'
'Serratia sp. FS14 strain=FS14'
'Serratia marcescens WW4 strain=WW4'
'Yersinia ruckeri strain=YRB'
'Yersinia ruckeri strain=Big Creek 74'
'Yersinia aldovae 670-83 strain=670-83'
'Yersinia similis strain=228'
'Yersinia pseudotuberculosis IP 32953 strain=IP 32953'
'Yersinia pestis biovar Microtus str. 91001 strain=91001'
'Yersinia pestis Nepal516 strain=Nepal516'
'Yersinia pestis CO92 strain=CO92'
'Yersinia pseudotuberculosis strain=1'
'Yersinia pseudotuberculosis IP 31758 strain=IP 31758'
'Yersinia pestis 3067 strain=3067'
'Yersinia pestis Angola strain=Angola'
'Yersinia pestis Z176003 strain=Z176003'
'Yersinia intermedia strain=Y228'
'Yersinia aleksiciae strain=159'
'Yersinia rohdei strain=YRA'
'Yersinia frederiksenii Y225 strain=Y225'
'Yersinia enterocoliticatype O5str. YE5303 strain=YE5303'
'Yersinia enterocolitica strain=FORC 002'
'Yersinia enterocolitica subsp. enterocolitica 8081 strain=8081'
'Yersinia enterocolitica strain=WA'
'Yersinia enterocolitica strain=KNG22703'
'Yersinia enterocolitica strain=YE5'
'Yersinia enterocolitica subsp. palearctica Y11 strain=Y11'
'Yersinia enterocolitica strain=YE165'

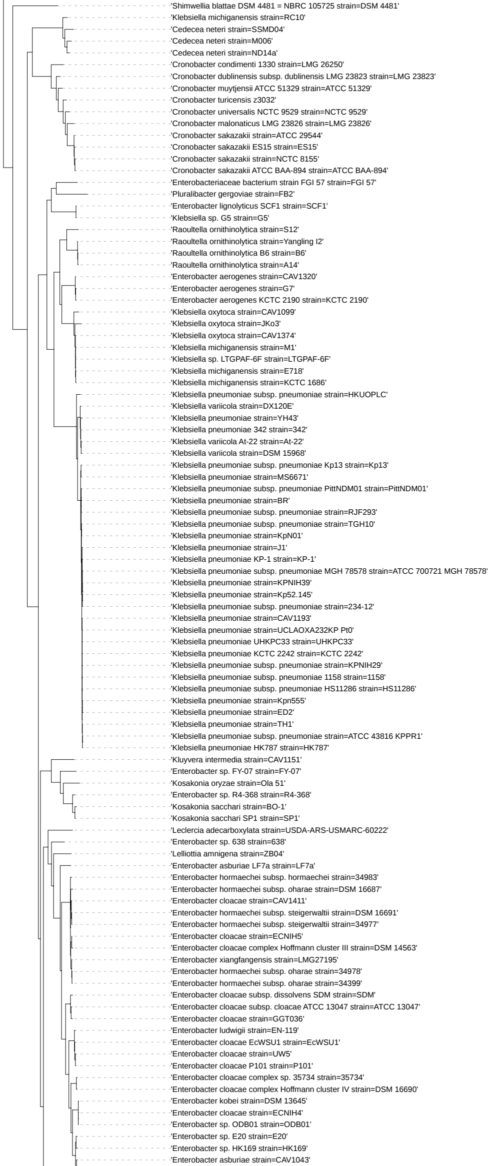
'Shimwellia blattae DSM 4481 = NBRC 105725 strain=DSM 4481'
'Klebsiella michiganensis strain=RC10'
'Cedecea neteri strain=SSMD04'
'Cedecea neteri strain=M006'
'Cedecea neteri strain=ND14a'
'Cronobacter condimenti 1330 strain=LMG 26250'
'Cronobacter dublinensis subsp. dublinensis LMG 23823 strain=LMG 23823'
'Cronobacter muytjensii ATCC 51329 strain=ATCC 51329'
'Cronobacter turicensis z3032'
'Cronobacter universalis NCTC 9529 strain=NCTC 9529'
'Cronobacter malonaticus LMG 23826 strain=LMG 23826'
'Cronobacter sakazakii strain=ATCC 29544'
'Cronobacter sakazakii ES15 strain=ES15'
'Cronobacter sakazakii strain=NCTC 8155'
'Cronobacter sakazakii ATCC BAA-894 strain=ATCC BAA-894'
'Enterobacteriaceae bacterium strain FGI 57 strain=FGI 57'
'Pluralibacter gergoviae strain=FB2'
'Enterobacter lignolyticus SCF1 strain=SCF1'
'Klebsiella sp. G5 strain=G5'
'Raoultella ornithinolytica strain=S12'
'Raoultella ornithinolytica strain=Yangling I2'
'Raoultella ornithinolytica B6 strain=B6'
'Raoultella ornithinolytica strain=A14'
'Enterobacter aerogenes strain=CAV1320'
'Enterobacter aerogenes strain=G7'
'Enterobacter aerogenes KCTC 2190 strain=KCTC 2190'
'Klebsiella oxytoca strain=CAV1099'
'Klebsiella oxytoca strain=JKo3'
'Klebsiella oxytoca strain=CAV1374'
'Klebsiella michiganensis strain=M1'
'Klebsiella sp. LTGPAF-6F strain=LTGPAF-6F'
'Klebsiella michiganensis strain=E718'
'Klebsiella michiganensis strain=KCTC 1686'
'Klebsiella pneumoniae subsp. pneumoniae strain=HKUOPLC'
'Klebsiella variicola strain=DX120E'
'Klebsiella pneumoniae strain=YH43'
'Klebsiella pneumoniae 342 strain=342'
'Klebsiella variicola At-22 strain=At-22'
'Klebsiella variicola strain=DSM 15968'
'Klebsiella pneumoniae subsp. pneumoniae Kp13 strain=Kp13'
'Klebsiella pneumoniae strain=MS6671'
'Klebsiella pneumoniae subsp. pneumoniae PittNDM01 strain=PittNDM01'
'Klebsiella pneumoniae strain=BR'
'Klebsiella pneumoniae subsp. pneumoniae strain=RJF293'
'Klebsiella pneumoniae subsp. pneumoniae strain=TGH10'
'Klebsiella pneumoniae strain=KpN01'
'Klebsiella pneumoniae strain=J1'
'Klebsiella pneumoniae KP-1 strain=KP-1'
'Klebsiella pneumoniae subsp. pneumoniae MGH 78578 strain=ATCC 700721 MGH 78578'
'Klebsiella pneumoniae strain=KPNIH39'
'Klebsiella pneumoniae strain=Kp52.145'
'Klebsiella pneumoniae subsp. pneumoniae strain=234-12'
'Klebsiella pneumoniae strain=CAV1193'
'Klebsiella pneumoniae strain=UCLAOXA232KP Pt0'
'Klebsiella pneumoniae UHKPC33 strain=UHKPC33'
'Klebsiella pneumoniae KCTC 2242 strain=KCTC 2242'
'Klebsiella pneumoniae subsp. pneumoniae strain=KPNIH29'
'Klebsiella pneumoniae subsp. pneumoniae 1158 strain=1158'
'Klebsiella pneumoniae subsp. pneumoniae HS11286 strain=HS11286'
'Klebsiella pneumoniae strain=Kpn555'
'Klebsiella pneumoniae strain=ED2'
'Klebsiella pneumoniae strain=TH1'
'Klebsiella pneumoniae subsp. pneumoniae strain=ATCC 43816 KPPR1'
'Klebsiella pneumoniae HK787 strain=HK787'
'Kluyvera intermedia strain=CAV1151'
'Enterobacter sp. FY-07 strain=FY-07'
'Kosakonia oryzae strain=Ola 51'
'Enterobacter sp. R4-368 strain=R4-368'
'Kosakonia sacchari strain=BO-1'
'Kosakonia sacchari SP1 strain=SP1'
'Leclercia adecarboxylata strain=USDA-ARS-USMARC-60222'
'Enterobacter sp. 638 strain=638'
'Lelliottia amnigena strain=ZB04'
'Enterobacter asburiae LF7a strain=LF7a'
'Enterobacter hormaechei subsp. hormaechei strain=34983'
'Enterobacter hormaechei subsp. oharae strain=DSM 16687'
'Enterobacter cloacae strain=CAV1411'
'Enterobacter hormaechei subsp. steigerwaltii strain=DSM 16691'
'Enterobacter hormaechei subsp. steigerwaltii strain=34977'
'Enterobacter cloacae strain=ECNIH5'
'Enterobacter cloacae complex Hoffmann cluster III strain=DSM 14563'
'Enterobacter xiangfangensis strain=LMG27195'
'Enterobacter hormaechei subsp. oharae strain=34978'
'Enterobacter hormaechei subsp. oharae strain=34399'
'Enterobacter cloacae subsp. dissolvens SDM strain=SDM'
'Enterobacter cloacae subsp. cloacae ATCC 13047 strain=ATCC 13047'
'Enterobacter cloacae strain=GGT036'
'Enterobacter ludwigii strain=EN-119'
'Enterobacter cloacae EcWSU1 strain=EcWSU1'
'Enterobacter cloacae strain=UW5'
'Enterobacter cloacae P101 strain=P101'
'Enterobacter cloacae complex sp. 35734 strain=35734'
'Enterobacter cloacae complex Hoffmann cluster IV strain=DSM 16690'
'Enterobacter kobei strain=DSM 13645'
'Enterobacter cloacae strain=ECNIH4'
'Enterobacter sp. ODB01 strain=ODB01'
'Enterobacter sp. E20 strain=E20'
'Enterobacter sp. HK169 strain=HK169'
'Enterobacter asburiae strain=CAV1043'

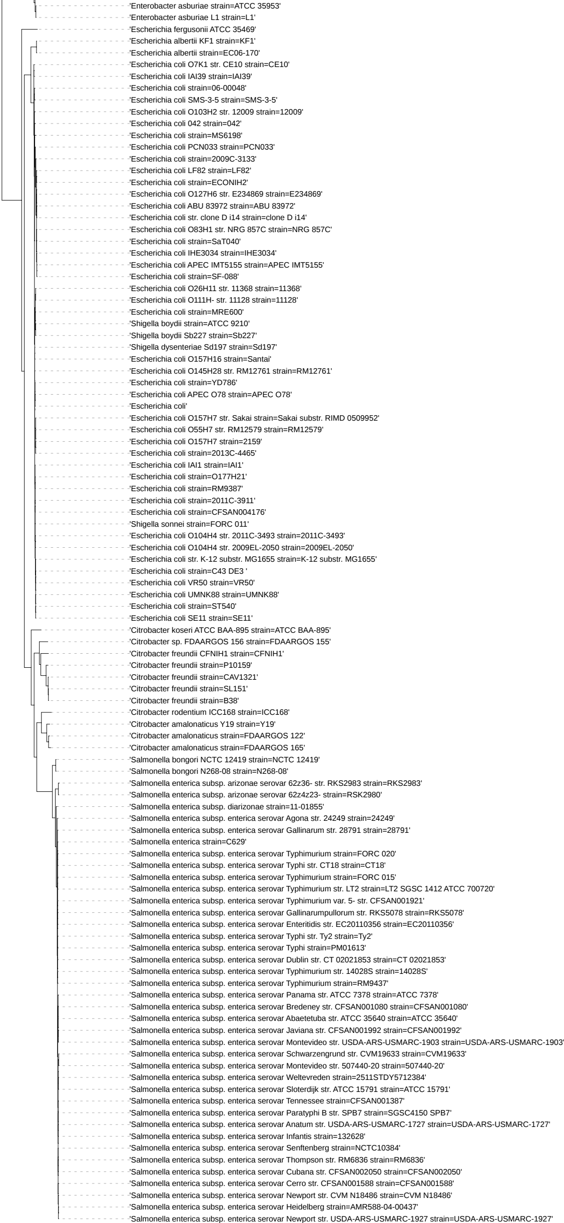
'Enterobacter asburiae strain=ATCC 35953'
'Enterobacter asburiae L1 strain=L1'
'Escherichia fergusonii ATCC 35469'
'Escherichia albertii KF1 strain=KF1'
'Escherichia albertii strain=EC06-170'
'Escherichia coli O7K1 str. CE10 strain=CE10'
'Escherichia coli IAI39 strain=IAI39'
'Escherichia coli strain=06-00048'
'Escherichia coli SMS-3-5 strain=SMS-3-5'
'Escherichia coli O103H2 str. 12009 strain=12009'
'Escherichia coli 042 strain=042'
'Escherichia coli strain=MS6198'
'Escherichia coli PCN033 strain=PCN033'
'Escherichia coli strain=2009C-3133'
'Escherichia coli LF82 strain=LF82'
'Escherichia coli strain=ECONIH2'
'Escherichia coli O127H6 str. E234869 strain=E234869'
'Escherichia coli ABU 83972 strain=ABU 83972'
'Escherichia coli str. clone D i14 strain=clone D i14'
'Escherichia coli O83H1 str. NRG 857C strain=NRG 857C'
'Escherichia coli strain=SaT040'
'Escherichia coli IHE3034 strain=IHE3034'
'Escherichia coli APEC IMT5155 strain=APEC IMT5155'
'Escherichia coli strain=SF-088'
'Escherichia coli O26H11 str. 11368 strain=11368'
'Escherichia coli O111H- str. 11128 strain=11128'
'Escherichia coli strain=MRE600'
'Shigella boydii strain=ATCC 9210'
'Shigella boydii Sb227 strain=Sb227'
'Shigella dysenteriae Sd197 strain=Sd197'
'Escherichia coli O157H16 strain=Santai'
'Escherichia coli O145H28 str. RM12761 strain=RM12761'
'Escherichia coli strain=YD786'
'Escherichia coli APEC O78 strain=APEC O78'
'Escherichia coli'
'Escherichia coli O157H7 str. Sakai strain=Sakai substr. RIMD 0509952'
'Escherichia coli O55H7 str. RM12579 strain=RM12579'
'Escherichia coli O157H7 strain=2159'
'Escherichia coli strain=2013C-4465'
'Escherichia coli IAI1 strain=IAI1'
'Escherichia coli strain=O177H21'
'Escherichia coli strain=RM9387'
'Escherichia coli strain=2011C-3911'
'Escherichia coli strain=CFSAN004176'
'Shigella sonnei strain=FORC 011'
'Escherichia coli O104H4 str. 2011C-3493 strain=2011C-3493'
'Escherichia coli O104H4 str. 2009EL-2050 strain=2009EL-2050'
'Escherichia coli str. K-12 substr. MG1655 strain=K-12 substr. MG1655'
'Escherichia coli strain=C43 DE3 '
'Escherichia coli VR50 strain=VR50'
'Escherichia coli UMNK88 strain=UMNK88'
'Escherichia coli strain=ST540'
'Escherichia coli SE11 strain=SE11'
'Citrobacter koseri ATCC BAA-895 strain=ATCC BAA-895'
'Citrobacter sp. FDAARGOS 156 strain=FDAARGOS 155'
'Citrobacter freundii CFNIH1 strain=CFNIH1'
'Citrobacter freundii strain=P10159'
'Citrobacter freundii strain=CAV1321'
'Citrobacter freundii strain=SL151'
'Citrobacter freundii strain=B38'
'Citrobacter rodentium ICC168 strain=ICC168'
'Citrobacter amalonaticus Y19 strain=Y19'
'Citrobacter amalonaticus strain=FDAARGOS 122'
'Citrobacter amalonaticus strain=FDAARGOS 165'
'Salmonella bongori NCTC 12419 strain=NCTC 12419'
'Salmonella bongori N268-08 strain=N268-08'
'Salmonella enterica subsp. arizonae serovar 62z36- str. RKS2983 strain=RKS2983'
'Salmonella enterica subsp. arizonae serovar 62z4z23- strain=RSK2980'
'Salmonella enterica subsp. diarizonae strain=11-01855'
'Salmonella enterica subsp. enterica serovar Agona str. 24249 strain=24249'
'Salmonella enterica subsp. enterica serovar Gallinarum str. 28791 strain=28791'
'Salmonella enterica strain=C629'
'Salmonella enterica subsp. enterica serovar Typhimurium strain=FORC 020'
'Salmonella enterica subsp. enterica serovar Typhi str. CT18 strain=CT18'
'Salmonella enterica subsp. enterica serovar Typhimurium strain=FORC 015'
'Salmonella enterica subsp. enterica serovar Typhimurium str. LT2 strain=LT2 SGSC 1412 ATCC 700720'
'Salmonella enterica subsp. enterica serovar Typhimurium var. 5- str. CFSAN001921'
'Salmonella enterica subsp. enterica serovar Gallinarumpullorum str. RKS5078 strain=RKS5078'
'Salmonella enterica subsp. enterica serovar Enteritidis str. EC20110356 strain=EC20110356'
'Salmonella enterica subsp. enterica serovar Typhi str. Ty2 strain=Ty2'
'Salmonella enterica subsp. enterica serovar Typhi strain=PM01613'
'Salmonella enterica subsp. enterica serovar Dublin str. CT 02021853 strain=CT 02021853'
'Salmonella enterica subsp. enterica serovar Typhimurium str. 14028S strain=14028S'
'Salmonella enterica subsp. enterica serovar Typhimurium strain=RM9437'
'Salmonella enterica subsp. enterica serovar Panama str. ATCC 7378 strain=ATCC 7378'
'Salmonella enterica subsp. enterica serovar Bredeney str. CFSAN001080 strain=CFSAN001080'
'Salmonella enterica subsp. enterica serovar Abaetetuba str. ATCC 35640 strain=ATCC 35640'
'Salmonella enterica subsp. enterica serovar Javiana str. CFSAN001992 strain=CFSAN001992'
'Salmonella enterica subsp. enterica serovar Montevideo str. USDA-ARS-USMARC-1903 strain=USDA-ARS-USMARC-1903
'Salmonella enterica subsp. enterica serovar Schwarzengrund str. CVM19633 strain=CVM19633'
'Salmonella enterica subsp. enterica serovar Montevideo str. 507440-20 strain=507440-20'
'Salmonella enterica subsp. enterica serovar Weltevreden strain=2511STDY5712384'
'Salmonella enterica subsp. enterica serovar Sloterdijk str. ATCC 15791 strain=ATCC 15791'
'Salmonella enterica subsp. enterica serovar Tennessee strain=CFSAN001387'
'Salmonella enterica subsp. enterica serovar Paratyphi B str. SPB7 strain=SGSC4150 SPB7'
'Salmonella enterica subsp. enterica serovar Anatum str. USDA-ARS-USMARC-1727 strain=USDA-ARS-USMARC-1727'
'Salmonella enterica subsp. enterica serovar Infantis strain=132628'
'Salmonella enterica subsp. enterica serovar Senftenberg strain=NCTC10384'
'Salmonella enterica subsp. enterica serovar Thompson str. RM6836 strain=RM6836'
'Salmonella enterica subsp. enterica serovar Cubana str. CFSAN002050 strain=CFSAN002050'
'Salmonella enterica subsp. enterica serovar Cerro str. CFSAN001588 strain=CFSAN001588'
'Salmonella enterica subsp. enterica serovar Newport str. CVM N18486 strain=CVM N18486'
'Salmonella enterica subsp. enterica serovar Heidelberg strain=AMR588-04-00437'
'Salmonella enterica subsp. enterica serovar Newport str. USDA-ARS-USMARC-1927 strain=USDA-ARS-USMARC-1927'
