# Supplementary material for: Evolution of the Natural Transformation Protein, ComEC, in Bacteria
Source: Front Microbiol. 2018 Dec 12;9:2980. doi: 10.3389/fmicb.2018.02980 (PMC6299819; doi:10.3389/fmicb.2018.02980)
Supplement: DATA SHEET S1 — Manual operations for the visualization of the species phylogeny. [file Data_Sheet_1.pdf]

Supplementary Text:

Evolution of the natural transformation protein, ComEC, in bacteria

Zachary Pimentel[1] and Ying Zhang[1,*]

[1] Department of Cell and Molecular Biology, University of Rhode Island, Kingston, RI, 02881, USA

* To whom correspondence should be addressed. Tel: +1 401 874 4915; Email: yingzhang@uri.edu

Manual operations for the visualization of the species phylogeny: In the visualization of bacterial phylogeny (Figure 2), taxa that contained a single representative species were eliminated from the phylogeny to enhance the visualization of major branches. The eliminated single lineages included *Caldisericum exile* AZM16cD1 of the Caldiserica (1), *Fimbriimonas ginsengisoli* Gsoil 348 of the Armatimonadetes (2), *Kiritimatiella glycovorans* L21-Fru-AB of the Kiritimatiellaeota (3), *Candidatus Cloacimonas acidaminovorans* str. Evry of the WWE1 candidate division (4), *Fibrobacter succinogenes* subsp. succinogenes S85 of the Fibrobacteres (5), *Gemmatimonas aurantiaca* T-27 of the Gemmatimonadetes (6), *Desulfurispirillum indicum* S5 of the Chrysiogenetes (7), and *Candidatus Babela massiliensis* 673862, which has been identified as the single representative of the TM6 phylum (8). Additionally, *Thermodesulfobium narugenese* DSM 14796 was removed as it was distant from the rest of the Firmicutes despite being labeled as one in the NCBI Taxonomy database. Work by other groups has demonstrated the distant relationship between *T. narugenese* and any known bacteria (9). The same was applied for *Coprothermobacter proteolyticus* DSM 5265, which was also labeled as a Firmicutes based on NCBI taxonomy, but it has been shown to not be a member (10). Some taxa were merged with others to reduce the phylogenetic complexity. The Tenericutes were included with Firmicutes and the Acidithiobacillia with the Deltaproteobacteria. The strain *bacterium* L21-Spi-D4 was included as a member of the Bacteroidetes based on its genome paper (11). The Desulfurellacae and Epsilonproteobacteria were included in the recently classified Campylobacterota (12, 13). Lastly, *Thermobaculum terrenum* ATCC BAA-798, not currently assigned a phylum in the NCBI Taxonomy database, was included as a member of the class Chloroflexi (phylum: Firmicutes) as it has been proposed to be a member (14).

References:

1. Mori,K., Yamaguchi,K., Sakiyama,Y., Urabe,T. and Suzuki,K.I. (2009) Caldisericum exile gen. nov., sp. nov., an anaerobic, thermophilic, filamentous bacterium of a novel bacterial phylum, Caldiserica phyl. nov., originally called the candidate phylum OP5, and description of Caldisericaceae fam. nov., Caldisericales ord. no. *Int. J. Syst. Evol. Microbiol.*, 10.1099/ijs.0.010033-0.
2. Hu,Z.-Y., Wang,Y.-Z., Im,W.-T., Wang,S.-Y., Zhao,G.-P., Zheng,H.-J. and Quan,Z.-X. (2014) The first complete genome sequence of the class *Fimbriimonadia* in the phylum Armatimonadetes. *PLoS One*, 10.1371/journal.pone.0100794.
3. Spring,S., Bunk,B., Sprï¿½er,C., Schumann,P., Rohde,M., Tindall,B.J. and Klenk,H.P. (2016) Characterization of the first cultured representative of Verrucomicrobia subdivision 5 indicates the proposal of a novel phylum. *ISME J.*, 10.1038/ismej.2016.84.
4. Pelletier,E., Kreimeyer,A., Bocs,S., Rouy,Z., Gyapay,G., Chouari,R., Rivière,D., Ganesan,A., Daegelen,P., Sghir,A., *et al.* (2008) "Candidatus Cloacamonas acidaminovorans": Genome sequence reconstruction provides a first glimpse of a new bacterial division. *J. Bacteriol.*, 10.1128/JB.01248-07.
5. Montgomery,L., Flesher,B. and Stahl,D. (1988) Transfer of Bacteroides succinogenes (Hungate) to Fibrobacter gen. nov. as Fibrobacter succinogenes comb. nov. and Description of Fibrobacter intestinalis sp. nov. *Int. J. Syst. Bacteriol.*, 10.1099/00207713-38-4-430.
6. Zhang,H., Sekiguchi,Y., Hanada,S., Hugenholtz,P., Kim,H., Kamagata,Y. and Nakamura,K. (2003) Gemmatimonas aurantiaca gen. nov., sp. nov., a Gram-negative, aerobic, polyphosphate-accumulating micro-organism, the first cultured representative of the new bacterial phylum Gemmatimonadetes phyl. nov. *Int. J. Syst. Evol. Microbiol.*, 10.1099/ijs.0.02520-0.
7. Rauschenbach,I., Narasingarao,P. and Häggblom,M.M. (2011) Desulfurispirillum indicum sp. nov., a selenate- and selenite-respiring bacterium isolated from an estuarine canal. *Int. J. Syst. Evol. Microbiol.*, 10.1099/ijs.0.022392-0.
8. Pagnier,I., Yutin,N., Croce,O., Makarova,K.S., Wolf,Y.I., Benamar,S., Raoult,D., Koonin,E. V. and La Scola,B. (2015) Babela massiliensis, a representative of a widespread bacterial phylum with unusual adaptations to parasitism in amoebae. *Biol. Direct*, 10.1186/s13062-015-0043-z.
9. Mori,K., Kim,H., Kakegawa,T. and Hanada,S. (2003) A novel lineage of sulfate-reducing microorganisms: Thermodesulfobiaceae fam. nov., Thermodesulfobium narugense, gen. nov., sp. nov., a new thermophilic isolate from a hot spring. *Extremophiles*, 10.1007/s00792-003-0320-0.
10. Nishida,H., Beppu,T. and Ueda,K. (2011) Whole-genome comparison clarifies close phylogenetic relationships between the phyla Dictyoglomi and Thermotogae. *Genomics*, 10.1016/j.ygeno.2011.08.001.
11. Ben Hania,W., Joseph,M., Bunk,B., Spröer,C., Klenk,H.P., Fardeau,M.L. and Spring,S. (2017) Characterization of the first cultured representative of a Bacteroidetes clade specialized on the scavenging of cyanobacteria. *Environ. Microbiol.*, 10.1111/1462-2920.13639.
12. Waite,D.W., Vanwonterghem,I., Rinke,C., Parks,D.H., Takai,K., Sievert,S.M., Simon,J., Campbell,B.J., Thomas,E., Woyke,T., *et al.* (2017) Comparative genomic analysis of the class Epsilonproteobacteria and proposed reclassification to Epsilonbacteraeota. *Front. Microbiol.*, 10.3389/fmicb.2017.00682.

13. Waite,D.W., Vanwonterghem,I., Rinke,C., Parks,D.H., Zhang,Y., Takai,K., Sievert,S.M., Simon,J., Campbell,B.J., Hanson,T.E., *et al.* (2018) Addendum: Comparative genomic analysis of the class Epsilonproteobacteria and proposed reclassification to Epsilonbacteraeota (phyl. nov.) [Front. Microbiol., 8, (2017), (682)] doi: 10.3389/fmicb.2017.00682. *Front. Microbiol.*, 10.3389/fmicb.2018.00772.
14. Kiss,H., Cleland,D., Lapidus,A., Lucas,S., Del Rio,T.G., Nolan,M., Tice,H., Han,C., Goodwin,L., Pitluck,S., *et al.* (2010) Complete genome sequence of "Thermobaculum terrenum" type strain (YNP1). *Stand. Genomic Sci.*, 10.4056/sigs.1153107.
